# Consumption of *Sutherlandia frutescens* by HIV-Seropositive South African Adults: An Adaptive Double-Blind Randomized Placebo Controlled Trial

**DOI:** 10.1371/journal.pone.0128522

**Published:** 2015-07-17

**Authors:** Douglas Wilson, Kathy Goggin, Karen Williams, Mary M. Gerkovich, Nceba Gqaleni, James Syce, Patricia Bartman, Quinton Johnson, William R. Folk

**Affiliations:** 1 Department of Internal Medicine, Edendale Hospital, Pietermaritzburg, University of KwaZulu-Natal, Durban, South Africa; 2 Health Services and Outcomes Research, Children’s Mercy Hospital and Clinics, University of Missouri-Kansas City Schools of Medicine and Pharmacy, Kansas City, Missouri, United States of America; 3 Biomedical and Health Informatics, University of Missouri-Kansas City School of Medicine, Kansas City, Missouri, United States of America; 4 AIK Innovations (Pty) Ltd, Durban University of Technology, Durban, South Africa; 5 School of Pharmacy, University of the Western Cape, Cape Town, South Africa; 6 Department of Internal Medicine Research Unit, Edendale Hospital, Pietermaritzburg, KwaZulu-Natal, South Africa; 7 George Campus, Nelson Mandela Metropolitan University, George, South Africa; 8 Department of Biochemistry, University of Missouri, Columbia, Missouri, United States of America; Glaxo Smith Kline, DENMARK

## Abstract

**Background:**

*Sutherlandia frutescens* (L.) R. Br. is widely used as an over the counter complementary medicine and in traditional medications by HIV seropositive adults living in South Africa; however the plant’s safety has not been objectively studied. An adaptive two-stage randomized double-blind placebo controlled study was used to evaluate the safety of consuming dried *S*. *frutescens* by HIV seropositive adults with CD4 T-lymphocyte count of >350 cells/μL.

**Methods:**

In Stage 1 56 participants were randomized to *S*. *frutescens* 400, 800 or 1,200 mg twice daily or matching placebo for 24 weeks. In Stage 2 77 additional participants were randomized to either 1,200 mg *S*. *frutescens* or placebo. In the final analysis data from Stage 1 and Stage 2 were combined such that 107 participants were analysed (54 in the *S*. *frutescens* 1,200 mg arm and 53 in the placebo arm).

**Results:**

*S*. *frutescens* did not change HIV viral load, and CD4 T-lymphocyte count was similar in the two arms at 24 weeks; however, mean and total burden of infection (BOI; defined as days of infection-related events in each participant) was greater in the *S*. *frutescens* arm: mean (SD) 5.0 (5.5) vs. 9.0 (12.7) days (p = 0.045), attributed to two tuberculosis cases in subjects taking isoniazid preventive therapy (IPT).

**Conclusion:**

A possible interaction between *S*. *frutescens* and IPT needs further evaluation, and may presage antagonistic interactions with other herbs having similar biochemical (antioxidant) properties. No other safety issues relating to consumption of *S*. *frutescens* in this cohort were identified.

**Trial Registration:**

ClinicalTrials.gov NCT00549523

## Background

The human immunodeficiency virus (HIV) pandemic has placed unprecedented demands on healthcare services in sub-Saharan Africa. In South Africa an estimated 5.26 million individuals (about 10% of the population) are living with HIV infection [1]. The South African government implemented the World Health Organization’s (WHO) recommendation that antiretroviral therapy (ART) be started when individuals’ CD4 T-lymphocyte count falls below 350 cells /μL [2]; however the average CD4 count of patients who start ART remains <150 cell/μL, with many patients deferring ART for a variety of reasons including lack of capacity within the South African public health service [3–6].

As in most countries, many South Africans use traditional, complementary and alternative medicines (TCAM) and/or consult with traditional health practitioners (THPs) [7–9]. More than 50% of adults in KwaZulu-Natal attending public sector facilities for ART initiation disclose using TCAM [10], and similar levels of TCAM use by HIV positive individuals may occur worldwide [11].

*Sutherlandia frutescens* is an indigenous southern African plant widely used to treat a variety of conditions including those associated with HIV infection (S1 Fig) [8, 12]. Teas, decoctions, capsules and tablets of the plant leaves are consumed and believed to influence immunity, stress, depression and wasting, all of which may significantly impact quality of life and therefore indirectly, progression of diseases such as HIV infection [13]. A variety of properties of *S*. *frutescens* and anecdotal reports suggest benefit in HIV seropositive patients [12]; however the plant is not registered by the South African Medicines Control Council (MCC) for specific indications.

No toxicity of *S*. *frutescens* was observed in either vervet monkeys at doses of up to 80 mg/kg dried powder over 3 months, or in healthy volunteers taking 400 mg dried powder twice daily for 24 weeks [14,15]. However, the safety and possible attributes of *S*. *frutescens* in HIV-seropositive adults are unknown. In response to repeated calls from the World Health Organization (WHO), the South African Development Community, and the South African government for controlled studies evaluating the safety and possible efficacy of TCAM practices [16, 17], we performed a double-blind randomized placebo-controlled clinical trial of *S*. *frutescens* in asymptomatic HIV-seropositive South Africans with >350 CD4 T-lymphocyte cells/μL.

## Methods

### Setting

HIV seropositive adults were recruited from clinics in the Edendale Hospital catchment area and evaluated at the Edendale Hospital Research Unit. This state-funded hospital serves 860,000 individuals living in urban and rural communities in the uMgungundlovu District of KwaZulu-Natal, South Africa. The demographic profile is similar to other high HIV prevalence settings in the province with an antenatal HIV seroprevalence of 42.2% in 2010 [18]. Close collaborations have developed between hospital staff and local THPs, especially relating to the management of HIV infection and tuberculosis.

### Study design and power

This Stage I/II study evaluated *S*. *frutescens* in healthy HIV seropositive adults using a double-blind, randomized, placebo-controlled design with a two-stage statistical selection theory design [19, 20]. In Stage 1, 56 participants were randomized to one of three doses of *S*. *frutescens* (400, 800, 1200 mg) or placebo, taken twice daily for 24 weeks. Interim analyses to determine which active study arm (i.e., 400, 800 or 1200 mg) should be continued in Stage 2 were conducted after at least twelve participants in each arm had completed the study. The most promising dose level was identified by comparing the number of participants in each arm with regard to clinical adverse events and evidence of treatment failure. Treatment failure was defined as follows: having either a decline in the primary outcomes of weight loss (>5% body mass) or CD4 T-lymphocyte (>20 cells/μL associated with percentage CD4 decline); having a score decrease of ≥ 20 points on any subscale score from the secondary outcome measure of the Medical Outcomes Study HIV Health Survey (MOS-HIV); or duration of infection more than 10 days of active infections during the 6 month trial (acquired on-study infections).

In the interim analysis changes in weight and CD4 count were similar across the arms. Unfavourable infection outcomes were observed in three of twelve participants in the 1200mg group, in six of fourteen participants in the placebo group, and in seven of twelve participants in the 400mg group. With respect to the MOS-HIV, failure differences were found between placebo and 1200 mg, favouring the 1200mg group. The study proceeded to Stage 2 in which placebo was compared with the highest dose evidenced to have no significant safety issues, have fewer failures than the placebo arm, and believed to be of the greatest potential benefit (i.e. 1,200 mg). The objectives in Stage 2 were therefore modified to assess the impact of *S*. *frutescens* on number, category (viral, bacterial, fungal, non-specific) and self-reported duration of infection events (burden of infection: BOI). Based on BOI data from Stage 1 (4 days versus 8 days average duration; SD 5 days), and assuming a two-tailed test and alpha of < .05, it was determined that a final sample of 60 completed subjects (30 completed subjects per arm) would be sufficient to compare burden of infection in Stage 2.

### Study product and randomization

A single batch of dried leaves of *S*. *frutescens* (L.) R. Br. was sourced from a commercial grower (Afriplex, Paarl, South Africa) and ground to a uniform powder. Four hundred milligrams of study product powder were packed into standard opaque 20 mm by 6 mm capsules by a GMP certified manufacturer (Ferlot Manufacturing Packaging (Pty) Ltd, Jeffreys Bay, South Africa). All study product was maintained at 18 to 24°C. The physicochemical properties of the study product were monitored annually by visual observation and high performance liquid chromatography measurement of Sutherlandioside B, canavanine and γ-amino butyric acid biomarkers. Placebo capsules were prepared with a mixture of lactulose, starch and a small amount of dried lettuce leaf powder to match the colour of *S*. *frutescens*. To further ensure blinding and match product odour a small amount of vanilla essence was added to the contents of both the placebo and *S*. *frutescens* capsules. Rigorous blinding procedures were developed to ensure that neither the participants nor study staff who worked directly with participants were able to link study product assignment with research subject. These practices conformed to the NCCAM/NIH Guidance on Natural Product Integrity and, substantively, the Guidelines for Complementary Medicines recently promulgated by the Republic of South Africa Department of Health and MCC [21,22]. At the baseline visit eligible participants were sequentially assigned a participant identification number (PIN) which had already been randomly linked to study arm and study product dispensing number by the off-site study statistician; randomization was stratified by gender. Study staff and participants did not have access to the randomization tables linking PIN to product dispensing number.

### Inclusion / exclusion criteria

HIV seropositive adults between the age of 21 and 64 years with CD4 T-lymphocyte count >350 cells/μL and HIV viral load >1,000 copies/ mL were eligible for enrolment into the study if: i) in good health without symptoms or signs of tuberculosis or other HIV-related conditions; ii) haematological and biochemical parameters were within normal range; iii) the resting electrocardiogram was normal; iv) traditional medicines had not been taken in the 28 days before screening; v) regular allopathic medications were not being taken (except for isoniazid and pyridoxine for the prevention of tuberculosis); vi) alcohol or recreational drugs were not being abused; and vii) not pregnant or breastfeeding. During Stage 1 of the study the HIV viral load inclusion criterion was successively lowered from >20,000 copies/mL to >1,000 copies/mL in order to enrol otherwise eligible participants and to meet enrolment targets. The full inclusion / exclusion criteria are shown in S1 Table.

### Development and validation of study questionnaires

In addition to the consent documents, standardized questionnaires to evaluate quality of life, psychological distress and depression were translated into isiZulu and validated using procedures already described [23,24].

### Study procedures

Stage 1 ran from May 2008 to July 2009 (interim analysis) and Stage 2 from August 2010 to December 2011 (study completion). Study procedures were conducted by registered nurses and medical practitioners. At baseline participants had a physical examination and electrocardiogram, had weight, height and skin fold thickness measured, and were prescribed a multivitamin for two months. To support adherence, study product was transferred into pill boxes by study staff, and adherence was measured by counting the number of capsules returned at each visit. Participants returned to the clinic at weeks 2, 4, 8, 12, 16, 20 and 24. Study questionnaires were administered by a study nurse at baseline and weeks 4, 12 and 24. Haematological and biochemical parameters were repeated at weeks 4, 8, 12 and 24, and the physical examination and electrocardiogram was repeated at weeks 12 and 24 and at other visits in response to symptoms. As consumption of *S*. *frutescens* has been anecdotally associated with systemic lupus erythematosis [25], serum anti-nuclear factor (ANF) was measured at baseline and weeks 12 and 24 and participants were screened for symptoms suggestive of vasculitis at each scheduled clinical review. Skin fold thickness and weight was repeated at every visit. Body mass index was calculated using the height measurement obtained at screening. Participants with unresolved medical issues were re-evaluated 4 weeks after exiting the study. Adverse events were graded using the standardized criteria developed by the NIH division of AIDS [26].

CD4 T-lymphocyte count and HIV viral load were repeated at weeks 12 and 24. Participants with a CD4 T-lymphocyte count of <350 cells/μL had a repeat count taken six weeks later; those found to have a second count of <350 cells/μL had the option of exiting the study and starting antiretroviral therapy.

Duration of those adverse events likely to be due to infection were collected prospectively in Stage 2. Study participants were asked to contact the study site at the onset of symptoms and were tracked regularly by telephone until resolution. Aetiology of infection events (viral, bacterial, fungal or protozoal) was determined by study physicians using standardized guidelines (S1 Text). The adverse event log was used similarly to infer aetiology of infection events occurring in Stage 1. In Stage 2 participants were offered isoniazid 300 mg daily with pyridoxine 50 mg daily as tuberculosis prevention therapy in accordance with current WHO recommendations and revised South African standard of care.

### Statistical analysis

Primary and secondary outcomes were analysed using an intention to treat analysis based on treatment assignment to groups. Analyses of the primary outcome, effect of treatment on infection (Mean BOI and Total Days BOI), were accomplished using independent t-tests using per protocol analyses. For secondary outcomes, profile plots for participants in each group across the 24 week observation period were generated. The secondary measures were analysed by fitting a mixed effects model to assess interaction effects of group over time (group and observation period as fixed effects) clustered within individual (participants as a random effect) on all continuous outcomes. Analyses were conducted using STATA 11.0 SE (StataCorp LP, College Station TX) to derive full maximum-likelihood and variance estimates with model assumptions confirmed through the analysis of residuals. Adherence to assigned study medication was calculated by subtracting the number of capsules returned from the number dispensed, dividing this figure by 6 multiplied by the number of days between visits, and expressing the result as a percentage. Differences in adherence between arms were compared using the Wilcoxon-Mann-Whitney test.

### Study oversight

The Biomedical Research Ethics Committee of the University of KwaZulu-Natal reviewed and approved the study protocol and amendments, and further approval to conduct the study was obtained from the KwaZulu-Natal Department of Health and the South African MCC, the Research Ethics Committees of Stellenbosch University, the University of the Western Cape, the University of Missouri and by the U.S. National Institutes of Health. All participants gave written informed consent in their home language. Compliance with the study protocol was evaluated by an independent study monitor and study data were regularly assessed by an independent Data Safety Monitoring Board at the University of Cape Town.

## Results

**Fig 1:**
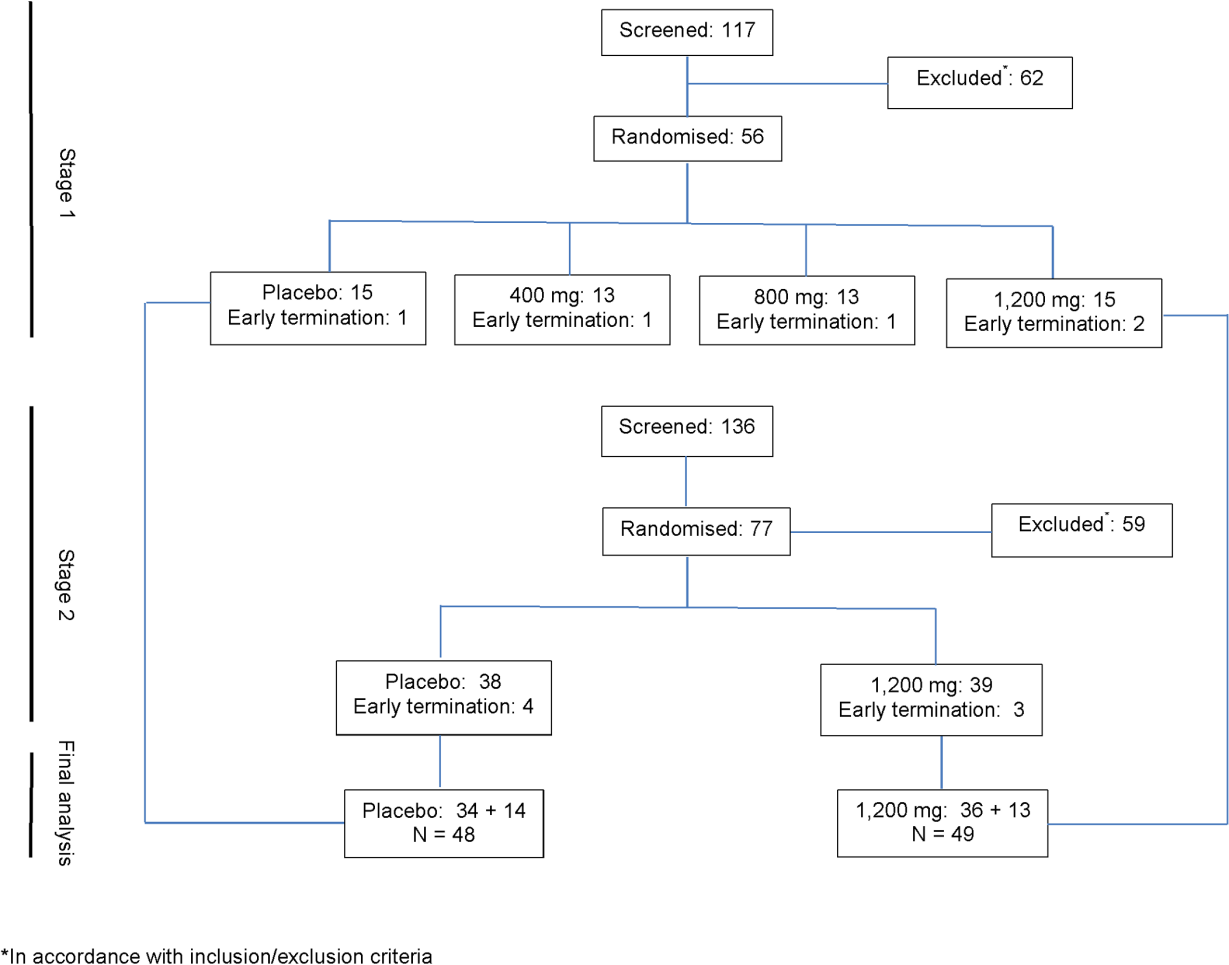
CONSORT diagram.

Participant screening, enrolment and randomization are shown in Fig 1. Fifty- six participants (50 women [89%] and 6 men [11%]) were enrolled into Stage 1, and 55 participants’ data were included in the interim analysis. In Stage 2, 77 participants were randomized to either *S*. *frutescens* 1,200 mg or placebo. At the completion of Stage 2 data from the 1,200 mg and placebo groups from both stages were combined for the final analysis. In total, 54 participants were randomized to the *S*. *frutescens* 1,200 mg arm and 53 to the placebo arm, with 49 (92%) participants in the *S*. *frutescens* 1,200 mg arm and 48 participants (90%) in the placebo arm completing the study. Thirty one participants in each arm were prescribed isoniazid preventive therapy (IPT).

### Baseline characteristics

Participant characteristics at baseline were similar in the two arms and are shown in Table 1; about 80% in each arm were female.

**Table 1:** Baseline demographic and clinical characteristics.

Characteristic	*S*. *frutescens* 1,200 mg	Placebo
	(N = 54)	(N = 53)
Female	81.1%	83.0%
	Mean (SD)	Mean (SD)
Age (years)	32.9 (8.3)	32.8 (8.3)
Education (grade)	10.5 (2.7)	10.1 (2.9)
Viral load (log_10_ copies/mL)	4.10 (0.60)	4.06 (0.63)
CD4 T-lymphocyte (cells/μL)	524.9 (142.9)	534.7 (134.8)
Haemoglobin (mg/dL)	12.8 (1.2)	12.9 (1.2)
MCV (fL)	88.6 (5.3)	88.9 (3.6)
Neutrophils (%)	50.6 (9.8)	50.3 (10.3)
Sodium (mmol/L)	136.5 (2.3)	136.5 (2.5)
Urea (mmol/L)	3.4 (1.3)	3.7 (1.2)
ALT (U/L)	21.8 (18.4)	19.2 (9.2)
Albumin (g/L)	40.3 (3.5)	40.8 (3.6)
Bilirubin (μmol/L)	6.8 (3.2)	6.9 (3.6)
Triglycerides mmol/L	1.0 (0.6)	0.9 (0.4)
Total cholesterol (mmol/L)	3.6 (0.9)	3.9 (0.9)

### Adherence

Adherence to study product was high (>80% in all participants who completed the study), and there were no significant differences in adherence across study arms (P-value 0.98).

### Changes in safety parameters

Biochemical and haematological parameters did not change significantly over the course of the study, irrespective of group (Table 2). P-values for change in glucose measurements were assessed as being not clinically significant. On serial electrocardiograms the PR interval, QRS duration and corrected QT interval (QTc) were similar in both arms (S2 Table).

**Table 2:** Changes in biochemical and hematological parameters over time in the combined Stage 1 and Stage 2 analysis *S*. *frutescens* 1,200 mg (N = 54) and placebo (N = 53).

Parameter	Baseline	Week 12	Week 24	P-value^*^
	Mean (SD)	Mean (SD)	Mean (SD)	
Urea mmol/L				0.541
*S*. *frutescens*	3.4 (1.0)	3.4 (1.1)	3.7 (1.1)	
Placebo	3.7 (1.2)	3.6 (1.3)	3.8 (1.1)	
Sodium mmol/L				0.247
*S*. *frutescens*	136.6 (2.2)	136.2 (2.5)	136.4 (2.8)	
Placebo	136.5 (2.5)	136.4 (2.2)	137.1 (2.6)	
Potassium mmol/L				0.671
*S*. *frutescens*	4.1 (0.4)	4.0 (0.4)	4.2 (0.4)	
Placebo	4.0 (0.3)	4.0 (0.5)	4.1 (0.4)	
Bicarbonate mmol/L				0.961
*S*. *frutescens*	25.4 (2.1)	24.6 (2.6)	25.7 (3.0)	
Placebo	25.3 (2.8)	25.2 (2.5)	25.9 (3.1)	
Alanine aminotransferase U/L				0.903
*S*. *frutescens*	21.7 (18.2)	23.1 (20.9)	23.4 (20.8)	
Placebo	19.2 (9.2)	23.4 (18.8)	20.4 (13.2)	
Alkaline phosphatase U/L				0.493
*S*. *frutescens*	65.9 (20.2)	66.7 (34.8)	67.1 (25.2)	
Placebo	67.0 (18.9)	67.3 (18.4)	67.0 (18.9)	
Total bilirubin μmol/L				0.742
*S*. *frutescens*	6.8 (3.2)	6.4 (2.6)	6.7 (3.8)	
Placebo	6.9 (3.6)	6.9 (3.0)	7.2 (4.2)	
Albumin g/L				0.606
*S*. *frutescens*	40.4 (3.5)	39.8 (3.3)	40.4 (3.1)	
Placebo	40.8 (3.6)	40.5 (3.8)	41.5 (2.9)	
Calcium mmol/L				0.288
*S*. *frutescens*	2.24 (0.09)	2.21 (0.09)	2.22 (0.08)	
Placebo	2.25 (0.09)	2.21 (0.08)	2.21 (0.09)	
Inorganic phosphate mmol/L				0.069
*S*. *frutescens*	1.1 (0.2)	1.1 (0.2)	1.0 (0.2)	
Placebo	1.0 (0.2)	1.1 (0.2)	1.0 (0.2)	
Random glucose mmol/L				0.048
*S*. *frutescens*	4.5 (0.6)	5.0 (1.0)	4.8 (0.8)	
Placebo	4.7 (0.8)	5.0 (0.9)	4.6 (0.7)	
Creatine kinase U/L				0.584
*S*. *frutescens*	73.7 (12.8)	67.4 (11.9)	68.5 (9.3)	
Placebo	74.4 (13.1)	72.2 (13.8)	69.8 (11.1)	
Total cholesterol mmol/L				0.812
*S*. *frutescens*	3.59 (0.93)	3.45 (0.80)	3.41 (0.80)	
Placebo	3.92 (0.92)	3.72 (0.93)	3.69 (1.01)	
HLD mmol/L				0.821
*S*. *frutescens*	1.04 (0.28)	1.01 (0.29)	1.04 (0.38)	
Placebo	1.05 (0.25)	0.97 (0.25)	1.04 (0.24)	
LDL mmol/L				0.291
*S*. *frutescens*	2.09 (0.77)	1.94 (0.66)	1.90 (0.67)	
Placebo	2.39 (0.77)	2.25 (0.73)	2.28 (0.78)	
Triglycerides mmol/L				0.948
*S*. *frutescens*	0.95 (0.59)	0.95 (0.50)	0.95 (0.47)	
Placebo	0.93 (0.37)	0.97 (0.37)	0.99 (0.64)	
Hemoglobin g/dL	12.8 (1.1)	12.8 (1.3)	12.7 (1.3)	0.105
*S*. *frutescens*				
Placebo	12.8 (1.2)	13.8 (1.3)	13.2 (1.3)	
Mean cell volume fL				0.716
*S*. *frutescens*	88.6 (5.3)	88.0 (5.2)	88.7 (5.1)	
Placebo	88.8 (3.6)	88.7 (3.6)	87.8 (9.2)	
Platelets ×10^9^/L				0.354
*S*. *frutescens*	301 (80)	297 (86)	288 (64)	
Placebo	285 (60)	292 (78)	285 (71)	
Leucocyte count ×10^9^/L				0.699
*S*. *frutescens*	5.8 (1.7)	5.5 (1.6)	5.3 (1.4)	
Placebo	5.6 (1.7)	5.3 (1.3)	5.3 (1.5)	
Lymphocyte count ×10^9^/L				0.881
*S*. *frutescens*	2.1 (0.7)	2.0 (0.7)	1.9 (0.6)	
Placebo	2.1 (0.6)	2.0 (0.5)	1.9 (0.6)	
Neutrophil count ×10^9^/L				0.634
*S*. *frutescens*	3.0 (1.2)	2.9 (1.2)	2.6 (0.9)	
Placebo	2.9 (1.3)	2.7 (1.0)	2.7 (1.2)	
Eosinophil count ×10^9^/L				0.087
*S*. *frutescens*	0.18 (0.16)	0.20 (0.19)	0.23 (0.22)	
Placebo	0.18 (0.15)	0.18 (0.13)	0.20 (0.17)	
Monocyte count ×10^9^/L				0.234
*S*. *frutescens*	0.33 (0.13)	0.30 (0.11)	0.29 (0.10)	
Placebo	0.28 (0.08)	0.30 (0.08)	0.28 (0.08)	
Basophil count ×10^9^/L				0.899
*S*. *frutescens*	0.03 (0.01)	0.02 (0.01)	0.03 (0.01)	
Placebo	0.03 (0.02)	0.03 (0.02)	0.03 (0.02)	

Abbreviations: HDL (high density lipoprotein); LDL (low density lipoprotein)

*P-value for interaction effect of groups over time.

### Adverse events and serious adverse events

Adverse events classified by system (S3 Table) were evenly distributed between the two arms, as were adverse events caused by infection (Table 3). Two serious adverse events occurred, both in participants on the *S*. *frutescens* arm: one participant had a positive urine pregnancy test followed two weeks later by spontaneous miscarriage; and one participant was hospitalized with shingles and early varicella dissemination that resolved completely with intravenous acyclovir. Neither was judged to be related to the study product. Five participants on the *S*. *frutescens* arm and one participant on the placebo arm developed a positive anti-nuclear factor during the study period, two of who reverted to negative. Two participants with a positive ANF at baseline tested negative during the study period. None of the participants developed vasculitis, and none needed to start antiretroviral therapy within 24 weeks of the baseline visit.

**Table 3:** Infection outcomes in the combined Stage 1 and Stage 2 analysis *S*. *frutescens* 1,200 mg (N = 54) and placebo (N = 53).

Infections	*S*. *frutescens* 1,200 mg	Placebo	P-value
Number of Infections			0.372
None	17 (33.3%)	22 (42.3%)	
One	18 (35.3%)	17 (32.7%)	
Two	10 (19.6%)	8 (15.4%)	
Three	3 (5.9%)	3 (5.8%)	
Four	3 (5.9%)	2 (3.8%)	
Type of Infection			0.784
Viral	33 (53.2%)	26 (51.0%)	
Bacterial	24 (38.7%)	18 (35.3%)	
Fungal	4 (6.5%)	5 (9.8%)	
Protozoal	1 (1.6%)	2 (3.9%)	
Non-specific	-	-	
Burden of Infection			
Mean BOI	9.0 (12.7)	5.0 (5.5)	0.045
Total BOI	18.2 (25.4)	9.0 (12.7)	0.065

Abbreviation: BOI (burden of infection)

### Burden of infection

Total and mean BOI was greater in the *S*. *frutescens* arm (Table 3), with statistical significance being due to two participants who were diagnosed with tuberculosis while taking IPT. No other cases of tuberculosis occurred on the study.

### Efficacy endpoints

Mean body mass index and skin fold thickness did not change significantly in the two arms (S4 Table). Both CD4 T-lymphocyte count and HIV viral load decreased over the duration of the study, but the magnitude of the change was not significantly different between the two arms (Table 4).

**Table 4:** Changes in CD4 T-lymphocyte count and HIV viral load over time in the combined Stage 1 and Stage 2 analysis *S*. *frutescens* 1,200 mg (N = 54) and placebo (N = 53).

HIV Measures	Baseline	Week 12	Week 24	P-value^*^
	Mean (SD)	Mean (SD)	Mean (SD)	
CD4 count T-lymphocyte cells/μL				0.632
*S*. *frutescens*	524 (142)	496 (174)	474 (155)	
Placebo	535 (135)	529 (167)	517 (186)	
Viral Load log_10_ copies/mL				0.829
*S*. *frutescens*	4.08 (0.60)	4.05 (0.65)	3.91 (0.74)	
Placebo	4.06 (0.63)	3.84 (0.79)	3.87 (0.69)	

*P-value for interaction effect of groups over time.

### Quality of life scores

Mean scores for depression, perceived stress and most HIV symptoms (MOS-HIV) did not differ between the two arms of the study after controlling for baseline variability (S5 Table). There were however, statistically significant interaction effects (differences between groups over time) for the Social (P-value < 0.01) and Mental Health (P-value 0.03) measures, although the differences were of small magnitude and appeared to reflect slightly different changes in trajectory from baseline values.

## Discussion

This is the first study to rigorously evaluate an African traditional and complementary medicine in adults living with HIV infection, using well established ethical and regulatory norms. This study responds to calls by the WHO and others to rigorously evaluate African traditional medicines in the context in which they are used with the knowledge and support of THPs [7–12, 15–17, 27–29], and provide a precedent and guidance for the conduct of future clinical trials of African TCAM practices. When compared to placebo *S*. *frutescens* did not impact CD4 T-lymphocyte count, however duration of secondary infection in participants consuming *S*. *frutescens* 1,200 mg was longer than that observed in those on placebo due to two participants developing tuberculosis while taking *S*. *frutescens*, despite taking isoniazid. The possible interaction that reduces the efficacy of IPT requires further evaluation. One possible explanation for interference between *S*. *frutescens* and IPT might be antioxidant/radical quenching properties of *S*. *frutescens* [30–33] which could block the proposed mechanisms of action of isoniazid [34]. This requires further study but, if experimentally supported, could substantially advance strategies for the prevention of tuberculosis and reduce development of drug resistance in regions where TCAM use occurs concurrently with IPT.

This study has several limitations. Asymptomatic HIV-positive adults were enrolled at a single site in KwaZulu-Natal. Inclusion of participants with more advanced HIV disease may have changed study findings in unpredictable ways; however known interactions between *S*. *frutescens* and antiretroviral medications precluded their inclusion in this study [27, 28]. Inclusion of participants with diabetes and/or adrenal imbalances may have revealed other effects of *S*. *frutescens* but was outside the scope of this study [35, 36]. The majority of participants in this study were women; this gender bias for enrolment in clinical studies is a limiting factor, but has been observed in multiple other contexts. Further, different ways of preparing *S*. *frutescens* for consumption may alter the chemistry and availability of any bioactive components.

Based on data from Stage 1, duration of infection and BOI were used as secondary endpoints in Stage 2. Data on duration of infection therefore needed to be retrospectively collected from Stage 1 source documents using adverse event start/stop dates. Monitoring for infectious events was less intense in Stage 1 and the duration of infection relied on participant recollection at scheduled study visits. However these limitations would have equally affected both arms of the study in Stage 1 participants whose data were carried forward into the final analysis and data on infection events requiring hospitalization or antibiotic treatment would have been captured. Data from this study suggests that *S*. *frutescens* at doses of up to 2,400 mg daily does not causes significant adverse effects in HIV seropositive adults; however possible interactions between *S*. *frutescens* and isoniazid require further study.

## Supporting Information

S1 CONSORT Checklist CONSORT Checklist. (PDF)

S1 Fig Photograph of *S*. *frutescens* taken at the Kirstenbosch National Botanical Garden, Cape Town (PDF)

S1 Table Inclusion and exclusion criteria (DOCX)

S1 Text Management of data on duration of infection (PDF)

S2 Table Baseline and Week 24 electrocardiogram intervals in the Stage 2 analysis *S*. *frutescens* 1,200 mg (N = 36) and placebo (N = 34) (DOCX)

S3 Table Comparison of adverse events by system in the Stage 2 analysis *S*. *frutescens* 1,200 mg (N = 39) and placebo (N = 38) (DOCX)

S4 Table Weight and body fat over time in the combined Stage 1 and Stage 2 analysis *S*. *frutescens* 1,200 mg (N = 54) and placebo (N = 53) (DOCX)

S5 Table Quality of life scores in the combined Stage 1 and Stage 2 analysis *S*. *frutescens* 1,200 mg (N = 54) and placebo (N = 53) (DOCX)

S1 Protocol Trial Protocol. (PDF)
