# Supplementary material for: Consumption of Sutherlandia frutescens by HIV-Seropositive South African Adults: An Adaptive Double-Blind Randomized Placebo Controlled Trial
Source: PLoS One. 2015 Jul 17;10(7):e0128522. doi: 10.1371/journal.pone.0128522 (PMC4506018; doi:10.1371/journal.pone.0128522)
Supplement: S1 Fig — (PDF) [file pone.0128522.s002.pdf]

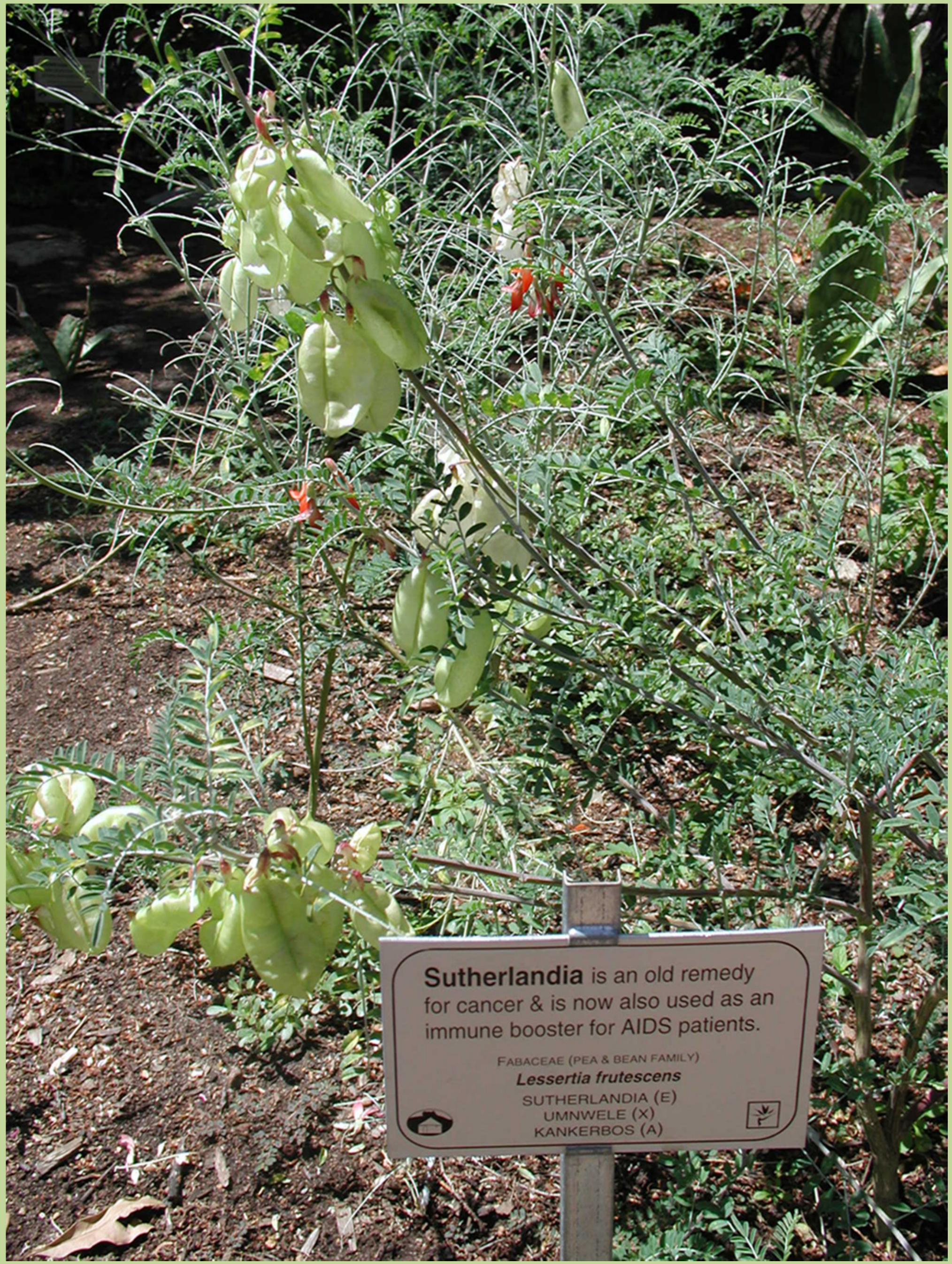

S1 Fig.: Photograph of *S. frutescens* taken at the Kirstenbosch National Botanical Garden, Cape Town
