# Supplementary material for: Consumption of Sutherlandia frutescens by HIV-Seropositive South African Adults: An Adaptive Double-Blind Randomized Placebo Controlled Trial
Source: PLoS One. 2015 Jul 17;10(7):e0128522. doi: 10.1371/journal.pone.0128522 (PMC4506018; doi:10.1371/journal.pone.0128522)
Supplement: S1 Table — (DOCX) [file pone.0128522.s003.docx]

**S1 Table**: Inclusion and exclusion criteria

*Inclusion Criteria*

1. Age ≥21 years and <65 years

2. HIV-1 infection documented in the medical record by two different rapid tests for HIV-1 antibodies

3. CD4 count >350 cells/μL

4. Viral load ≥1,000 copies/mL

5. Normal haematological function (haemoglobin >10.0 g/dL, absolute neutrophil count >1.0x109, eosinophil count <2.4x109, platelet count >100x1011)

6. Absence of clinically significant renal disease: a). serum creatinine <140 μmol/L; b). glomerular filtration rate ≥ 60 mL/min calculated using the formula of Cockroft and Gault;* and c). absence of haematuria and/or ≥1+ proteinuria on urine dipstick.

7. Normal liver function (INR <1.5, bilirubin <1.5x normal, ALT <2x normal, ALP <2x normal)

8. Random glucose <11.1 mmol/L

9. Normal electrocardiogram.**

10. Regular attendance at the Wellness Clinic for at least 4 visits

11. Cognitive capacity sufficient to provide informed consent

12. Has not taken any traditional medication for 28 days prior to screening

*Exclusion Criteria*

1. Any AIDS-defining diagnosis

2. Weight loss >5% of body weight within the preceding six months

3. Other features of undiagnosed tuberculosis (including cough, fatigue, drenching night sweats and abnormal chest radiograph).

4. Any other significant disease (e.g. active tuberculosis, hypertension, diabetes mellitus and other endocrine disorders, peptic ulcer disease, gastrointestinal malabsorption, psychiatric illness) either newly diagnosed or controlled by medication.

5. Use of any allopathic medication other than isoniazid for tuberculosis prophylaxis.

6. Use of traditional medicines within the past 28 days.

7. Prior or current use of antiretroviral therapy

8. History of allergic conditions (e.g. asthma, eczema, urticaria requiring medical therapy on more than one occasion) or drug allergy/hypersensitivity.

9. Either history or family history of autoimmune disease (e.g. systemic lupus erythmatosis, Guillian Barre, haemolytic anaemia)

10. Alcohol use of >7 units per week or 3 units per occasion, tobacco use greater than 10 cigarettes per day or description of recreational drug use within the past 6 months

11. Pregnancy or breast-feeding

12. Women of childbearing potential who are sexually active and not using medically accepted dual contraceptive measures, as judged by the investigator***

13. Participation in a clinical study of any investigational product 1 month prior to the screening visit

Notes: *Formula of Cockroft and Gault: glomerular filtration rate = (140 – age) x weight (kg) / 0.82 x plasma creatinine (μmol/L). In women plasma creatinine is multiplied by 0.85 instead of 0.82)

**Acceptable variants: first degree heart block with normal QRS duration and normal axis; QTc ≤0.44 sec; bradycardia and/or voltage criteria for left ventricular hypertrophy in precordial leads in physically fit individuals with normal cardiac examination.

***Dual contraceptive measures: consistent use of the male condom with use of another recognized contraceptive method (female condom, intramuscular medroxyprogesterone or norethisterone, combined oral contraceptive pill, intrauterine device). Complete abstinence from sexual intercourse and the stated intention to continue abstinence will be accepted as the alternative to dual contraception.
