# Supplementary material for: Consumption of Sutherlandia frutescens by HIV-Seropositive South African Adults: An Adaptive Double-Blind Randomized Placebo Controlled Trial
Source: PLoS One. 2015 Jul 17;10(7):e0128522. doi: 10.1371/journal.pone.0128522 (PMC4506018; doi:10.1371/journal.pone.0128522)
Supplement: S1 Text — (PDF) [file pone.0128522.s004.pdf]

Detection of infection-related adverse events (data integrity):

In order to optimize detection of infection-related adverse events participants will be asked at SMS a 'Please call me' message to the study mobile telephone at the onset of any adverse event. Alternatively, participants can telephone or visit the study site. Details of the symptoms and treatment received will be elicited by the research staff and documented in the adverse event follow-up log (appendix 1). After the initial report of the adverse event the study staff will call the participant every 2 days to follow-up on progression of the adverse event until resolution. The date of onset and resolution of the adverse event will be documented in the follow-up log during the initial and follow-up calls.

Documentation of the adverse event:

Participants have the option of consulting one of the study clinicians for management of the adverse event. The diagnosis for the adverse event will be recorded in the adverse event section of the source documents, along with medication prescribed in the concomitant medication section. The clinician will indicate if the adverse event was due to an infectious or non -infectious cause; and if infectious whether the cause was viral, bacterial, fungal or non-specific (see adverse event page).

If the participant has not attended for an unscheduled visit the study clinician will review the notes made in the adverse event log, and record the diagnosis for the adverse event adverse event section of the source documents, along with medication prescribed in the concomitant medication section. The participant will be encouraged to bring in the hand-held clinic card for independent verification of the adverse event and concomitant medications. The clinician will indicate if the adverse event was due to an infectious or non -infectious cause.

The start and stop dates for the adverse event will be captured from the follow-up log and recorded in the adverse event section of the source documents.

In order to achieve as much diagnostic certainty as possible in the determination of whether or not the adverse event was due to an infection case definitions will be used where possible (appendix 2), or the case will be discussed with an infectious diseases (ID) consultant. When telephonic consultation cannot resolve the diagnosis the source documents will be photocopied and with the clinician's diagnosis screened out. The PI (DW) and the Medical Advisor (HD) will review the adverse event notes and data independently, blinded to the clinician's final diagnosis and code whether or not the adverse event was due to an infectious or non -infectious cause. Discrepant outcomes will be resolved by consensus, if necessary by involving GM.

Infectious adverse events will be coded as viral, bacterial or fungal, and the basis of the diagnosis will be indicated (appendix 3).

Compiled by: D Wilson

Dr D Wilson
Principal Investigator __________________
Effective Date December 2009

REVIEW DATE : December 2010

Appendix 1: Adverse event follow-up log

Study number .... ☐☐☐☐

Patient initials ……………………………………………………………… ☐☐☐

Adverse event no.	Symptoms	Treatment
	Start date: DD / MM / YYYY Stop date: DD / MM / YYYY	Check when diagnosis captured as adverse event ☐
	Start date: DD / MM / YYYY Stop date: DD / MM / YYYY	Check when diagnosis captured as adverse event ☐

Appendix 2: Case definitions for infectious adverse events occurring in Phase 1

Key reference: Standard treatment guidelines and essential medicines list (EDL). Essential drug programme, South African Department of Health (2008)

1/ Diarrhoea

Viral: Watery high volume diarrhoea without blood or mucous (small bowel) with fevers and chills [Note fevers and chills essential to differentiate from food poisoning]

Bacterial: Low volume frequent diarrhoea with fever and chills and/or tenesmus and/or blood and/or mucous in the stool

Parasitic: Diarrhoea lasting for more than 7 days responding to metronidazole (giardiasis or amoebiaisis)

Reference: EDL page 23 - 27, 168

2/ Urinary tract infection

Bacterial: Dysuria for more than 24 hours with leucocytes and/or nitrates on urine dipstick

Reference: EDL page 136

3/ Upper respiratory tract infection

Viral: Nasal stuffiness and throat irritation (pain)
[Not associated with nasal itchiness (allergic rhinitis); may be associated with fever; initially nasal discharge, later purulent]

Reference: EDL page 280

Bacterial sinusitis: Deterioration of a common cold after 5-7 days; with purulent nasal discharge especially if unilateral; and pain and tenderness over one or more sinuses.

Tonsillitis and pharyngitis: A painful red throat and/or enlarged inflamed tonsils. Yellow exudates may be present. Tender anterior cervical lymphadenopathy may be present. Viruses are the cause in the majority of cases. However, streptococcal pharyngitis/tonsillitis may cause local suppurative complications as well as rheumatic fever, which can cause serious heart disease. Antibiotics to eradicate streptococci should be given to patients with pharyngitis/tonsillitis who are at risk for rheumatic fever **unless** one of the following features of viral infection is present (do **not** give antibiotics if these are present): runny nose or cough or a rash [suggests viral aetiology]

[Note: Code as viral or bacterial using the above definition]

Reference: EDL page 316

3/ Otitis media

Bacterial: Inflammation of the middle ear characterised by: pain; <u>and</u> loss of the normal light reflex of the eardrum; <u>or</u> red bulging eardrum; » drum perforation;

Reference: EDL page 312

4/ Fungal skin rash

Tinea corporis (ring worm): itchy ringlike patches <u>and</u> raised borders <u>and</u> patches slowly grow bigger

Cutaneous candidiasis: Appears moist (weeping) <u>and</u> with red raw-looking patches.

[Note: May have peripheral white pustules and scales or have clear edges. Common sites: under the breasts; perineum; axilla; nail folds; groin]

Reference: EDL page 81

5/ Vaginal candidiasis

Fungal: Abnormal vaginal discharge <u>with</u> itching

6/ Vaginal discharge

Bacterial / fungal / protozoal: Abnormal vaginal discharge <u>with</u> dysuria <u>or</u> vaginal itching or burning

[Note: If not sexually active over the past 3 months code as protozoal and treat for *Trichmonas vaginalis*. If sexually active within the past 3 months code as bacterial and treat syndromically]

Reference: EDL page 81

7/ Male urithritis syndrome

Bacterial: Urethral discharge of dysuria

[Note: If symptoms persist for more than 7 days after treatment, code as protozoal and treat for *Trichmonas vaginalis.*]

Reference: EDL page 208

8/ Skin abscess

Bacterial: Skin becomes swollen <u>and</u> red <u>and</u> tender and hot

Reference: EDL page 75

Additional case definitions for other common infectious conditions

9/ Bronchitis:

Viral: Non-productive cough with burning retrosternal chest pain

Bacterial: productive cough with yellow or greenish sputum

[Note: Antibiotics may be considered for HIV positive patients because of the higher incidence of bacterial lower respiratory tract infections in this subgroup]

Reference: EDL page 281

10/ Pneumonia

Bacterial: Fever, often with sudden onset and with rigors, **and** cough, which becomes productive of rusty brown or yellow-green sputum, **and** pleuritic type chest pain **and** tachypnoea **and** any one of the following signs - crackles or crepitations, bronchial breath sounds, or a pleural rubbing sound or signs of a pleural effusion or an abnormal chest radiograph

Reference: EDL: Page 282

Additional Resource:

WHO case definitions for surveillance and revised clinical staging and immunological classification of HIV-related diseases in adults and children (2006) Annex 1 - shown below.

ANNEX 1. PRESUMPTIVE AND DEFINITIVE CRITERIA FOR RECOGNIZING HIV-RELATED CLINICAL EVENTS IN ADULTS (15 YEARS OR OLDER) AND CHILDREN (YOUNGER THAN 15 YEARS) WITH CONFIRMED HIV INFECTION

Adults (15 years or older)

CLINICAL EVENT	CLINICAL DIAGNOSIS	DEFINITIVE DIAGNOSIS
CLINICAL STAGE 1		
Asymptomatic	No HIV-related symptoms reported and no signs on examination	Not applicable
Persistent generalized lymphadenopathy	Painless enlarged lymph nodes >1 cm in two or more non-contiguous sites (excluding inguinal) in the absence of known cause and persisting for three months or more	Histology
CLINICAL STAGE 2		
Moderate unexplained weight loss (<10% of body weight)	Reported unexplained involuntary weight loss in pregnancy failure to gain weight	Documented weight loss <10% of body weight
Recurrent upper respiratory tract infections (current event plus one or more in last six-month period)	Symptom complex, such as unilateral face pain with nasal discharge (sinusitis), painful inflamed eardrum (otitis media) or tonsillopharyngitis without features of viral infection (such as coryza or cough)	Laboratory studies where available, such as culture of suitable body fluid
Herpes zoster	Painful vesicular rash in dermatomal distribution of a nerve supply, does not cross the midline	Clinical diagnosis

CLINICAL EVENT	CLINICAL DIAGNOSIS	DEFINITIVE DIAGNOSIS
Angular cheilitis	Splits or cracks at the angle of the mouth not due to iron or vitamin deficiency, usually respond to antifungal treatment	Clinical diagnosis
Recurrent oral ulcerations (two or more episodes in last six months)	Aphthous ulceration, typically painful with a halo of inflammation and a yellow-grey pseudomembrane	Clinical diagnosis
Papular pruritic eruption	Papular pruritic lesions, often with marked post-inflammatory pigmentation	Clinical diagnosis
Seborrhoeic dermatitis	Itchy scaly skin condition, particularly affecting hairy areas (scalp, axillae, upper trunk and groin)	Clinical diagnosis
Fungal nail infections	Paronychia (painful red and swollen nail bed) or onycholysis (separation of the nail from the nail bed) of the fingernails (white discoloration – especially involving proximal part of nail plate – with thickening and separation of the nail from the nail bed)	Fungal culture of the nail or nail plate material

CLINICAL EVENT	CLINICAL DIAGNOSIS	DEFINITIVE DIAGNOSIS
CLINICAL STAGE 3		
Unexplained severe weight loss (more than 10% of body weight)	Reported unexplained involuntary weight loss (>10% of body weight) and visible thinning of face, waist and extremities with obvious wasting or body mass index <18.5 kg/m^2; in pregnancy, the weight loss may be masked	Documented loss of more than 10% of body weight
Unexplained chronic diarrhoea for longer than one month	Chronic diarrhoea (loose or watery stools three or more times daily) reported for longer than one month	Three or more stools observed and documented as unformed, and two or more stool tests reveal no pathogens
Unexplained persistent fever (intermittent or constant and lasting for longer than one month)	Fever or night sweats for more than one month, either intermittent or constant with reported lack of response to antibiotics or antimalarial agents, without other obvious foci of disease reported or found on examination; malaria must be excluded in malarious areas	Documented fever >37.5°C with negative blood culture, negative Ziehl-Nielsen stain, negative malaria slide, normal or unchanged chest X-ray and no other obvious focus of infection
Oral candidiasis	Persistent or recurring creamy white curd-like plaques that can be scraped off (pseudomembranous) or red patches on tongue, palate or lining of mouth, usually painful or tender (erythematous form)	Clinical diagnosis

CLINICAL EVENT	CLINICAL DIAGNOSIS	DEFINITIVE DIAGNOSIS
Oral hairy leukoplakia	Fine white small linear or corrugated lesions on lateral borders of the tongue that do not scrape off	Clinical diagnosis
Pulmonary tuberculosis (current)	Chronic symptoms: (lasting more than 2–3 weeks) cough, haemoptysis, shortness of breath, chest pain, weight loss, fever, night sweats, and no clinical evidence of extrapulmonary disease<br><br>Discrete peripheral lymph node *M. tuberculosis* infection (especially cervical) is considered a less severe form of extrapulmonary tuberculosis	One or more sputum smear positive for acid-fast bacilli and/or radiographic abnormalities consistent with active tuberculosis and/or culture positive for *Mycobacterium*
Severe bacterial infection (such as pneumonia, meningitis, empyema, pyomyositis, bone or joint infection, bacteraemia and severe pelvic inflammatory disease)	Fever accompanied by specific symptoms or signs that localize infection and response to appropriate antibiotic	Isolation of bacteria from appropriate clinical specimens (usually sterile sites)
Acute necrotizing ulcerative gingivitis or necrotizing ulcerative periodontitis	Severe pain, ulcerated gingival papillae, loosening of teeth, spontaneous bleeding, bad odour and rapid loss of bone and/or soft tissue	Clinical diagnosis

CLINICAL EVENT	CLINICAL DIAGNOSIS	DEFINITIVE DIAGNOSIS
Unexplained anaemia (<8 g/dl), neutropaenia (<0.5 × 10^9 per litre) or chronic (more than one month) thrombocytopaenia (<50 × 10^9 per litre)	Not presumptive clinical diagnosis	Diagnosed on laboratory testing and not explained by other non-HIV conditions; not responding to standard therapy with haematinics, antimalarial agents or anthelmintic agents as outlined in relevant national treatment guidelines, WHO Integrated Management of Childhood Illness guidelines or other relevant guidelines
CLINICAL STAGE 4		
HIV wasting syndrome	Unexplained involuntary weight loss (>10% baseline body weight), with obvious wasting or body mass index <18.5<br><br>PLUS<br><br>unexplained chronic diarrhoea (loose or watery stools three or more times daily) reported for longer than one month<br><br>OR<br><br>reports of fever or night sweats for more than one month without other cause and lack of response to antibiotics or antimalarial agents; malaria must be excluded in malarious areas	Documented weight loss >10% of body weight<br><br>PLUS<br><br>two or more unformed stools negative for pathogens<br><br>OR<br><br>documented temperature of >37.5°C with no other cause of disease, negative blood culture, negative malaria slide and normal or unchanged chest X-ray

CLINICAL EVENT	CLINICAL DIAGNOSIS	DEFINITIVE DIAGNOSIS
Pneumocystis pneumonia	Dyspnoea on exertion or nonproductive cough of recent onset (within the past three months), tachypnoea and fever<br><br>AND<br><br>Chest X-ray evidence of diffuse bilateral interstitial infiltrates<br><br>AND<br><br>No evidence of bacterial pneumonia; bilateral crepitations on auscultation with or without reduced air entry	Cytology or immunofluorescent microscopy of induced sputum or bronchoalveolar lavage or histology of lung tissue
Recurrent severe bacterial pneumonia	Current episode plus one or more previous episodes in the past six months; acute onset (<2 weeks) of severe symptoms (such as fever, cough, dyspnoea, and chest pain) PLUS new consolidation on clinical examination or chest X-ray; response to antibiotics	Positive culture or antigen test of a compatible organism
Chronic herpes simplex virus infection (orolabial, genital or anorectal) of more than one month or visceral infection of any duration	Painful, progressive anogenital or orolabial ulceration; lesions caused by recurrence of herpes simplex virus infection and reported for more than one month. History of previous episodes. Visceral herpes simplex virus requires definitive diagnosis	Positive culture or DNA (by polymerase chain reaction) of herpes simplex virus or compatible cytology or histology

CLINICAL EVENT	CLINICAL DIAGNOSIS	DEFINITIVE DIAGNOSIS
Oesophageal candidiasis	Recent onset of retrosternal pain or difficulty on swallowing (food and fluids) together with oral *Candida*	Macroscopic appearance at endoscopy or bronchoscopy, or by microscopy or histology
Extrapulmonary tuberculosis	Systemic illness (such as fever, night sweats, weakness and weight loss). Other evidence for extrapulmonary or disseminated tuberculosis varies by site, such as pleura, pericardia, meninges, mediastinum or abdominal<br><br>Discrete peripheral lymph node *Mycobacterium tuberculosis* infection (especially cervical) is considered a less severe form of extrapulmonary tuberculosis	*M. tuberculosis* isolation or compatible histology from appropriate site or radiological evidence of miliary TB (diffuse uniformly distributed small miliary shadows or micronodules on chest X-ray).
Kaposi sarcoma	Typical gross appearance in skin or oropharynx of persistent, initially flat, patches with a pink or violaceous colour, skin lesions that usually develop into plaques or nodules	Macroscopic appearance at endoscopy or bronchoscopy, or by histology

CLINICAL EVENT	CLINICAL DIAGNOSIS	DEFINITIVE DIAGNOSIS
Cytomegalovirus disease (other than liver, spleen or lymph node)	Retinitis only: may be diagnosed by experienced clinicians. Typical eye lesions on fundoscopic examination: discrete patches of retinal whitening with distinct borders, spreading centrifugally, often following blood vessels, associated with retinal vasculitis, haemorrhage and necrosis	Compatible histology or cytomegalovirus demonstrated in cerebrospinal fluid by culture or DNA (by polymerase chain reaction)
Central nervous system toxoplasmosis	Recent onset of a focal nervous system abnormality consistent with intracranial disease or reduced level of consciousness AND response within 10 days to specific therapy	Positive serum toxoplasma antibody AND (if available) single or multiple intracranial mass lesion on neuroimaging (computed tomography or magnetic resonance imaging)
HIV encephalopathy	Disabling cognitive and/ or motor dysfunction interfering with activities of daily living, progressing over weeks or months in the absence of a concurrent illness or condition other than HIV infection that might explain the findings	Diagnosis of exclusion: and (if available) neuroimaging (computed tomography or magnetic resonance imaging)

CLINICAL EVENT	CLINICAL DIAGNOSIS	DEFINITIVE DIAGNOSIS
Extrapulmonary cryptococcosis (including meningitis)	Meningitis: usually subacute, fever with increasing severe headache, meningism, confusion, behavioural changes that respond to cryptococcal therapy	Isolation of *Cryptococcus neoformans* from extrapulmonary site or positive cryptococcal antigen test on cerebrospinal fluid or blood
Disseminated non-tuberculous mycobacterial infection	No presumptive clinical diagnosis	Diagnosed by finding atypical mycobacterial species from stool, blood, body fluid or other body tissue, excluding the lungs
Progressive multifocal leukoencephalopathy	No presumptive clinical diagnosis	Progressive nervous system disorder (cognitive dysfunction, gait/speech disorder, visual loss, limb weakness and cranial nerve palsies) together with hypodense white matter lesions on neuro-imaging or positive polyomavirus JC polymerase chain reaction on cerebrospinal fluid
Chronic cryptosporidiosis (with diarrhoea lasting more than one month)	No presumptive clinical diagnosis	Cysts identified on modified Ziehl-Nielsen stain microscopic examination of unformed stool
Chronic isosporiasis	No presumptive clinical diagnosis	Identification of *Isospora*
Disseminated mycosis (such as coccidiomycosis, histoplasmosis or penicilliosis)	No presumptive clinical diagnosis	Histology, antigen detection or culture from clinical specimen or blood culture

CLINICAL EVENT	CLINICAL DIAGNOSIS	DEFINITIVE DIAGNOSIS
Recurrent non-typhoid *Salmonella* bacteraemia	No presumptive clinical diagnosis	Blood culture
Lymphoma (cerebral or B-cell non-Hodgkin)	No presumptive clinical diagnosis	Histology of relevant specimen or, for central nervous system tumours, neuroimaging techniques
Invasive cervical carcinoma	No presumptive clinical diagnosis	Histology or cytology
Visceral leishmaniasis	No presumptive clinical diagnosis	Diagnosed by histology (amastigotes visualized) or culture from any appropriate clinical specimen
HIV-associated nephropathy	No presumptive clinical diagnosis	Renal biopsy
HIV-associated cardiomyopathy	No presumptive clinical diagnosis	Cardiomegaly and evidence of poor left ventricular function confirmed by echocardiography

Appendix 3: Modified adverse event form

Medical Event (syndrome or symptom)	Start-stop Date<br><br>Ongoing	Toxicity / Maximum Intensity<br>(1) Mild<br>(2) Moderate<br>(3) Severe<br>(4) Very severe	Action taken<br>(Enter all that apply)<br>(1) No action<br>(2) Drug stopped/ interrupted<br>(3) Drug discontinued<br>(4) Additional treatment given<br>(5) Other-specify	Probable cause of event (enter all that apply)<br>(1) Study Drug Treatment<br>(2) Paradoxical reaction<br>(3) Concomitant medication<br>(4) HIV disease<br>(5) Infectious cases (specify)<br>a) viral; b) bacterial c) fungal d) protozoal<br>e) non-specific<br>(6) Other cause (specify)	Was event serious?<br><br>1=Yes<br>2=No<br>(if event is serious fill in **SAE** form)
Event No._ _ _	Start Date<br>Stop Date<br>Ongoing ☐	Week ☐☐☐☐<br>Severity or Change ☐☐☐☐		AE due to an infection: 1 = yes; 2 = no<br>If 'yes' - fill infection event check list ☐	
Event No._ _ _	Start Date<br>Stop Date<br>Ongoing ☐	Week ☐☐☐☐<br>Severity or Change ☐☐☐☐		AE due to an infection: 1 = yes; 2 = no<br>If 'yes' - fill infection event check list ☐	
Event No._ _ _	Start Date<br>Stop Date<br>Ongoing ☐	Week ☐☐☐☐<br>Severity or Change ☐☐☐☐		AE due to an infection: 1 = yes; 2 = no<br>If 'yes' - fill infection event check list ☐	

Appendix 4: Infection event checklist

Medical Event (syndrome or symptom)<br><br>*Use Event No. recorded on AE form*	Start-stop Date<br><br>Ongoing	Infection diagnosis using case definitions<br><br>(e.g. bronchitis, urinary tract infection)<br><br>*Refer to duration of infection SOP*	Infectious AE case definition:<br>EDL = 1; WHO = 2;<br>Discussed with ID = 3	Type of infection<br><br>1 = viral; 2 = bacterial<br>3 = fungal; 4 = protozoal;<br>5 = non-specific	Was event serious?<br><br>1=Yes<br>2=No<br>(if event is serious fill in **SAE** form)
Event No._ _ _	Start Date ____________<br>Stop Date ________<br>Ongoing ☐				
Event No._ _ _	Start Date ____________<br>Stop Date ________<br>Ongoing ☐				
Event No._ _ _	Start Date ____________<br>Stop Date ________<br>Ongoing ☐				
