# Supplementary material for: Consumption of Sutherlandia frutescens by HIV-Seropositive South African Adults: An Adaptive Double-Blind Randomized Placebo Controlled Trial
Source: PLoS One. 2015 Jul 17;10(7):e0128522. doi: 10.1371/journal.pone.0128522 (PMC4506018; doi:10.1371/journal.pone.0128522)
Supplement: S2 Table — (DOCX) [file pone.0128522.s005.docx]

**S2 Table**: Baseline and Week 24 electrocardiogram intervals in Phase 2 Placebo (N = 34) and *S. frutescens* 1,200 mg (N = 36) arms

|  |  | Baseline | | Week 24 | |  |
| --- | --- | --- | --- | --- | --- | --- |
|  |  | Placebo | *S. frutescens* | Placebo | *S. frutescens* | P-value^*^ |
| PR interval, median (IQR) msec |  | 148,0 (133,3; 171,3) | 156,0 (145,3; 169,3) | 152,0 (138,0; 160,0) | 150,0 (143,3; 166,0) | 0,91 |
| QRS duration, median (IQR) msec |  | 84,0 (77,3; 86,7) | 84,0 (77,7; 88,0) | 82,0 (77,3; 87,3) | 82,0 (76,0; 90,0) | 0,98 |
| Corrected QT, interval (median (IQR) msec |  | 408,0 (401,0; 421,7) | 402,0 (395,7; 415,0) | 405,0 (395,3; 418,0) | 404,0 (399,0; 411,3) | 0,85 |

^*^P-value for comparison between Week 24 data
