# Supplementary material for: Consumption of Sutherlandia frutescens by HIV-Seropositive South African Adults: An Adaptive Double-Blind Randomized Placebo Controlled Trial
Source: PLoS One. 2015 Jul 17;10(7):e0128522. doi: 10.1371/journal.pone.0128522 (PMC4506018; doi:10.1371/journal.pone.0128522)
Supplement: S3 Table — (DOCX) [file pone.0128522.s006.docx]

**S3 Table**: Comparison of adverse events by system in the Stage 2 analysis *S. frutescens* 1,200 mg and placebo

| System | *S. frutescens* (N = 39) | Placebo (N = 38) | Total |
| --- | --- | --- | --- |
| Increased appetite | 6 | 5 | 11 |
| Respiratory | 39 | 26 | 65 |
| Gastrointestinal / hepatic | 35 | 37 | 72 |
| Genitourinary | 37 | 26 | 63 |
| Dermatological | 12 | 14 | 26 |
| Lymphatic / haematological | 5 | 5 | 10 |
| Cardiovascular | 3 | 4 | 7 |
| Ear / nose / throat | 52 | 34 | 86 |
| Renal / electrolyte | 37 | 25 | 62 |
| Headache | 13 | 7 | 20 |
| Nervous system | 5 | 4 | 9 |
| Fatigue | 2 | 5 | 7 |
| Elevated creatine kinase | 2 | 1 | 3 |
| Pregnancy | 1 | 0 | 1 |
| Musculoskeletal | 14 | 12 | 26 |
| Weight loss | 0 | 4 | 4 |
| Biochemical metabolic | 1 | 2 | 3 |
| Ophthalmic | 4 | 2 | 6 |
| Night sweats / constitutional | 2 | 0 | 2 |
| Total | 270 | 213 | 483 |
