# Supplementary material for: Consumption of Sutherlandia frutescens by HIV-Seropositive South African Adults: An Adaptive Double-Blind Randomized Placebo Controlled Trial
Source: PLoS One. 2015 Jul 17;10(7):e0128522. doi: 10.1371/journal.pone.0128522 (PMC4506018; doi:10.1371/journal.pone.0128522)
Supplement: S4 Table — (DOCX) [file pone.0128522.s007.docx]

**S4 Table**: Weight and body fat over time in the combined Stage 1 and Stage 2 analysis *S. frutescens* 1,200 mg (N = 54) and placebo (N = 53)

| Fat Measure | Baseline | | Week 12 | Week 24 | | P-value |  |  |
| --- | --- | --- | --- | --- | --- | --- | --- | --- |
|  | Mean (SD) | | Mean (SD) | Mean (SD) | |  |  |  |
| Weight Kg  *S. frutescens* | 71.8 (12.2) | | 72.8 (11.9) | 72.5 (11.89) | | 0.32 |  |  |
| Placebo | 76.6 (15.5) | | 77.2 (15.5) | 76.9 (15.4) | |  |  |  |
| BMI  *S. frutescens* | 28.0 (5.0) | | 28.4 (5.0) | 28.3 (4.9) | | 0.32 |  |  |
| Placebo | 29.3 (6.3) | | 29.5 (6.2) | 29.4 (6.2) | |  |  |  |
| Chest/Triceps Fat  *S. frutescens* | 3.2 (1.3) | | 3.3 (1.1) | 3.2 (1.0) | | 0.48 |  |  |
| Placebo | 3.5 (1.4) | | 3.5 (1.3) | 3.5 (1.2) | |  |  |  |
| Abdominal/Suprailliac Fat  *S. frutescens* | 2.4 (1.0) | | 2.5 (0.9) | 2.4 (0.9) | | 0.98 |  |  |
| Placebo | 2.5 (0.9) | | 2.5 (0.9) | 2.6 (0.9) | |  |  |  |
| Thigh Fat  *S. frutescens* | 5.1 (1.3) | | 5.6 (1.3) | 5.7 (1.2) | | 0.58 |  |  |
| Placebo | 5.3 (1.6) | | 5.5 (1.7) | 5.8 (1.4) | |  |  |  |
|  |  |  | | |  |  |  |  |
