# Supplementary material for: Consumption of Sutherlandia frutescens by HIV-Seropositive South African Adults: An Adaptive Double-Blind Randomized Placebo Controlled Trial
Source: PLoS One. 2015 Jul 17;10(7):e0128522. doi: 10.1371/journal.pone.0128522 (PMC4506018; doi:10.1371/journal.pone.0128522)
Supplement: S5 Table — (DOCX) [file pone.0128522.s008.docx]

**S5 Table**: Quality of life scores in the combined Stage 1 and Stage 2 analysis *S. frutescens* 1,200 mg (N = 54) and placebo (N = 53)

|  | *S. frutescens*  N = 54 | Placebo  N = 53 | P-value |
| --- | --- | --- | --- |
| CES-D Total Mean (SD) |  |  | 0.798 |
| Baseline | 15.5 (8.7) | 13.9 (9.2) |  |
| Week 4 |  |  |  |
| Week 12 | 14.6 (8.8) | 13.8 (8.0) |  |
| Week 24 | 14.7 (9.2) | 14.1 (10.1) |  |
| CES-D > 16 |  |  | 0.829 |
| Baseline | 42.3% | 35.2% |  |
| Week 12 | 43.3% | 34.7% |  |
| Week 24 | 32.7% | 33.3% |  |
| MOS-HIV  Pain |  |  | 0.226* |
| Baseline  Week 4 | 4.7 (0.8)  4.6 (0.9) | 4.5 (1.1)  4.6 (1.0) |  |
| Week 12 | 4.5 (1.0) | 4.4 (0.9) |  |
| Week 24 | 4.7 (0.9) | 4.3 (1.0) |  |
| Vitality |  |  | 0.145* |
| Baseline  Week 4 | 2.7 (0.4)  2.7 (0.4) | 2.7 (0.5)  2.6 (0.5) |  |
| Week 12 | 2.7 (0.5) | 2.6 (0.5) |  |
| Week 24 | 2.7 (0.4) | 2.6 (0.5) |  |
| Role |  |  | 0.602* |
| Baseline  Week 4 | 2.0 (0.2)  1.9 (0.2) | 1.9 (0.3)  1.9 (0.2) |  |
| Week 12 | 1.9 (0.2) | 1.9 (0.3) |  |
| Week 24 | 1.9 (0.2) | 2.0 (0.2) |  |
| Social |  |  | 0.006 |
| Baseline  Week 4 | 5.4 (1.3)  5.0 (1.5) | 4.5 (1.9)  5.2 (1.4) | (Interaction) |
| Week 12 | 5.1 (1.4) | 5.2 (1.5) |  |
| Week 24 | 5.1 (1.5) | 5.1 (1.4) |  |
| Mental Health |  |  | 0.031 |
| Baseline  Week 4 | 4.6 (0.9)  4.4 (1.2) | 4.1 (1.5)  4.6 (1.2) | (Interaction) |
| Week 12 | 4.2 (1.3) | 4.4 (1.3) |  |
| Week 24. | 4.5 (1.0) | 4.6 (1.1) |  |
| Energy |  |  | 0.404* |
| Baseline  Week 4 | 3.9 (1.2)  4.0 (1.1) | 4.3 (1.4)  4.1 (1.3) |  |
| Week 12 | 4.2 (1.1) | 4.1 (1.2) |  |
| Week 24 | 4.1 (1.2) | 4.2(1.2) |  |
| Health Distress |  |  | 0.956* |
| Baseline  Week 4 | 4.6 (0.9)  4.6 (1.0) | 4.4 (1.2)  4.8 (1.0) | 0.055 # |
| Week 12 | 4.7 (1.0) | 4.7 (1.1) |  |
| Week 24 | 4.7 (1.1) | 4.8 (1.1) |  |
| Cognitive Function |  |  | 0.705* |
| Baseline  Week 4 | 4.4 (1.0)  4.3 (1.1) | 4.4 (1.4)  4.5 (1.1) |  |
| Week 12 | 4.4 (1.2) | 4.5 (1.1) |  |
| Week 24 | 4.5 (1.0) | 4.6 (1.0) |  |
| General Health Perception |  |  | 0.759* |
| Baseline  Week 4 | 3.3 (1.0)  3.5 (0.9) | 3.5 (0.9)  3.6 (1.0) |  |
| Week 12 | 3.5 (1.0) | 3.4 (0.9) |  |
| Week 24 | 3.6 (1.0) | 3.6 (0.9) |  |
| QOL |  |  | 0.933* |
| Baseline  Week 4 | 3.9 (0.9)  4.1 (0.9) | 3.9 (1.0)  4.0 (1.0) | 0.030# |
| Week 12 | 4.1 (0.9) | 3.9 (1.0) |  |
| Week 24 | 4.1 (1.0) | 4.3 (0.7) |  |
| Health Compared to Others |  |  | 0.150* |
| Baseline  Week 4 | 4.3 (0.9)  4.4 (0.9) | 4.4 (0.6)  4.7 (0.5) | 0.001# |
| Week 12 | 4.5 (0.8) | 4.5 (0.7) |  |
| Week 24 | 4.6 (0.7) | 4.8 (0.4) |  |
| PSS |  |  |  |
| Baseline | 24.9 (5.9) | 24.7 (6.0) | 0.893* |
| Week 4 | 23.6 (6.2) | 23.5 (6.0) |  |
| Week 12 | 23.2 (4.8) | 23.1 (6.7) |  |
| Week 24 | 23.5 (6.2) | 22.3 (6.1) |  |

*Main Effect of Group (Irrespective of Time)

# Main Effect over Time (Irrespective of Group)

Abbreviations: CES-D (Center for Epidemiologic Studies Depression Scale), MOS-HIV (Medical Outcomes Study HIV Health Survey), QOL (Quality of Life), PSS (Perceived Stress Scale).
