# Supplementary material for: Consumption of Sutherlandia frutescens by HIV-Seropositive South African Adults: An Adaptive Double-Blind Randomized Placebo Controlled Trial
Source: PLoS One. 2015 Jul 17;10(7):e0128522. doi: 10.1371/journal.pone.0128522 (PMC4506018; doi:10.1371/journal.pone.0128522)
Supplement: S1 Protocol — (PDF) [file pone.0128522.s009.pdf]

Protocol

**Protocol Title:**	A Randomized, Double-blind, Placebo-controlled Study of the Safety and Efficacy of *Lessertia frutescens (L.) Goldblatt and J.C. Manning (syn. Sutherlandia frutescens (L.) R. Br.)* in HIV-infected South Africans
**Protocol Number:**	TICIPS002_RP01
**Protocol Date Revision:**	October 7, 2009
**Study Phase:**	II
**Project Leader:**	D Wilson, MBChB, FCP(SA)
**Protocol Author(s):**	J A Syce, PhD<br>D Wilson, MBChB, FCP(SA)<br>K Goggin, PhD
**Investigator:**	Dr D Wilson<br>Department of Medicine, Edendale Hospital,<br>Private Bag X 509, Plessislaer 3216<br>PIETERMARITZBURG<br>SOUTH AFRICA.
**Sponsor:**	**National Center for Complementary and Alternative Medicine**<br>**National Institutes of Health (http://nccam.nih.gov)**
**Implemented by:**	**TICIPS** (The International Centre for Indigenous Phytotherapy Studies)<br>c/o The South African Herbal Science and Medicine Institute (SAHSMI)<br>University of Western Cape (UWC), Private Bag X17, BELLVILLE, SOUTH AFRICA

Confidentiality Agreement

This document is a confidential communication of TICIPS. Acceptance of this document constitutes the agreement by the recipient that no unpublished information contained within will be published or disclosed without prior written approval, except that this document may be disclosed to the appropriate Ethics Committee and Regulatory Authority under the condition that they are requested to keep it confidential.

TABLE OF CONTENTS

		PAGE
**LIST OF ABBREVIATIONS AND DEFINITIONS**		**6**
**STUDY CONTACT LIST**		**7**
**PROTOCOL SYNOPSIS**		**9**
**FLOWCHART**		**13**
**1**	**INTRODUCTION**	**14**
1.1	Background	14
1.2	Study Rationale	16
**2**	**STUDY OBJECTIVES**	**17**
2.1	General Aim	17
2.2	Primary Objective(s)	17
2.3	Secondary and Tertiary Objective(s)	17
**3**	**STUDY POPULATION**	**17**
3.1	Number of Subjects Planned	17
3.2	Inclusion Criteria	17
3.3	Exclusion Criteria	18
3.4	Recruitment and Attrition	18
3.5	Criteria for Discontinuation	19
**4**	**STUDY ENDPOINTS**	**19**
4.1	SAFETY ENDPOINTS	19
4.1.1	Primary Safety Endpoints	19
4.1.2	Secondary and Tertiary Safety Endpoints	20
4.2	Efficacy Endpoints	20
4.2.1	Potential Mediators	20
4.2.2	Moderating Variables	20
4.2.3	HIV Disease Progression Measures	20
**5.**	**INVESTIGATIONAL PLAN**	**20**
5.1	Study Design	20
5.2	Scheduled Clinic Visits	21

5.3 Visits and Assessments 22
5.4 Measurements at each visit 23
5.4.1 Visit 0: Screening Visit 23
5.4.2 Baseline Visit 1: Day 1 – Randomization & 1st Drug Issue 23
5.4.3 Visit 2: Treatment and Assessment (Week 2) 23
5.4.4 Visit 3: Treatment and Assessment (Week 4) 24
5.4.5 Visit 4: Treatment and Assessment (Week 8) 24
5.4.6 Visit 5: Treatment and Assessment (Week 12) 24
5.4.7 Visit 6 & 7: Treatment and Assessment (Week 16 and Week 20) 25
5.4.8 Visit 8: Treatment and Assessment (Week 24) 25
5.5 Specific Detail on Measurements 25
5.5.1 Adverse Events 25
5.5.2 Safety Reporting 26
5.5.3 12-Lead ECG 26
5.5.4 Laboratory Investigations 26
5.5.5 Mediator and Moderator Variables 27
5.5.6 HIV Disease Progression Measure 29
5.6 Strategies to Maximize Adherence to Study Procedures 29
5.7 Criteria for Discontinuation 30
5.8 Assessment of Study Medication Adherence 30

6 INVESTIGATIONAL PRODUCT 30
6.1 Investigational Products and Treatments 30
6.2 Treatment Schedule 30
6.2.1 Randomization 30
6.2.2 Study Product Dosing 30
6.2.3 Study Duration 30
6.3 Identity of Study Products 30
6.4 Storage and Accountability 31
6.5 Allowed Medication 31
6.6 Compliance 31

7 STATISTICAL METHODS 32
7.1 Statistical Design / Model 32
7.2 General 32
7.3 Planned Analysis 34

8 ETHICS 35
8.1 Ethics Review 35
8.2 Ethical Conduct of the Study 35

8.3	Subject Information and Consent	35
8.4	Subject Data Protection	35
8.5	Insurance and Indemnity	35
**9**	**HUMAN SUBJECTS**	**35**
9.1	Risks to Participants	35
9.1.1	Human Participants Involvement and Characteristics	35
9.1.2.	Sources of Materials	36
9.1.3	Potential Risks	36
9.2	Adequacy of Protection Against Risks	36
9.2.1	Recruitment and Informed Consent	36
9.2.2	Protection Against Risk	36
9.3	Potential Benefits of the Proposed Research to the Participants	37
9.4	Importance of the Knowledge to be Gained	37
9.5	Collaborating Sites	37
9.6	Women and Minority Inclusion in Clinical Research	37
9.6.1	Inclusion of Women	37
9.6.2	Inclusion of Children	37
9.7	Data and Safety Monitoring Plan	38
9.8	Vertebrate Animals	38
9.9	Minorities and Women	38
**10**	**STUDY DOCUMENTATION AND ARCHIVING OF DATA**	**38**
10.1	Data Collection Form Completion and Submission	38
10.2	Data Management	38
10.3	Record, Storage and Archive	39
**11**	**DATA QUALITY ASSURANCE**	**39**
**12**	**STUDY TIME TABLE AND TERMINATION**	**39**
**13**	**REFERENCES**	**40**
**14**	**APPENDICES**	**44**
14.1	Declaration of Helsinki	
14.2	Clinical Trial Compensation Guidelines	
14.3	Sample Patient Information Leaflet and Informed Consent Form	
14.4	Pill Count for Adherence Assessment	
14.5	Product Information A (http://www.sutherlandia.org/index.html)	

14.6 Product Information B (Background: www.sutherlandia.org/chemistry.html)

14.7 Product Information C (Supplier)

14.8 Product Information D (Package Insert Equivalent)

14.9 Product Information E (Label)

14.10 Study Questionnaires and Measurement Instruments

LIST OF ABBREVIATIONS AND DEFINITIONS

The following abbreviations and specialist terms are used in this study protocol:

**Abbreviation**	**Explanation**
AE	Adverse Event
ALP	Alkaline phosphatase
ALT	Alanine aminotransferase
b.i.d	*bis in die* = twice daily
CD4+	count of CD4 expressing helper T lymphocytes
CD8+	count of CD8 expressing cytotoxic T lymphocytes
CES-D	The Center for Epidemiologic Studies Depression Scale
CK	creatine phosphokinase
CNS	Central Nervous System
CRF	Case Report Form
ECG	Electrocardiogram
GABA	Gamma aminobutyric acid
GCP	Good Clinical Practice
GIT	Gastro-Intestinal Tract
Hb	Haemoglobin
Hct	Haematocrit
HDL-C	High Density Lipoprotein –cholesterol
HPLC	High performance liquid chromatography
ICH	International Conference of Harmonization
IEC	Independent Ethics Committee
IRB	Independent Review Board
LCMS	Liquid chromatography linked mass spectrometry
LDL-C	Low Density Lipoprotein -cholesterol
MS	Mass spectrometry
MCH	Mean corpuscular haemoglobin
MCHC	Mean corpuscular haemoglobin concentration
MCV	Mean corpuscular volume
ml	milliliter
MRC	Medical Research Council
OHRP	Office of Human Research Protections (of Federal Department of Health & Human Services, USA)
PPS	Perceived Stress Scale
RDW	Red cell distribution width
RBC	Red Blood Cells
SAE	Serious Adverse Event
SAHSMI	South African Herbal Science and Medicine Institute
SG	Specific gravity
SEM	Standard error of the mean
SLE	Systemic lupus erythematosus
TICIPS	The International Center for Indigenous Phytotherapy Studies
TSH	Thyroid-stimulating hormone
UWC	University of the Western Cape
UCT	University of Cape Town
WBC	White blood cells

Definitions

Herbal Medicines: Mainly whole, fragmented or cut plants, part of plants in an unprocessed state, usually in dried form, but sometimes fresh. Herbal drugs are precisely defined by the botanical scientific name according to the binomial system (genus, species, variety).

African traditional medicine: The total body of knowledge and techniques for the preparation and use of substances, measures and practices that are based on the socio-cultural and religious bedrock of African communities, are founded on personal experience and observations handed down from generation to generation, either verbally or in writing, and are used for the diagnosis, prevention or elimination of imbalances in physical, mental or social well-being.

STUDY CONTACT LIST

PROJECT TEAM:

Project Leader (SA): Douglas Wilson, MBChB, FCP(SA)
E-mail: wilsond1@ukzn.ac.za
Telephone: +27 (0) 33 395 4911
Mobile: +27 (0) 827867698
Telefax: +27 (0) 33 3954060

Project Co-Leader (US): Kathy Goggin, PhD
E-mail: goggink@umkc.edu
Telephone: +1 (816) 235 1059

Clinical Core Leader (SA): James A Syce, M Pharm, PhD
E-mail: jsyce@uwc.ac.za
Telephone: +27 21 959 2192
Mobile: 082 202 3315
Telefax: +27 21 959 1324

Consultant : HIV Physician and Pharmacologist (SA): Gary Maartens, FCP (SA), MMed
E-mail: gary.maartens@uct.ac.za
Telephone: +27 (0) 21 406 6286

Consultant: Clinical Trials Expert (US): Mary Gerkovich, PhD
E-mail: gerkovichm@umkc.edu
Telephone: +1 (816) 235 6480

Consultant: Quality of Life Expert (US): Albert W Wu, MD, MPH
E-mail: awu@jhsph.edu
Telephone: +1 410 955 6567

Biostatistician (SA): Danelle Kotze, PhD
E-mail: dkotze@uwc.ac.za
Telephone: +27 21 959 3203
Mobile: +27 (0) 83 648 8886
Telefax: +27 21 959 1356

Biostatistician (US): Karen Williams, PhD
E-mail: williamsk@umkc.edu
Telephone: +1 (816) 235 2058

STUDY SITE PERSONNEL TEAM:

Principal Investigator: Douglas Wilson, MBChB, FCP(SA)

Co- Investigator: Dr Bongani Thembela, MBChB, FCP(SA)
Chief Specialist, Department of Medicine
Pietermaritzburg Hospitals Complex
E-mail: thembelabl@telkomsa.net
Mobile: +27 (0) 84351875

Study Co-ordinator: Patricia Bartman, Professional Nurse
E-mail: pat.bartman@gmail.com
Mobile: +27 (0) 723749407

Physical address: Department of Medicine
Edendale Hospital
Pietermaritzburg , South Africa

<u>For reporting a serious adverse event, please contact:</u>

Medical Advisor: Dr Halima Daewood, MBChB, FCP(SA)
Principal Specialist, Infectious Diseases
Grey's Hospital, Pietermaritzburg
E-mail: halimadaewood@gmail.com
Mobile: +27 (0) 826535786

Data and Safety Monitoring Board (DSMB) / Chairperson: Dr Mark Blockman
E-mail: mblockmn@uctgsh1.uct.ac.za
Telephone: +27 21 21 406 6496
Mobile: +27 (0) 83 458 5510

<u>For questions regarding data quality assurance, please contact:</u>

Independent Study Monitor: Dinmari Wilsenach
Clinical Research Associate, On Q Consulting
E-mail: dinmariw@onqsa.co.za
Mobile: 82 77 23 555

PROTOCOL SYNOPSIS

**TITLE**	A Randomized, Double-blind, Placebo-controlled Study of the Safety and Efficacy of *Lessertia frutescens (L.) Goldblatt and J.C. Manning (syn. Sutherlandia frutescens (L.) R. Br.)* in HIV-infected South African adults.		
**Investigational site:**	Department of Medicine, Edendale Hospital, Pietermaritzburg, South Africa		
**Investigators:**	Dr Douglas Wilson, Dr. K Goggin		
**Sponsor:**	National Center for Complementary and Alternative Medicine (NCCAM), National Institutes of Health (NIH)<br>The International Centre for Indigenous Phytotherapy Studies (TICIPS)		
**Representatives:**	Prof. Quinton Johnson, Prof. William Folk		
**Study number:**	TICIPS002_RP01		
**Final Protocol:**	Nov 2007	**Clean File:**	**2008 / 2009**
**Ethics Approval:**	Oct 2007 / Dec 2007	**Statistical analysis:**	**2008 / 2009**
**Regulatory Approval**	Feb 2007	**Study Report:**	**2008 / 2009**
**Clinical Phase:**	II	**Timelines:**	**Jan 2010 / Dec 2010**
**OBJECTIVES:**	Primary Objectives:<br>• To determine the safety of *Lessertia frutescens* when used by HIV-1 infected adults with early disease.<br>• To document the impact of *Lessertia frutescens* on the number, nature and duration of secondary infections in HIV-infected adults with early disease<br><br>Secondary and Tertiary Objectives:<br>• To determine the effect of *Lessertia frutescens* on quality of life indices in HIV-infected adults measured by the Medical Outcomes Study HIV Health Survey (MOS-HIV) (secondary outcome) and length duration of secondary infections (tertiary outcome.)<br>• To document the impact of *Lessertia frutescens* on markers of HIV disease progression		
**STUDY DESIGN:**	The study is a 2-stage, double-blind, randomized, placebo-controlled study following a two-stage, statistical selection theory design of Steinberg and Venzon[74]. Up to forty-eight study subjects will be randomized (with substitution) into Stage 1 that will comprise a 4-arm parallel group (one placebo and 3 treatment groups) trial. One or possibly two interim analyses will be performed to determine continuation to Stage 2. A blinded interim analysis to determine the superior active treatment arm of Stage 1 to be continued in Stage 2, will be conducted after 8 subjects per arm have completed the 24-week 4-arm dosing regimen. If there are no clear differences between any of the treatment arms and the placebo arm at this point, the study will continue with Stage 1 until 12 subjects per group have completed the 24-week dosing regimen and the blinded interim analysis is repeated. The study will be terminated if the interim analysis identifies either significant safety issues or demonstrable futility, (Sutherlandia groups associated		

with significantly more adverse events than placebo). If treatment arms are equivalent, the 1200 mg bid dose will be selected for continuation to Stage 2. Following determination of treatment arm continuation in the blinded interim analysis, the accrual and randomization of study subjects to the selected active and placebo control arms will continue, and study kept double-blind, until a total of 48 subjects per treatment arm are completed.

**RANDOMISATION**	**STAGE 1**<br>**4-arm randomisation**				**STAGE 2**<br>**2-arm randomisati[illegible]**	
**Week 1 / Baseline**	**Wk 24**	**INTERIM ANALYSIS**	**Wk 24**	**INTERIM ANALYSIS**	**Wk 24**	**TO[illegible]**
Placebo	8	S: Continue Stage 2<br>NS: Continue n=12	12	S: Continue Stage 2<br>NS: Study Termination	36	48
400 mg bid<br>L frutescens	8		12		36	48
800 mg bid<br>L frutescens	8		12			
1200 mg bid<br>L frutescens	8		12			
Note:<br>S = Significant<br>NS = Non Significant		TOTAL	48	TOTAL	72	12[illegible]

<u>* Randomization with substitution will be performed to achieve a final sample of 48 per stage 2 groups.</u>

SUBJECTS:

<u>Inclusion Criteria</u>:

1. Age ≥21 years and <65 years
2. HIV-1 infection documented in the medical record by two different rapid tests for HIV-1 antibodies
3. CD4 count ≥350 cells/µL
4. Viral load ≥1,000 copies/mL
5. Normal haematological function (haemoglobin >10.0 g/dL, absolute neutrophil count >1.5x10^9, eosinophil count <1.0x10^9, platelet count >100x10^{11})
6. Absence of clinically significant renal disease: 1. serum creatinine <140 µmol/L; 2. glomerular filtration rate ≥ 60 mL/min calculated using the formula of Cockroft and Gault and 3. absence of haematuria and/or ≥1+ proteinuria on urine dipstick.
7. Normal liver function (INR <1.5, bilirubin <1.5x normal, ALT <2x normal, ALP <2x normal)
8. Random glucose <11.1 mmol/L
9. Normal electrocardiogram
10. Regular attendance at the Wellness Clinic for at least 4 visits
11. Cognitive capacity sufficient to provide informed consent
12. Has not taken traditional medication for 28 days prior to screening

<u>Exclusion criteria</u>:

	1. Any AIDS-defining diagnosis<br>2. Weight loss >5% of body weight within the preceding six months<br>3. Other features of undiagnosed tuberculosis (including cough, fatigue, drenching night sweats and abnormal chest radiograph).<br>4. Any other significant disease (for example active tuberculosis, hypertension, diabetes mellitus and other endocrine disorders, peptic ulcer disease, gastrointestinal malabsorption, psychiatric illness) either newly diagnosed or controlled by medication.<br>5. Use of any allopathic medication other than isoniazid for tuberculosis prophylaxis<br>6. Use of traditional medicines within the past 28 days<br>7. Prior or current use of antiretroviral therapy<br>8. History of allergic conditions (e.g. asthma, eczema, urticaria requiring medical therapy on more than one occasion) or drug allergy/hypersensitivity.<br>9. Either history or family history of autoimmune disease (e.g. systemic lupus erythmatosis, Guillian Barre, haemolytic anaemia)<br>10. Alcohol use of >7 units per week or 3 units per occasion, tobacco use of more than 10 cigarettes per day or description of recreational drug use within the past 6 months<br>11. Pregnancy or breast-feeding<br>12. Women of childbearing potential who are sexually active and not using medically accepted dual contraceptive measures, as judged by the investigator.<br>13. Participation in a clinical study of any investigational product 1 month prior to the screening visit.
**PRODUCT TO BE EVALUATED**	**Test Product:** <u>*Lessertia frutescens*</u> 400mg capsules:<br>1 i.e. 400mg BID;<br>2 i.e. 800mg BID and<br>3 i.e. 1200mg BID<br><br>**Reference Product:** Placebo capsules
**DURATION OF STUDY**	28 weeks ± 30 days
**ENDPOINTS:**	<u>**SAFETY**</u><br>1. Primary, secondary and tertiary endpoints are defined as weight loss and change in CD4 cell count, change in Quality of Life measures measured by the Medical Outcomes Study HIV Health Survey (MOS-HIV), and length of infections (e.g., common cold, sore throat, URTI, UTI, stye in eye), respectively.<br>2. Adverse events and serious adverse events. Given the findings of the earlier Phase I trial, the following list represents potential AEs that might be observed. This is not, however, an exhaustive list and any adverse event that patients report will be tracked and documented.<br>a. Cardiovascular (e.g., palpitations, nose bleeds)<br>b. CNS (e.g., headaches, nervousness, insomnia,

	dizziness)<br>c. GIT (e.g., diarrhea, gastritis, constipation, stomach cramps, nausea)<br>d. Infections (e.g., common cold, sore throat, URTI, UTI, stye in eye)<br>e. Allergy (e.g., dermatitis)<br>f. Appetite (e.g., increase, decrease)<br>g. General (e.g., iliac pain)<br>h. Malaise<br>3. Other markers of HIV progression or phytotherapy-induced effects, as assessed by:<br>a. Vital signs: blood pressure, heart rate, respiratory rate, oral temperature.<br>b. ECG<br>c. Urinalysis: leucocytes, nitrate, urobilinogen, protein, pH, blood, specific gravity, ketones, bilirubin and glucose<br>d. Blood hematology and chemistry<br>Haematology: RBC, MCHC, MCH, MCV, RDW, Hct, Hb, WBC Differential count –lymphocytes monocytes, neutrophils, eosinophils, basophils; CD4+,<br>Biochemistry: Serum - Bilirubin (total and conjugated) AST, ALT, ALP, GGT, LDH, CK, Protein, albumin, globulin, cholesterol (total, LDL-C, HDL-C), glucose, urea, creatinine, sodium, potassium, chloride, calcium, magnesium.<br>e. Viral load<br>f. Active constituent levels: Canavanine, Pinitol and SU1.<br><br>**LENGTH OF INFECTION**<br>The following primary infection event end points will be measured:<br>1. Number of secondary infection events defined by clinical case definitions and / or by infectious disease consultant opinion<br>2. Category of secondary infection events (viral, bacterial, fungal, protozoal, non-specific)<br>3. Duration of secondary infection events determined by date of onset (start date) and date of recovery (stop date)
**SAMPLE SIZE**	120 subjects
**STATISTICAL ANALYSIS**:	Stage I: Study will use a two stage, statistical selection theory design of Steinberg and Venzon[74]. Interim analysis will be done once 32 subjects (8 per stage 1 treatment) have completed 24 weeks. To select the dose arm that, compared to placebo, is maximally tolerated and effective the proportion of failures in terms of the outcomes: weight loss, change in CD4 cell count, QoL scores and length of infection, for each treatment group will be compared with that of the placebo group. A gap of at least 2 subjects categorized as failures (criteria for determination for failure outlined in section 7.2) between active treatment group and placebo is required to select active treatment for Phase II. If this minimal difference in failure does not exist, analysis will be repeated when 12 subjects per group have completed 24 weeks treatment. Study will be terminated if the interim analysis identified either significant safety issues or demonstrated futility, defined as treatment arms being worse than placebo. If treatment arms are equivalent, the 1200 mg bid dose will be

	selected for continuation to Stage 2. Following determination of treatment arm continuation for Stage 2, the accrual and randomization of a further 36 study subjects to the selected active and placebo control arms will continue, and study kept double blind. Assuming there will be an attrition rate of 15%, we will utilize experimental randomization with replacement until there are 48 subjects completed in the placebo and active treatment arms.<br><br>Stage II: Appropriate descriptive analyses will be performed to examine distributional characteristics for collected measures, and to summarize changes over time as a function of group assignment. During the initial phase of the statistical analyses, bivariate relationships among primary and secondary outcome measures and variables thought to impact the outcomes (Potential Mediators and Background/Moderators) will be explored. In addition, appropriate analyses (Chi-Square, Mann-Whitney and Independent T-test) for variables measured at the nominal, ordinal and interval/ratio level, respectively, will be conducted to determine whether the randomly assigned groups are equivalent at the start of the study with respect to the demographic and other measures collected at baseline. The results of these analyses will determine what additional variables will be incorporated in the subsequent hypothesis testing of the effect of treatment arm across time on the primary and secondary outcomes (e.g., linear regression, mixed modeling, repeated-measures ANOVA, or analysis of covariance, as appropriate). As described in the research design section, outcome measures will also be dichotomized according to specific definitions of "failure", and failure rates compared between groups. Additional exploratory analyses will be conducted to examine significant predictors of failure for these outcomes. A series of logistic regression models will be fitted to obtain odds ratios (OR+ 95% CI) for failure if indicated by bivariate analyses.<br><br>Double data entry will be performed to ensure data integrity and identifiers removed to ensure subject confidentiality. Statistical analyses of primary and secondary outcomes will be conducted using the de-identified dataset, and appropriate post hoc analyses performed as necessary.

FLOWCHART

**Week**	**-1** **Screening**	**0** **Baseline**	**2**	**4**	**8**	**12**	**16**	**20**	**24**	**28** **Follow up, if applicable**
**Visit**	**0**	**1**	**2**	**3**	**4**	**5**	**6**	**7**	**8**	**9**
Informed consent	X									
Medical/ smoking history	X									
Inclusion/Exclusion criteria	X	X								
Vital signs: heart rate, blood pressure, respiratory rate, oral temperature and weight	X	X	X	X	X	X	X	X	X	X
Subcutaneous fat measurement (biceps, triceps, iliac crest)		X	X	X	X	X	X	X	X	
Height	X									
Review of Medical Symptoms	X	X	X	X	X	X	X	X	X	
Physical examination	X					X			X	X
12-lead ECG	X					X			X	X**

*Pregnancy test	X	X		X	X	X	X	X	X	
Allocation of subject number		X								
Laboratory tests: Haematology & Biochemistry and Urinalysis	X			X	X	X			X	X**
ANF Serum Test		X				X			X	
Concomitant medication	X	X	X	X	X	X	X	X	X	X
Quality of Life		X		X		X			X	
CD4 Cell Count	X					X			X	
HIV RNA Viral Load	X					X			X	
Storage of urine sample		X				X			X	
Storage of blood sample		X				X			X	
Potential Mediators										
Depressive Symptoms		X		X		X			X	
Stress		X		X		X			X	
Background/Moderating Variables										
Beliefs in the Benefits of Sutherlandia		X							X	
Medical History	X									
Demographics	X									
Alcohol / Substance Use	X									
SA – Alcohol Substance Inventory		X		X		X			X	
African Cultural Related Symptoms		X		X		X			X	
Patient Satisfaction with Study									X	
Adverse events			X	X	X	X	X	X	X	X
Study medication - Dispensed/Returned (D/R)		D	R/ D	R/ D	R/ D	R/ D	R/ D	R/ D	R	
Micronutrient Supplement Dispensed (28 day supply)		X		X						
Drug adherence – Pill counts			X	X	X	X	X	X	X	
Unannounced Phone Pill Count				X	X					

Note:
*Females of childbearing age
**If applicable

1 INTRODUCTION

1.1 Background

An estimated 4.69 million South Africans are infected with Type 1 human immunodeficiency virus (HIV).[1] HIV infection is incurable and, if untreated, is ultimately fatal in the approximately 95% of infected individuals. HIV has profound effects on the immune system, causing specific depletion of CD4 T-lymphocytes resulting in immune dysregulation followed by increasingly severe immune suppression manifesting as the acquired immunodeficiency syndrome (AIDS).[2,3]Receiving a positive HIV test result has equally profound effects on an individual's quality of life and mood. These impacts are important as observed higher levels of depression and anxiety has been shown to adversely affect the course of HIV disease.[4,5] Combinations of antiretroviral therapy (ART) are used to effectively suppress viral replication and restore immune function and quality of life in patients with significant immune suppression or symptomatic HIV disease.[6] However, these drugs are expensive, associated with a significant incidence of adverse side effects, and with the development of drug resistant virus.[7] There is an urgent need for therapies for use in early HIV disease that modulate the progression of HIV infection and defer the need for ART.

L. frutescens is thought to be among the most efficacious plants used in southern African traditional medicine. The plant has been used in indigenous settings to treat serious microbial infections as well as cancer and inflammatory conditions. Beneficial effects have also been observed in wasting conditions and stress-related

disorders.[8,9] Tinctures, infusions and decoctions of the leaves and stems of *L. frutescens* have been widely used in the Cape Region of South Africa since the value of this herbal medicine was first discovered by the Khoi, San and Nama people. The traditional Tswana name for the plant, 'Phetola', means 'it changes'. Literally translated this means that the plant changes the course of many illnesses into favourable outcomes. Furthermore, the North Sotho name, 'Lerumo-lamadi', means the 'spear for the blood' which refers to the value of *L. frutescens* as a powerful blood-purifier that acts as an all-purpose tonic.[10]

The medicinal value of *L. frutescens* has been ascribed to a variety of its constituents including pinitol, γ-amino butyric acid (GABA), and L-canavanine. Pinitol is claimed to have anti-inflammatory effects and may have clinical application in treating wasting due to AIDS.[9,11] GABA is an inhibitory neurotransmitter that may have beneficial effects on stress, anxiety and depression. Canavanine is a potent L-arginine antagonist with anti-infective and anti-inflammatory effects.[12] Anti-cancer effects for this substance have also been documented.[13] Concerns have been raised over possible induction by canavanine of auto-immune diseases (such as systemic lupus erythematosis). However, this effect is thought only to occur at very high doses or in predisposed individuals or in the presence of low arginine levels.[14] Recently, *Sutherlandia fructescens* extract has been shown to possess antioxidant properties in a cell-free system that may partially explain the plant's anti-inflammatory effects.[15]

A recently completed study on vervet monkeys (*Chlorocebus aethiops*) conducted by the South African Medical Research Council, found that up to nine times the recommended dose of *L. frutescens* (81 mg/kg body weight per day for 3 months) resulted in no significant changes to relevant haematological, biochemical and physiological parameters. It was concluded that *L. frutescens* is non-toxic in vervet monkeys.[16] Results of a Phase I double blind placebo-controlled study conducted by The International Center for Indigenous Phytotherapy Studies (TICIPS) through the South African Herbal Science and Medicine Institute (SAHSMI) at the University of the Western Cape to evaluate the safety of *L. frutescens* in 25 healthy adult volunteers have just been made available. This randomized double blind placebo controlled study randomized12 healthy subjects to the treatment arm where they received 400mg *Lessertia frutescens* leaf power capsules twice a day and 13 subjects to the control arm where they received an identical placebo capsule twice per day. Participants were seen at monthly visits throughout the 3-month study period. The subjects provided blood and urine samples for haematology/biochemistry (i.e., RBC, WBC, serum analysis, serum proteins) and urinanalysis (i.e., SG, pH, protein, glucose, ketone, bilirubin, blood, & urobilinogen), completed a 12-lead ECG, and reported any adverse events at each visit. Results indicate that the 800mg per day dose was well tolerated by study participants and resulted in no significant changes to relevant haematological, biochemical or physiological parameters. However, there was a notable difference in the length of infections (i.e., common cold, sore throat, URTI, UTI) with the treatment group experiencing fewer days with symptoms ($M = 4.3$, $SD = 2.2$) as compared to the control group ($M = 8.0$, $SD = 5.9$). This finding has important implications for clinical management of early HIV disease. The formulation of *L. frutescens* used in these studies is of a reproducible quality and is the only form of *L. frutescens* that has been clinically tested.

The course of HIV infection is primarily determined by the rate of HIV replication assessed using the HIV viral load, which measures the number of viral RNA copies per millilitre of plasma.[17] However, recent evidence suggests that HIV RNA has low predictive validity for individual CD4 cell decline.[76] The impact of HIV on the immune system is quantified by measuring the CD4 T-lymphocyte count. As HIV disease progresses the CD4 count falls and the risk of developing AIDS-defining opportunistic infections and malignancies increases. A CD4 count of <200 cells/µL is considered indicative of severe immune suppression.

The effect of HIV infection on the immune system's cytokine networks is complex and incompletely understood. Cytokine plasma levels, peripheral blood monocyte cell cytokine secretion after antigen stimulation and cytokine receptor expression are all important variables. However, certain principles are becoming apparent.[18] HIV replication is inhibited by the interferon family of cytokines (α, β and γ) and by granulocyte-macrophage colony stimulating factor, interleukin-10, interleukin-13, interleukin-16 and β-chemokines. The pro-inflammatory cytokines tumour necrosis factor α and β, interleukin-1, interleukin-6 and macrophage-colony stimulating factor stimulate HIV replication. Interleukin-4 is considered bi-directional, having both stimulatory and inhibitory properties.[19] Wasting due to HIV disease is mediated by tumour necrosis factor alpha (TNF-α) and interleukin-6.[20]

Elevated TNF-α levels are thought to contribute to HIV-induced neurodegeneration, initially manifesting as impaired cognition and reduced mental health.[21] Psychological disorders such as stress and depression are

thought to adversely affect immune function, possibly by influencing cytokine networks, and pro-inflammatory cytokines may adversely affect mood and cognition.[22]

Other parameters such as hemoglobin concentration have been shown to affect quality of life in HIV-infected adults and anemia is known to be an independent risk factor for disease progression and mortality.[71] In the absence of anemia due to opportunistic infections or drug side-effects the most common cause for anemia in HIV infection is anemia of chronic disorders, probably mediated by the cytokine-induced hepatic acute phase protein hepcidin.[72]

Highly active antiretroviral therapy has profoundly altered the prognosis of HIV infection. However, the benefits of antiretroviral therapy are limited by the concern of long-term drug toxicities and the development of drug resistant virus. The optimum time to initiate antiretroviral therapy is controversial, needing to balance risk of opportunistic infections and irreversible immunological damage with that of toxicity and resistance. Antiretroviral therapy is also costly, and requires complex healthcare infrastructure, dedicated patient adherence, and costly clinical/laboratory monitoring. Furthermore an effective vaccine for HIV infection is probably many years away[25] and the incidence of new infection remains high in many parts of sub-Saharan Africa.

Traditional phytotherapies may have beneficial effects on early HIV disease by slowing the rate of disease progression and allowing antiretroviral therapy to be safely deferred for clinically meaningful periods of time. In particular, it is possible that *L. frutescens* may have useful effects in HIV infected adults. Early HIV infection has been associated with a significant reduction in quality of life in multiracial South Africans measured by the Medical Outcomes Study HIV Health Survey (MOS-HIV) instrument.[23] The observed anti-inflammatory properties of *L. frutescens* may favorably influence cytokine networks with beneficial effects on quality of life, viral replication and immune functioning. It is thus essential to document that this widely used phytotherapy can be used safely in HIV-infected adults, and to attempt to demonstrate potentially beneficial effects for outcomes that patient's likely value (e.g., quality of life). Moreover, recent data has demonstrated that there are significant in vitro interactions between Sutherlandia and commonly used antiretrovirals.[24] Healthcare providers in Africa thus also need data to inform their HIV-infected patients' use of traditional medicine.

In summary, *L. frutescens* has a long history of use in many southern African communities. It is most commonly believed to influence stress, anxiety, low-mood and wasting, all of which may significantly impact on quality of life and therefore indirectly, on progression of diseases such as HIV infection. Indeed, the herb is used commonly by HIV-infected individuals and there are a number of anecdotal case reports suggesting that benefit exist. This proposal is designed to further investigate the safety and effectiveness of this product in accordance with internationally accepted conventions for the evaluation of traditional or indigenous medicines that historically have been used to treat human ailments. Given the findings from the only known controlled studies of *L. frutescens* and the stated purpose of most users of this substance, this proof of principle study will focus on investigating the safety of *L. frutescens* in a sample of adult HIV-1 infected South Africans with early disease and the impact of *L. frutescens* on markers of HIV disease progression. Secondarily the study will focus on the effect of *L. frutescens* on quality of life indices (an outcome that patients will likely be most interested in). Tertiarily, the outcome of duration of common secondary infections (an outcome that clinicians will likely be most interested in) will be explored along with markers of HIV disease progression (i.e., CD4 cell count, viral load).

1.2 Study Rationale

An estimated 4.69 million South Africans are infected with Type 1 human immunodeficiency virus (HIV). HIV infection is incurable and, if untreated, is ultimately fatal in approximately 95% of infected individuals. *L. frutescens* is thought to be among the most efficacious plants used in southern African traditional medicine. The plant has been used in indigenous settings to treat serious microbial infections, especially HIV infection, as well as cancer and inflammatory conditions. Beneficial effects have also been observed in wasting conditions and stress-related disorders. It is possible that *L. frutescens* may have useful effects in HIV infected adults. Early HIV infection has been associated with a significant reduction in quality of life in multiracial South Africans measured by the Medical Outcomes Study HIV Health Survey (MOS-HIV) instrument.[23] The observed anti-inflammatory properties of *L. frutescens* may favourably influence cytokine networks with beneficial effects on quality of life, viral replication and immune function. It is essential to document that this widely used phytotherapy can be used safely in HIV-infected adults, and to attempt to demonstrate potentially beneficial effects.

One factor that often complicates studies on traditional plant medicine is the inconsistency of preparations from one batch to another. In this study a commercially available capsule form of *S. frutescens* (viz. African Ginseng^{TM}) which contains standardized leaf powder of the *S. frutescens* subsp. *Microphylla* Elite PN1^{TM} chemotype will be used. As part of the study the content uniformity of the preparation will be validated before it is used in the study so that the problem of inconsistent dosage should be removed as a possible complicating factor. In addition, the presence of select chemical markers in the plasma of subjects who had taken the treatment will be monitored as further indicator of the consistency of the test products to be used.

Many herbal medicines such as S*utherlandia* are increasingly being commercially exploited and advocated for use still based only on unproven claims of efficacy and safety arising from poorly monitored (sometimes even dubious) African traditional medicine use practices. To derive optimal benefit from herbal medicines both their preparation and use (whether in traditional setting and/or along with non-traditional medicine) should be thoroughly evaluated and standardized. However, this is not receiving adequate attention in South Africa at present. Thus the development of added capacity and expertise to do such scientific and clinical evaluation of traditionally used medicines through the South African Herbal Science and Medicine Institute at UWC and in South Africa would also be a most worthy endeavor (and an additional objective with this study and mission of TICIPS). To facilitate such development collaboration with other sites will be encouraged. Thus the study will be conducted in KwaZulu-Natal where the availability of the necessary infrastructure, expertise, other ongoing HIV research projects and extensive traditional Sutherlandia use and traditional healer interest exist.

Given the widespread indigenous use of *S. frutescens* as a phytotherapeutic, and its non-toxic effects shown in the pre-clinical studies in a primate and a recently completed Phase I safety study, it is logical to now assess its safety and efficacy, in a Phase II clinical study in adults with early HIV infection.

Finally, in both the traditional and commercial settings, the "normal" recommended dose of *Sutherlandia* is 600 to 800 mg daily. It is however also known that in the traditional setting doses substantially higher than this is frequently used and /or the *Sutherlandia* combined with other plant medicines. Results of the completed Phase 1 study showed that 800mg (ca. 12mg/kg for average 66 kg human) daily doses of Sutherlandia leaf powder produced no serious adverse effects while doses as high as 3x and 9x (i.e. 81mg/kg body weight) the equivalent human dose also produced no adverse effects in the vervet monkey. To optimize the chances of clearly establishing whether *Sutherlandia* is safe and efficacious or not in the traditional use setting a dose escalating study with doses higher than the currently recommended doses used in commercial products would thus be well justified.

Given all the above, this multi-component study will consequently firstly, evaluate the clinical safety of *Lessertia frutescens* as well as assess its impact on objectively determined quality of life parameters including depression and stress, length of common infections, markers of HIV disease progression and cytokine profile. In addition, in the initial phase of this two stage design, the optimally tolerated and effective dose of *L. frutescens* out of 3 options, the highest being 3x the usual dose recommended for the commercial preparations, will be determined. Thereafter, (stage 2) sufficient participants will be enrolled to ensure the truly effective evaluation of the selected dose, i.e. in a randomized double-blind placebo controlled trial with sufficient power. Finally, TICIPS has assembled a highly experienced team of researchers to determine the safety and efficacy of *L. frutescens, a* multi-disciplinary team that has experience in conducting and analyzing HIV-related clinical and pharmacological studies in South Africa and in evaluating the influence of psychological factors on quality of life and a successful study should be ensured.

1.3 Summary of the findings from stage 1 of this study

Screening and Enrolment:

One hundred and seventeen patients were screened for Stage I and 56 (47.9%) were enrolled from April 2008 to March 2009. Common reasons for exclusion included low CD4 T-lymphocyte count, active tuberculosis, hematuria or proteinuria, current use of traditional medicines, and in early stages of recruitment, some participants were excluded due to low HIV viral load. The viral load inclusion criterion was reduced from ≥20,000 copies / mL to ≥10,000 copies / mL in May 2008 and to ≥1,000 copies /mL in October 2008 as many potential subjects were being excluded without good justification, and it became apparent that initial recruitment targets were not being met.

Baseline demographics, randomization and early terminations:

Fifty women (89%) and six men (11%) were enrolled with a mean age of 31.1 years (standard deviation 7.3 years). Fifteen participants were enrolled onto the placebo arm, thirteen participants onto the 400 mg arm, thirteen participants onto the 800 mg arm, and fifteen participants onto the 1,200 mg arm. Five participants were terminated early from the study: one for pregnancy; one for withdrawal of consent; one for incomplete week 24 visit; one for impaired renal function that was present at baseline; and one participant due to the expenses associated with visiting the study site.

The gender imbalance we experienced in our recruited cohort (89% women) mirrors clinical realities and is typical of healthcare-seeking behavior in southern African HIV-positive adults. Specifically, men are more likely to seek medical attention when at a more clinically advanced stage of the infection than women and less likely to volunteer for studies that require them to be away from work. We know of no evidence to suggest that Sutherlandia affects men differently than women.

Complete data was included in the interim analysis from fourteen participants in the placebo arm, and twelve participants from each of the 400 mg, 800 mg and 1,200 mg arms.

Adverse event summary:

Two hundred adverse events were recorded up to 01 August 2009. Adverse events were graded using the NIH Division of AIDS (DAIDS) definitions. No grade 4 adverse events were recorded. One participant became pregnant and exited early from the study (assessed as a grade 3 adverse event).

One hundred and forty one events (70.5%) were grade 1 toxicity and 59 events (29.5%) grade 2 toxicity. The commonest reported adverse events were: increased appetite; and gastrointestinal, respiratory, genitourinary, lymphatic / hematological, and dermatological conditions. The commonest lymphatic / hematological disorder was cervical and axillary lymphadenopathy attributable to HIV infection. No adverse events were attributed to the study product / placebo only, and no study events were typical of an adverse drug reaction.

One participant in the placebo group (titre 1:160) and one participant in the 400 mg group (titre 1: 80) converted from ANF negative to ANF positive. These low level titers are not clinically significant. No episodes of clinical vasculitis were reported.

Evaluation for product toxicity

There were no clinically significant changes detected in hepatic, renal or haematological parameters. No clinically significant changes were detected in serial electrocardiograms.

End point analysis:

The purpose of the interim analysis was 1) to ensure that there was no evidence of harm to participants and 2) to provide information about which dose/arm to use to complete the trial. The summary of results below reveals that the placebo group did not differ from any of the study drug arms on Weight Loss failures and unexpectedly evidenced fewer CD4 failures than any of the treatment arms. Accordingly, neither of these End Points will be used to inform selection of treatment arm to continue with into Stage 2.

The failure rate in the placebo group for Days Infection and Quality of Life-Energy Sub-scale were worse by a gap of 2 than the 1200mg dose group. These differences met the criteria for selection of the active treatment arm for Stage 2. The 1200mg dose group and 400mg dose group did not differ by 2 on Quality of Life-Energy Sub-scale, but given that the 1200mg dose group was superior to both the 400mg dose group and placebo on Days Infection and Quality of Life-Energy Sub-scale and is the highest tolerable dose, we propose its selection as the active treatment arm for Stage 2.

Summary report of interim statistical analysis:

Weight: Failure rates in the placebo group (1/14) did not differ from active groups (0/12; 0/12; 0/12).

CD4 Change:* Failure rates for 400mg (4/12), 800mg (8/12) and 1200 mg (4/12) groups were worse than placebo (1/14).

*P= 0.012 Pearson's Chi-squared test for combined Sutherlandia groups vs. placebo

Note: Mean change in CD4 percentage of the total lymphocyte count remained stable across all four groups and the failure rate for CD4 change was assessed as not being clinically significant by both the investigators and the DSMB.

Quality of Life subscales:

Energy: Failure rate for Energy in the placebo group (3/14) was worse by at least 2 when compared to the 400mg (0/12) or 1200mg (1/12) group.

Mental: Placebo failure rates not different (by 2) from any of the active groups.

Physical: Placebo group (1/14) did not differ from 800mg (2/12) and 1200mg (1/12) groups, but did better than 400mg group (3/12).

Distress: Placebo group (0/14) had a better failure rate than any of the Active groups. Specifically, 400mg (2/12) and 800mg (4/12) group failure rates were greater than placebo by 2, but 1200mg (1/12) group was not.

Days Infection: 1200mg (3/12) group failure rates is less than placebo (6/14) and 400mg (7/12) by at least 2.

Viral Load: While viral load was not a primary outcome variable for the interim analyses, these data were explored to provide a better understanding of the CD4 data. The placebo, 400mg, and 1200mg groups showed mean reductions in viral load. The 800mg group evidenced an average increase. The pattern is useful information, but of limited clinical relevance, so we also explored ≥ .5 Log Reduction in VL.

VL Log Change: Again, the placebo (5/14), 400mg (4/12) and 1200mg (5/12) evidenced marked Log reductions in VL. The 800mg group did not (1/12).

Comment:

In this interim analysis consumption of Sutherlandia appears safe in this cohort of HIV-infected adults, with no evidence of product-related toxicities. Hematological, liver enzyme and electrolyte measurements did not show meaningful changes over the study period in any group. No SAEs attributed to Sutherlandia consumption have occurred, and the pattern of AEs does not indicate that participants are being harmed by taking part in the study.

The investigators note the reduction in viral load and mean gain in CD4 cells in three of the four arms. This is thought to be attributable to the non-specific benefits of participating in structured clinical care. During discussion with the NIH/NCCAM the CD4 failure rate was not thought of be clinically meaningful as this was based on a single reading using a test that normally shows a variance of up to 15%. Importantly the mean CD4% remained steady in all arms between baseline and week 24.

Clinically meaningful conclusions regarding the safety or indications of efficacy of Sutherlandia cannot be made using the Stage 1 data only (n = 55). We note that the design of the trial is such that the purpose of the interim analysis was to provide information about which dose/arm to use to complete the trial, and was not powered to make definitive statements regarding safety or efficacy. Given that this is the first placebo-controlled, randomized clinical trial of an African phytotherapy in an HIV-infected cohort, the lack of otherwise reliable safety data, the widespread use of Sutherlandia and other traditional medicines, the partnership of traditional healers and allopathic medical scientists and interest that this trial has generated in subcontinent, there is great need for a convincing statement about the trial and its findings. For all of these reasons, the investigators recommend completion of Stage 2, randomizing participants to either Sutherlandia 1200 mg twice daily or placebo twice daily, in accordance with the goals described in the study protocol.

2. STUDY OBJECTIVES

2.1 General Aim

- To determine the Safety and Efficacy of *Lessertia frutescens (L.) Goldblatt and J.C. Manning (syn. Sutherlandia frutescens (L.) R. Br.)* in HIV-infected South Africans

2.2 Primary Objective(s)

- To determine the Safety of *Lessertia frutescens* when used by HIV-1 infected adults with early disease.
- To document the impact of *Lessertia frutescens* on markers of HIV disease progression
- To assess the impact of *Lessertia frutescens* on number, category (viral, bacterial, fungal, non-specific) and duration of infection events

2.3 Secondary/Tertiary Objective(s)

- To determine the effect of *Lessertia frutescens* on quality of life in HIV-infected adults [a secondary outcome and measured by the Medical Outcomes Study HIV Health Survey (MOS-HIV)] and length of infection (a tertiary outcome).
  Hypothesis 2: Higher levels of energy assessed using the Medical Outcomes Study HIV Health Survey (MOS-HIV) after six months treatment with *Lessertia frutescens* will be demonstrated.

3 STUDY POPULATION

3.1 Number of Subjects Planned

HIV infected adults with early HIV disease (≥350 CD4 cells/µL and HIV viral load > 1,000 copies/mL) will be recruited from the Wellness Clinics attached to Edendale Hospital, Kwazulu Natal to enroll 120 patients. To be enrolled, the following criteria have to be fulfilled at both the Screening (Visit 0) and Randomisation (Visit 1):

:

3.2 Inclusion Criteria

1. Age ≥21 years and <65 years
2. HIV-1 infection documented in the medical record by two different rapid tests for HIV-1 antibodies
3. CD4 count ≥350 cells/µL
4. Viral load ≥1,000 copies/mL
5. Normal haematological function (haemoglobin >10.0 g/dL, absolute neutrophil count >$1.5x10^9$, eosinophil count <$1.0x10^9$, platelet count >$100x10^{11}$)
6. Absence of clinically significant renal disease: a). serum creatinine <140 µmol/L; b). glomerular filtration rate ≥ 60 mL/min calculated using the formula of Cockroft and Gault;* and c). absence of haematuria and/or ≥1+ proteinuria on urine dipstick.
7. Normal liver function (INR <1.5, bilirubin <1.5x normal, ALT <2x normal, ALP <2x normal)
8. Random glucose <11.1 mmol/L
9. Normal electrocardiogram.**
10. Regular attendance at the Wellness Clinic for at least 4 visits
11. Cognitive capacity sufficient to provide informed consent
12. Has not taken any traditional medication for 28 days prior to screening

3.3 Exclusion Criteria

1. Any AIDS-defining diagnosis
2. Weight loss >5% of body weight within the preceding six months
3. Other features of undiagnosed tuberculosis (including cough, fatigue, drenching night sweats and abnormal chest radiograph).
4. Any other significant disease (e.g. active tuberculosis, hypertension, diabetes mellitus and other endocrine disorders, peptic ulcer disease, gastrointestinal malabsorption, psychiatric illness) either newly diagnosed or controlled by medication.
5. Use of any allopathic medication other than isoniazid for tuberculosis prophylaxis.
6. Use of traditional medicines within the past 28 days.
7. Prior or current use of antiretroviral therapy
8. History of allergic conditions (e.g. asthma, eczema, urticaria requiring medical therapy on more than one occasion) or drug allergy/hypersensitivity.

9. Either history or family history of autoimmune disease (e.g. systemic lupus erythmatosis, Guillian Barre, haemolytic anaemia)
10. Alcohol use of >7 units per week or 3 units per occasion, tobacco use greater than 10 cigarettes per day or description of recreational drug use within the past 6 months
11. Pregnancy or breast-feeding
12. Women of childbearing potential who are sexually active and not using medically accepted dual contraceptive measures, as judged by the investigator***
13. Participation in a clinical study of any investigational product 1 month prior to the screening visit

Notes: *Formula of Cockroft and Gault: glomerular filtration rate = (140 – age) x weight (kg) / 0.82 x plasma creatinine (µmol/L). In women plasma creatinine is multiplied by 0.85 instead of 0.82)
**Acceptable variants: first degree heart block with normal QRS duration and normal axis; QTc≤0.44 sec; bradycardia and/or voltage criteria for left ventricular hypertrophy in precordial leads in physically fit individuals with normal cardiac examination.
***Dual contraceptive measures: consistent use of the male condom with use of another recognized contraceptive method (female condom, intramuscular medroxyprogesterone or norethisterone, combined oral contraceptive pill, intrauterine device). Complete abstinence from sexual intercourse and the stated intention to continue abstinence will be accepted as the alternative to dual contraception.

3.4 Recruitment and Attrition

This single site study will be conducted in Kwazulu Natal, and participants will be recruited from the Wellness Clinics associated with the Pietermaritzburg Hospital Complex and Edendale Hospital.

The Pietermaritzburg Hospital Complex serves over 1 million adults, with 10-15% of the population estimated to be infected with HIV. Currently, there are over 6000 patients registered with the HIV Clinics within the Complex, and with the advent of the national antiretroviral rollout programme and readily available access to voluntary counseling and testing for HIV infection this number is expected to increase substantially. The Complex has a network of well-integrated Communicable Diseases Clinics that provide opportunistic infection prophylaxis for HIV-infected adults and, more recently, antiretroviral therapy (ART) as part of the South African Government's rollout programme. Only patients with CD4 count of <200 cells/µL or an AIDS-defining diagnosis qualify for government-funded ART. This has led to the establishment of Wellness Clinics that provide counseling, support groups and tuberculosis (TB) prophylaxis for the estimated 90% of HIV-infected adults who do not currently qualify for ART. The participants enrolled onto this study will not be taking antiretroviral therapy and will not be eligible for this treatment as their baseline CD4 count will be ≥350 cells/µL, and they will not have developed an AIDS-defining diagnosis. Many HIV-infected South Africans will therefore have elected to take alternative treatments, including traditionally used medicines, in an attempt to support their immune systems and positively influence the rate of HIV disease progression. Preparations containing *L. frutescens* are widely promoted for this purpose, and are readily available throughout South Africa.

Edendale Hospital is the largest hospital in the Complex and serves a predominantly peri-urban and rural population. The HIV Clinic provides both District and Regional level care, and the Wellness Clinic coordinator is based within the hospital. The hospital is situated in the largest peri-urban community around Pietermaritzburg, and is situated in close proximity to several community clinics. Edendale Hospital also provides training for medical students and residents in internal medicine from the University of KwaZulu-Natal.

Consistent with other studies' that have been led in South Africa and the United States, detailed contact information will be obtained from participants to ensure proper follow-up. In addition to providing personal contact information (i.e., phone, address, cell phone, e-mail, and work phone and address), participants will be asked to provide contact information for at least two friends or family members who always know how to contact them. At each visit, contact information will be updated and participants will be reminded of the importance of completing all study visits. In addition, participants will receive reimbursement of expenses of R150 ($25.00) at each of the study visits and an additional R150 ($25.00) for each of the quality of life evaluation visits to defray travel costs and help ensure continued participation. Further, participants will be informed of the importance of the study and their involvement at the enrollment visit. These techniques have been successfully used in other longitudinal studies to enhance participants' level of commitment and motivation.

3.5 Criteria for Discontinuation

Subjects may be discontinued from study treatment and assessments at any time, at the discretion of the investigator. Specific reasons for discontinuing a subject from the study are:

1. Withdrawal of informed consent.
2. Development of exclusion criteria, pregnancy or other safety reasons during the study.
3. Protocol non-compliance.
4. Incorrect enrolment or randomization of the subject.

For subjects withdrawn from the study, the same measurements and assessments should be performed as done at Visit 8. Adverse events should be followed up and study medication returned by the subjects.

4 STUDY ENDPOINTS

4.1 PRIMARY ENDPOINTS

4.1.1 Primary Safety Endpoints

The following primary safety endpoints will be measured at each study visit from screening to week 24 (Adverse events will be measured at all study visits following start of study medication):

1. Vital parameters (pulse rate, blood pressure, respiratory rate, oral temperature, weight; height will be recorded at the baseline visit only)
2. Concomitant medications (with start and stop dates)
3. HIV related medical symptoms
4. Adverse events and serious adverse events with start and stop dates. Given the findings of the earlier Phase I clinical study, the following list represents potential AEs that might be observed. This is not; however, an exhaustive list and any adverse event that patients report will be tracked and documented:
    a. Cardiovascular (e.g., palpitations, nose bleeds)
    b. CNS (e.g., headaches, nervousness, insomnia, dizziness)
    c. GIT (e.g., diarrhea, gastritis, constipation, stomach cramps, nausea)
    d. Infections (e.g., common cold, sore throat, URTI, UTI, stye in eye)
    e. Allergy (e.g., dermatitis)
    f. Appetite (e.g., increase, decrease)
    g. General (e.g., iliac pain)
    h. Malaise
    i. Other
5. Urine β-HCG pregnancy test (except week 2)

The following primary safety will be measured at screening, week 4, week 8, week 12 and week 24:

1. Full blood count and differential count
2. Serum urea, creatinine, albumin, electrolytes (sodium, potassium, calcium, inorganic phosphate), ALT, ALP, total bilirubin (with conjugated bilirubin if total bilirubin elevated)
3. Serum cholesterol and triglyceride
4. Non-fasting plasma glucose
5. Urine analysis (Multistix^{R})

The following additional safety endpoints will be measured at Visit 0, 5 and 8 (screening, week 12 and week 24):

1. Physical examination
2. 12-lead electrocardiogram
3. CD4 and Viral load

The following additional safety endpoints will be measured at Visit 1, 5 and 8 (Baseline, week 12 and week 24):

1. Serum and urine stored for analysis of Sutherlandia biomarkers.
2. Antinuclear antibody

4.1.2 Incidence and length of infection events

The following primary infection event end points will be measured:

4. Number of secondary infection events defined by clinical case definitions and / or by infectious disease consultant opinion

5. Category of secondary infection events (viral, bacterial, fungal, protozoal, non-specific)
6. Duration of secondary infection events determined by date of onset (start date) and date of recovery (stop date)

4.1.3 Secondary and Tertiary Safety Endpoints

The following secondary endpoint will be measured at baseline, week 4, week 12, and week 24:

1. Medical Outcomes Study HIV Health Survey (MOS-HIV) [quality of life in HIV-infected adults].

4.2 PRELIMINARY EFFICACY ASSESSMENT

The following efficacy endpoints will be observed at designated study visits as indicated to obtain potential effect size estimates for :

4.2.1 Potential Mediators (At baseline, week 4, week 12 and week 24):

1. Depressive Symptoms (The Center for Epidemiologic Studies Depression Scale (CES-D)
2. Stress (The Perceived Stress Scale (PSS)

4.2.2 Moderating Variables:

1. Beliefs in the benefit of *Sutherlandia* (Baseline and week 24)
2. Medical history (Screening only)
3. Medical symptoms (Each study visit)
4. Demographics (Screening only)
5. Alcohol/substance use (Screening, baseline, week 4, week 12, week 24)
6. African Cultural Related Symptoms (Baseline, week 4, week 12, week 24)
7. Patient Satisfaction with Study (Week 24)

4.2.3 HIV Disease Progression Measures:

1. Weight loss greater than 5% of total body weight (Each study visit)
2. CD4 cell count (Screening, week 12 and week 24)
3. HIV RNA Viral load (Screening, week 12 and week 24)

5 INVESTIGATIONAL PLAN

5.1 Study Design

The study is a 2-stage, double-blind, randomized, placebo-controlled study following a two-stage, statistical selection theory design of Steinberg and Venzon[74]. Up to forty-eight study subjects will be randomized (with substitution) into Stage 1 that will comprise a 4-arm parallel group (one placebo and 3 treatment groups) trial. One or possibly two interim analyses will be performed to determine continuation to Stage 2. A blinded interim analysis to determine the superior active treatment arm of Stage 1 to be continued in Stage 2, will be conducted after 8 subjects per arm have completed the 24-week 4-arm dosing regimen. If there are no clear differences between any of the treatment arms and the placebo arm at this point, the study will continue with Stage 1 until 12 subjects per group have completed the 24-week dosing regimen and the interim analysis is repeated. The study will be terminated if the interim analysis identifies either significant safety issues or demonstrable futility defined as treatment arms being worse than placebo. If treatment arms are equivalent, the 1200 mg bid dose will be selected for continuation to Stage 2. Following determination of treatment arm continuation for Stage 2, the accrual and randomization of study subjects to the active and placebo control arms will continue, and study kept double-blind, until a total of 48 subjects per treatment arm are completed. A schematic- and visit plan is given below.

Informed Consent ⇓ → **Screening -7 days** ---------→ **Baseline Week 0** (⇓ **Randomisation**) ------→ **Week 2, 4, 8** → **Week 12** → **Week 16, 20** → **Week 24** → **30-days Follow-up**

**RANDOMISATION**	**STAGE 1**<br>4-arm randomisation				**STAGE 2**<br>2-arm randomisation	
**Week 1 / Baseline**	**Week 24**	**INTERIM ANALYSIS**	**Week 24**	**INTERIM ANALYSIS**	**Week 24**	**TOTAL**
Placebo	8	S: Continue Stage 2<br>NS: Continue n=12	12	S: Continue Stage 2 NS/Safety: Study Termination	36	48
400 mg bid L frutescens	8		12		36	48
800 mg bid L frutescens	8		12			
1200 mg bid L frutescens	8		12			
Note:<br>S = Significant<br>NS = Non Significant		TOTAL	48	TOTAL	72	120*

* Randomization with substitution will be performed to achieve a final sample of 48 per Stage 2 group.

5.2 Scheduled Clinic Visits

**Visit number**	**Type of visit**	**Day number (& days between visits)**
Visit 0<br>Screening	Information, Consent and Screening	Day -7
Visit1<br>Baseline	Randomization & 1st Drug issue visit	Day 0 (7 ± 2 days after Visit 0)
Visit 2<br>Week 2	After 2 weeks treatment	Day 14 (14 ± 2 days after Visit 1)
Visit 3<br>Week 4	After 4 weeks treatment	Day 28 (28 ± 2 days after Visit 1)
Visit 4<br>Week 8	After 8 weeks treatment	Day 56 (56 ± 3 days after Visit 1)
Visit 5<br>Week 12	After 12 weeks treatment	Day 84 (84 ± 3 days after Visit 1)
Visit 6<br>Week 16	After 16 weeks treatment	Day 112 (112 ± 5 days after Visit 1)
Visit 7<br>Week 20	After 20 weeks treatment	Day 140 (140 ± 5 days after Visit 1)
Visit 8<br>Week 24	After 24 weeks treatment	Day 168 (168 ± 5 days after Visit 1)
Visit 9<br>Follow up, if applicable	30-day follow up, if applicable	Day x (30 ± 3 days after last Visit)

5.3 Visits and Assessments

Study subjects will be assessed for adverse events potentially attributable to *L. frutescens* at screening, baseline, week 2, week 4, week 8, week 12, week 16, week 20, week 24 and 4 weeks after the last dose, if applicable. All participants will receive a micronutrient supplement (Centrum one tablet daily) for the first eight weeks of the study.

**Week**	**-1 Screening**	**0 Baseline**	**2**	**4**	**8**	**12**	**16**	**20**	**24**	**28 Follow up, if applicable**
**Visit**	**0**	**1**	**2**	**3**	**4**	**5**	**6**	**7**	**8**	**9**
Informed consent	X									
Medical/ smoking history	X									
Inclusion/Exclusion criteria	X	X								
Vital signs: heart rate, blood pressure, respiratory rate, oral temperature and weight	X	X	X	X	X	X	X	X	X	X
Subcutaneous fat measurement (biceps, triceps, iliac crest)		X	X	X	X	X	X	X	X	
Height	X									
Review of Medical Symptoms	X	X	X	X	X	X	X	X	X	
Physical examination	X					X			X	X
12-lead ECG	X					X			X	X**
*Pregnancy test	X	X		X	X	X	X	X	X	
Allocation of subject number		X								
Laboratory tests: Haematology & Biochemistry and Urinalysis	X			X	X	X			X	X**
ANF Serum Test		X				X			X	
Concomitant medication	X	X	X	X	X	X	X	X	X	X
Quality of Life		X		X		X			X	
CD4 Cell Count	X					X			X	
HIV RNA Viral Load	X					X			X	
Storage of urine sample		X				X			X	
Storage of blood sample		X				X			X	
Potential Mediators										
Depressive Symptoms		X		X		X			X	
Stress		X		X		X			X	
Background/Moderating Variables										
Beliefs in the Benefits of Sutherlandia		X							X	
Medical History	X									
Demographics	X									
Alcohol / Substance Use	X									
SA – Alcohol Substance Inventory		X		X		X			X	
African Cultural Related Symptoms		X		X		X			X	
Patient Satisfaction with Study									X	
Adverse events			X	X	X	X	X	X	X	X
Study medication - Dispensed/Returned (D/R)		D	R/ D	R/ D	R/ D	R/ D	R/ D	R/ D	R	
Micronutrient Supplement Dispensed (28 day supply)		X		X						
Drug adherence – Pill counts			X	X	X	X	X	X	X	
Unannounced Phone Pill Count				X	X					

Note:
*Females of childbearing age

**If applicable

5.4 Measurements at each visit
See section 5.2 for the visit schedule.

5.4.1 Visit 0: Screening Visit
The subjects will be examined within 14 days prior to the first ingestion of the investigational products to assess their eligibility to participate. Each subject will consent in writing (Appendix 14.3) to the study before the start of the investigational procedures. The informed consent form will also include the study information leaflet.
The examinations and investigations will include:
- Medical history, including history of past use of medications, demographics (date of birth, sex, race), contraception history and alcohol and tobacco consumption patterns.
- Physical examination including assessment of general appearance, cardiovascular, lungs, neurological, mouth, throat and abdomen and measurement of height. The examination will be made in accordance with the normal clinical routines at the clinical study center.
- Vital signs: systolic and diastolic blood pressure in sitting (after 5 minutes rest), pulse, respiratory rate, body weight and oral temperature. Blood pressure will be consistently taken in the same arm.
- ECG: standard 12-lead (see section 5.5.3).
- Review of medical signs and symptoms.
- Review of concomitant medications
- Laboratory tests: Haematology, serum biochemistry and urine analysis (see section 5.5.4).
- Urine β-HCG pregnancy test in non-hysterectomized volunteers. Fertile females must have a negative pregnancy test.
- CD4 Cell Count
- HIV RNA Viral Load.
- Investigator confirms that study subject fulfills inclusion criteria for the study.

5.4.2 Baseline Visit 1: Day 1 - Randomization & 1st Drug Issue
- Study subjects will be seen at the investigational center and investigator will confirm that study subjects fulfill inclusion criteria for the study.
- Vital signs: systolic and diastolic blood pressure in sitting (after 5 minutes rest), pulse, respiratory rate, weight and oral temperature. Blood pressure will be consistently taken in the same arm.
- Assessment of subcutaneous fat.
- Urine β-HCG pregnancy test
- The pre-dose safety assessments and eligibility checks will be recorded on the relevant pages in the individual CRFs (including Urine β-HCG pregnancy test)
- ANF serum test will be performed.
- Assess Quality of Life and potential mediators and background/moderating variables
- Review of medical signs and symptoms.
- Concomitant medication assessment.
- Assign study Participant Identification Number (PIN).
- The randomized subject will be issued with 1 month's supply of study medication, instructed how to take the medication and given next appointment date. Subject should start the first dose at the recommended time (ca: 06:00 – 10:00) on the day of drug issue or start the next day. In addition, they will receive a 1-week emergency supply of study medication to be used if needed before the next study visit. This will be checked at each study visit and re-supplied as needed.
- One month supply of multivitamin supplementation will be issued.
- Storage of serum and urine for analysis for Sutherlandia metabolites (see section 5.5.4)

5.4.3 Visit 2: Treatment and assessment (Week 2)
For this visit the examinations and investigations will include:
- Collection of returned study medication, pill count and re-issue of month's study medication.
- Study medication compliance will be checked and discussed (esp. with respect to incidence and type of reported adverse events).
- Vital signs: systolic and diastolic blood pressure in sitting (after 5 minutes rest) and pulse, respiratory rate, weight and oral temperature. Blood pressure will be consistently taken in the same arm.
- Assessment of subcutaneous fat.

- Review of medical signs and symptoms.
- Adverse event and concomitant medication assessment.

5.4.4 Visit 3: Treatment and assessment (Week 4)

For this visit the examinations and investigations will include:

- Collection of returned study medication and pill count.
- Study medication compliance will be checked and discussed (esp. with respect to incidence and type of reported adverse events).
- Vital signs: systolic and diastolic blood pressure in sitting (after 5 minutes rest) and pulse, respiratory rate, weight and oral temperature. Blood pressure will be consistently taken in the same arm.
- Assess subcutaneous fat.
- Laboratory tests: Haematology, serum biochemistry and urine analysis (see section 5.5.4).
- Urine β-HCG pregnancy test
- Review of medical signs and symptoms.
- Adverse event and concomitant medication assessment.
- Assess Quality of Life and potential mediators and background/moderating variables
- The subject will be issued with 1 month's supply of study medication, instructions on how to take the medication will be reviewed and given next appointment date.
- One month supply of multivitamin supplementation will be issued.
- Between Visit 3 and 4, adherence to study medication will be assessed by unannounced phone contact and pill count.

5.4.5 Visit 4: Treatment and assessment (Week 8)

For this visit the examinations and investigations will include:

- Collection of returned study medication and pill count.
- Study medication compliance will be checked and discussed (esp. with respect to incidence and type of reported adverse events).
- Vital signs: systolic and diastolic blood pressure in sitting (after 5 minutes rest), pulse, respiratory rate, weight and oral temperature. Blood pressure will be consistently taken in the same arm.
- Laboratory tests: Haematology, serum biochemistry and urine analysis (see section 5.5.4).
- Urine β-HCG pregnancy test
- Assessment of subcutaneous fat.
- Review of medical signs and symptoms.
- Adverse event and concomitant medication assessment.
- The subject will be issued with 1 month's supply of study medication, instructions on how to take the medication will be reviewed and given next appointment date.
- Between Visit 4 and 5, adherence to study medication will be assessed by unannounced phone contact and pill count

5.4.6 Visit 5: Treatment and assessment (Week 12)

For this visit the examinations and investigations will include:

- Collection of returned study medication and pill count.
- Study medication compliance will be checked and discussed (esp. with respect to incidence and type of reported adverse events).
- Vital signs: systolic and diastolic blood pressure in sitting (after 5 minutes rest), pulse, respiratory rate, weight and oral temperature. Blood pressure will be consistently taken in the same arm.
- Laboratory tests: Haematology, serum biochemistry and urine analysis (see section 5.5.4).
- ANF Serum Test
- Urine β-HCG pregnancy test
- Assessment of subcutaneous fat.
- Review of medical signs and symptoms.
- Physical examination
- ECG: standard 12-lead (see section 5.5.4).
- Assess Quality of Life and potential mediators and background/moderating variables
- Adverse event and concomitant medication assessment.
- CD4 Cell Count
- HIV RNA Viral Load

- The subject will be issued with 1 month's supply of study medication, instructions on how to take the medication will be reviewed and given next appointment date.
- Storage of serum and urine for analysis for Sutherlandia metabolites (see section 5.5.4)

5.4.7 Visits 6 & 7: Treatment and assessment (Week 16 and Week 20)

For these visits the examinations and investigations will include:

- Collection of returned study medication and pill count.
- Study medication compliance will be checked and discussed (esp. with respect to incidence and type of reported adverse events).
- Vital signs: systolic and diastolic blood pressure in sitting (after 5 minutes rest), pulse, respiratory rate, weight and oral temperature. Blood pressure will be consistently taken in the same arm.
- Urine β-HCG pregnancy test
- Assessment of subcutaneous fat.
- Review of medical signs and symptoms.
- Adverse event and concomitant medication assessment.
- The subject will be issued with 1 month's supply of study medication, instructions on how to take the medication will be reviewed and given next appointment date.

5.4.8 Visits 8: Treatment and assessment (Week 24)

For this visit the examinations and investigations will include:

- Collection of returned study medication and pill count.
- Study medication compliance will be checked and discussed (esp. with respect to incidence and type of reported adverse events).
- Vital signs: systolic and diastolic blood pressure in sitting (after 5 minutes rest), pulse, respiratory rate, weight and oral temperature. Blood pressure will be consistently taken in the same arm.
- Laboratory tests: Haematology, serum biochemistry and urine analysis (see section 5.5.4).
- ANF Serum Test
- Urine β-HCG pregnancy test
- Assessment of subcutaneous fat.
- Review of medical signs and symptoms.
- Physical examination
- ECG: standard 12-lead (see section 5.5.4).
- Assess Quality of Life and potential mediators and background/moderating variables
- Assess Patient Satisfaction with Study.
- Adverse event and concomitant medication assessment.
- CD4 Cell Count
- HIV RNA Viral Load
- Collection of returned dossettes.
- Storage of serum and urine for analysis for Sutherlandia metabolites (see section 5.5.4)

Note: For any subject in which any of the above tests indicated levels outside the normal range or an (s)AE is ongoing, a follow-up visit (visit 9) will be scheduled for an appropriate time (1 month after completion of study participation or withdrawal). Specific tests will be repeated at this visit, as needed.

5.5 Specific detail on measurements

5.5.1 Adverse events (AE)

An adverse event is the development of an undesirable medical condition - e.g. symptoms or abnormal results of an investigation - or the deterioration of a pre-existing medical condition (not relevant to this study). AE's will be collected by means of a standard question: "Have you had any health problems since the previous visit?" AEs will be recorded at every visit. Spontaneously reported AE's and/or observed AE's and the subject's response to this question will be recorded on the AE form with information about seriousness, action taken, date of onset and recovery, maximum intensity and outcome. The subjects will be asked to assess the intensity of the reported AE according to the following scale:

Mild = awareness of sign or symptom, but easily tolerated

Moderate = discomfort sufficient to cause interference with normal activities
Severe = incapacitating, with inability to perform normal activities
Potentially life-threatening = unable to perform basic self-care functions OR medical or operative intervention indicated to prevent permanent impairment, persistent disability or death

A Serious Adverse Event (SAE) is an adverse event occurring during any stage of the study and at any dose of the investigational product or placebo that fulfils one or more of the following criteria:

- Results in death;
- Is immediately life-threatening;
- Requires in-subject hospitalization;
- Results in persistent or significant disability or incapacity (including pregnancy).

The causality of SAEs (i.e. the relationship to study treatment) will be assessed by the investigators, who in completing the relevant Case Report Form must answer 'yes' or 'no' to the question "Do you consider that there is a reasonable possibility that the event may have been caused by the study medication?" The following factors should be considered when deciding if there is a "reasonable possibility" that an AE may have been caused by the investigational product.

- Time course of events and exposure to suspect drug – did the AE occur in a reasonable temporal relationship to the administration of suspect drug?
- De-challenge experience – did the AE resolve or improve on stopping or reducing the dose of the suspect drug?
- Re-challenge experience - did the AE reoccur if the suspected drug was reintroduced after having stopped?
- Laboratory tests – has a specific laboratory investigation confirmed the relationship?
- No alternative cause - the AE cannot be reasonably explained by another aetiology such as an underlying disease (not previously present), other drugs or environmental factors.

There would not be a "reasonable possibility" of causality if none of the above criteria apply or where there is evidence of exposure and a reasonable time course, but any de-challenge is negative or there is another more likely cause of the AE.
In this study the AEs will be noted from the interview at the time of visit.

5.5.2 Safety reporting

All SAEs will be reported to the Sponsor, the South African Medicines Control Council, DSMB, National Center for Complementary and Alternative Medicine, and the Research Ethics Committee within 24 hours of the study team becoming aware of the event. All adverse events will be reported in summary form every six months to the Sponsor and the South African Medicines Control Council. The Data and Safety Monitoring Board affiliated with TICIPS will be asked to undertake an independent review of the adverse events occurring on the study if three or more serious adverse events occur during the course of the study. Unblinding will occur after written notification by the Principal Investigator or the Data and Safety Monitoring Board.

5.5.3 12- Lead ECG

Standard 12-lead ECGs will be performed at screening, week 12, week 24 and only if applicable at post-study follow-up visit (or upon withdrawal). Study subjects will only be randomized if the ECG is normal, or any abnormalities that are noted are considered by the investigator to be not clinically relevant.

5.5.4 Laboratory Investigations

Blood and urine will be collected for clinical pathology tests at the screening visit, weeks 4, 8, 12 and 24, and, if required, at any post-study safety follow-up visits (or upon withdrawal). The following variables will be measured:

- **Haematology:** (10ml blood sample)

Red cells: Erythrocytes, Haemaglobin. Haematocrit, MCV, MCH and MCHC.
White cells: Leucocytes and 5 part differential (neutrophils, lymphocytes. monocytes, eosinophils and basophils); CD4+ and CD8+ counts.
Platelets
CD4 T-lymphocyte count (screening, week 12 and week 24)

- **Clinical Chemistry:** (2 x 10ml blood samples)

Serum analysis: ALP, ALT, cholesterol (total, LDL-C, HDL-C), Total bilirubin (with conjugated bilirubin if total bilirubin is elevated)

Serum electrolytes: Sodium, potassium, calcium, inorganic phosphate, urea, creatinine
Serum proteins: Albumin
Plasma glucose (non-fasting)
Urinalysis (Multistix^R): SG, pH, protein, glucose, ketone, bilirubin, blood and urobilinogen.
Urine β-HCG pregnancy test (all visits except week 2)

- **Virology**

HIV qualitative viral load (screening, Week 12 and week 24)

A local laboratory (Bauer & Partners / Ampath Laboratories) will be used for routine pathology measurements. Urinalysis will be performed at the investigational site. The samples will be processed and shipped as per instructions of the pathology laboratory.

For all laboratory investigations the investigator will receive actual printouts of the data of the subjects. A signed original printout will be attached to the CRF and a copy thereof will be filed in the subject file. If the copy seen by the investigator is the faxed copy, then this is the copy that must be signed and retained. The reference ranges will be provided in the clinical study manual (investigator's file) together with the descriptions of the laboratory methods used. The laboratory will provide normal ranges on each report to ease the investigator's assessment.

As part of the overall safety-monitoring plan, the safety monitor will also assess the data, particularly those falling outside the normal range.

Blood levels and urine levels of active constituent. At baseline and weeks 12 and 24 venous blood samples (10 ml each) will be collected into heparinised tubes for storage for future analysis for the presence of Sutherlandia metabolites. At the 12 week and 24 week visits, subjects will ingest their study medication at the onset of each appointment and blood samples will always be taken at the completion of the study visits (no less than 2 hours post dosing). The blood samples will be collected in tubes labeled with the following information: Study number (TICIPS002_RP1); study day; subject study number and initials; blood sample number (visit 1, 5, 8) and sampling time.

Blood samples will be handled on ice between collection and centrifugation. Within 15 to 30 minutes of collection, the blood samples will be centrifuged at approx. 2600 g for 10 minutes after which the supernatant will be divided into 3 approximate aliquots. The aliquots will be transferred to the appropriately labeled tubes (75 x 12 mm silanised borosilicate test tubes with plastic ripple caps). Similarly a 10 mL sample of urine will be frozen at baseline, weeks 12 and 24 to assess for the presence of Sutherlandia metabolites.

The samples will be stored in a -20 ^{o}C freezer (if for short period and -70 ^{o}C if for longer period) pending shipment for assay (to Pharmaceutics Lab, UWC, or MRC lab in the Western Cape). Samples will be kept frozen during the shipment by packing them in "dry ice".

The plasma samples will be assayed for the targeted plant constituents using high performance liquid chromatography (HPLC) with ultra violet (UV), fluorescence detection and/or mass spectrometric (MS) detection (canavanine) or gas chromagtography (pinitol) in validated procedures developed from the methods of Petritis *et al.* (2000), Shiah *et al.* (2003) and Lein *et al.* (2002), respectively.

5.5.5 Mediator and Moderator Variables: Development of Culturally Appropriate Measures

Measures will be developed especially for this clinical study and also standard measures which have not yet been validated in a Zulu speaking population will be used. Consequently, a pre-trial stage will focus on measurement adaptation and validation. In this stage, the existing measures will be adapted, as necessary; to address domains of interest where established measures are not available or significant alterations of standard measures is required for this clinical study. Next, standard translation/back-translation/forward-translation procedures will be used to adapt all measures for use with the South African population. Finally, all measures will be piloted in a sample of patients similar to those to be recruited for this study and the reliability, validity and acceptability of each one will be assessed. This will be done before onset of the study program.

- **Cultural Adaptation of Measures**

All survey measures (i.e., MOS-HIV, CES-D, PSS, Beliefs in the Benefits of "Sutherlandia", African Culture Related Questions, and Patient Satisfaction with Study) will be translated and culturally adapted into isiZulu using standard methods [54,55] that have been successfully used in other international studies. [56,57] The goal will be to retain the conceptual equivalence of questions rather than to perform literal translations. Two independent forward translations and three independent backward translations will be performed by Zulu speakers fluent in English. Consensus meetings will be held after each step to resolve discrepancies and a harmonization committee composed of the investigators, Zulu translators, and project staff will evaluate the final Zulu version of all measures.

Cultural adaptation and translation will result in a Zulu version of all of the measures that will be appropriate to the local lifestyle and activities common to the survey area. Questions that have little cultural relevance such as those describing moderate physical activities as 'carrying groceries or bowling' might be translated as 'washing clothes or moving a bucket of water.' As a unit of distance, 'walking a city block' might be translated as 'walking the length of a soccer field.' Psychological concepts presented as 'feeling full of pep' and 'feeling down in the dumps' are likely too idiomatic for meaningful literal translation and will likely be translated as 'full of life and energy' and 'so depressed that nothing could cheer you up.' The comparability of the described concepts in English and isiZulu translations will be verified by translators.

- **Pilot Testing of Measures**

Once all of the measures have been adapted, a small pilot study (n = 20) will be conducted to ensure the acceptability of the cultural and language translations. Participants for this pilot study will be recruited from the Edendale Hospital and will meet the inclusion criteria for the study. Participants will be asked to complete all of the adapted questionnaires. At the follow-up visit two weeks following baseline, participants will complete translated measures again and be debriefed about the experience of completing the questionnaires, the acceptability and relevance of the content, and the difficulty/ease of administration. Data collected in this 2 week pilot study will be used to establish the reliability (i.e., internal consistency, test-retest) and acceptability of each measure. The pilot study will be done before onset of the study program.

- **Quality of Life Measure**

The Medical Outcomes Study HIV Health Survey (MOS-HIV)[56] is a brief, comprehensive measure of health-related quality of life used extensively in HIV/AIDS clinical studies. This 35-item questionnaire takes approximately five minutes to administer and includes ten dimensions: 1) health perceptions, 2) pain, 3) physical, 4) role, 5) social function, 6) cognitive function, 7) mental health, 8) energy, 9) health distress and 10) quality of life. Subscales are scored on a 0-100 scale (higher scores indicate better health) and physical and mental health summary scores can be generated.[58] The MOS-HIV has been shown to be internally consistent with Cronbach's alpha coefficients exceeding 0.70 for the total scale, > 0.90 for the health summary score, and > 0.91 for the mental health summary score. Numerous studies have also established that the MOS-HIV correlates with concurrent measures of health, discriminates between distinct groups, predicts future outcomes and is responsive to changes over time. It is available in 14 languages and has been included as a secondary measure in numerous clinical studies for all stages of disease. In several studies it has detected significant differences between treatments; in some cases concordant with conventional endpoints and in others discordant. [59-63]

- **Potential Mediators**

Several self-report measures will be used to assess potential mediating variables, which are presumed to be affected by *Lessertia frutescens* (syn. "Sutherlandia").

Depressive symptoms. The Center for Epidemiologic Studies Depression Scale (CES-D)[64] will be used to assess depressive symptoms. The scale has been tested in numerous settings and found to have adequate reliability (Cronbach's alpha = .85-.90). Validity was established by patterns of correlations with other self-report measures, clinical ratings of depression, and other variables which support its construct validity. The questions are based on a 4-point Likert scale (0 "not at all" to 3 "always") and are designed to ascertain the participant's level of depressive symptoms. An example is "How often (in the past week) did you feel that your life had been a failure?"

Stress. The Perceived Stress Scale (PSS)[65] will be used to measure the degree to which situations in participants' life are appraised as stressful. This is a 14-item self-report scale designed to assess how unpredictable, uncontrollable, and overloaded respondents find their lives. The PSS has good reliability (Cronbach's alpha = .85) and validity has been established via correlations with physical symptoms, depressive

symptoms, and life events. Typical questions include "In the past month, how often have you felt that you were unable to control the important things in your life?" or "How often have you felt that things were going your way?"

- **Background/Moderating Variables**

The following measures will be used to assess potential moderating variables:

Beliefs in the benefit of Sutherlandia. We have developed a 12-item questionnaire that assesses beliefs about "Sutherlandia's" impact on their HIV disease. Participants are asked to indicate their belief with each of the statements using a 5-point Likert-type response format that ranges from "definitely true" to "definitely false." A sample question is, "Sutherlandia" is a very helpful herb/muthi".

Medical history. Patients' medical history will be assessed via chart review and self-report. Data collected will include: date of HIV diagnosis, current medication regime (if any), and hospitalization history.

Medical symptoms. HIV-related medical symptoms will be assessed using open-ended questioning about self-report of frequency and severity of symptoms experienced in the past week The tertiary outcome, length of infections, will be determined based on these open-ended questions.

Demographics. Data on participants' age, gender, home language, education, relationship status, housing status, employment status, and number of children will be collected.

Alcohol/substance use. Provider interview at screening will be used to establish alcohol and substance use eligibility for participation in the study. For those who qualify, the SA – Alcohol Substance Inventory, the alcohol/drug use section of the Addiction Severity Index (ASI)[70], will be used to assess frequency and amount of use of various types of substances (e.g., alcohol and marijuana); current and past patterns of use; and participation in substance use treatment at Visits 1, 3, 5, 8. Data from this measure will be used to quantify the degree of severity of past substance use which will be used as a moderator variable in outcome analyses. This instrument has been used widely in treatment and research settings, and it has established reliability and validity across various populations.

African Culture Related Questions. This 11-item measure was developed to assess participants' familiarity with typical signs/symptoms of traditional illnesses. Items were developed based on focus groups and individual consultation with Traditional Health Providers. Participants are asked to indicate how often they are or have experienced each symptom using a 5-point Likert-type response format ranging from "never" to "very" often."

Patient satisfaction with study. This is a 10-item self-report measure of patients' satisfaction with participation in the study. Participants are asked to respond to items such as "Overall, how satisfied were you with your participation in the study?" ("not satisfied at all" to "very satisfied").

5.5.6 HIV Disease Progression Measures

Patients' CD4 cell counts and viral load will be assessed at screening and at weeks 12 and 24.

5.6 Strategies to Maximize Adherence to Study Procedures

Adherence will be assessed by returned pill count at each study visit and / or plasma and /or urine canavanine and pinitol/SU1 levels at week 12 and week 24. Unannounced pill counts will be obtained by contacting subjects by phone between week 4 and 8, and again between week 8 and 12.

The methods previously used to maximize the proportion of study participants who routinely keep their appointments in previous studies will be used in the present study. First, only individuals with an expressed interest in participating and keeping the scheduled appointments will be included. Second, information that will be collected at enrollment includes participant name, detailed address, telephone number, and names and telephone numbers of friends and relatives who would normally know the whereabouts of the study participant. Third, participants will be given a written appointment card with the date and time of the next scheduled visit. Fourth, participants with telephones will be called the day before the scheduled appointment to remind them of the appointment. Fifth, participants who miss their appointments will be contacted either by going to the place of residence or through the friends and relatives whose names are supplied at enrollment. Participants will be offered a subsistence allowance of R150 (US$25.00) paid after successful entry into the clinical study and at each subsequent visit, as compensation for time spent in the study and to cover travel expenses.

At each visit participants will be counseled on the importance of safer sex and the use of either dual contraception or abstinence will be documented.

5.7 Criteria for discontinuation

Participation in the study is voluntary. Study subjects are free to withdraw from the study at any time. If they decide to withdraw from the study, they should inform the study doctor immediately. The study doctor should not be upset or penalize the study subject in any way, and their future care will not be affected. If a study subject withdraws from the study, the study data collected before the withdrawal may still be processed along with other data collected as part of the study.

In addition, the following circumstances may lead to the ending of a study subject's participation in this study:

- medical reasons including pregnancy;
- noncompliance of a study subject; e.g. a study subject not taking their medication or not regularly keeping to the study appointments, or if they do not follow the doctor's instructions;
- a study subject taking concomitant traditional medication during this study;
- if there are not enough patients in the study;
- if the Medicines Control Council or the Biomedical Research Ethics Committee, University of KwaZulu-Natal or the Committee for Clinical Trials, University of Stellenbosch stops or suspends the investigation;
- if the CD4 count is less than 350 cells/µL at weeks 12, and this finding is confirmed another CD4 count taken 6 weeks later (if antiretroviral therapy is not available due to the CD4 <200 cut off being enforced the participant will be counseled and invited to continue the study)
- if the Sponsor stops the study.

5.8 Assessment of study medication adherence

Adherence to study medication will be assessed using pill counts at each follow-up study visit and twice during the study (between week 4 and 8, and again between week 8 and 12) using unannounced telephone contacts. At each assessment period, adherence will be computed as follows: 100% minus the percentage of pills missed (the number of pills remaining in the dossette will be divided by 3X the number of possible doses during the defined period). At baseline, phone contact information will be obtained and subjects will be trained to perform pill counts as they will do during the unannounced telephone assessment contact. Serum and urine will be frozen at baseline, weeks 12 and week 24 to assess the presence of Sutherlandia metabolites. These data may be used to augment assessment of study medication adherence.

6 INVESTIGATIONAL PRODUCT

6.1 Investigational Products and Treatments

All study treatment supplied are manufactured, tested, and released according to current Good Manufacturing Practice guidelines.

6.2 Treatment Schedule

6.2.1 Randomization

Participant identification numbers (PIN) will be randomly linked to either *Lessertia frutescens* dose group (i.e., 400mg BID, 800mg BID, or 1200mg BID) or placebo by the study statistician. At the baseline visit eligible participants will be sequentially assigned to a PIN and to a study arm. All clinical staff will be blinded to the study drug / placebo assignment, and the study statistician will be located off-site at the University of Missouri - Kansas City. Participants will be un-blinded only after written request by the Principal Investigator or as required by the Data and Safety Monitoring Board.

6.2.2 Study Product Dosing

Participants will take either the test product or placebo in capsule form twice daily with food. Participants will take three capsules, twice daily, either as 1 x 400mg *Sutherlandia* (active) capsule and 2 placebo capsules; 2 actives and 1 placebo; 3 active capsules; or 3 placebo capsules.

6.2.3 Study Duration

Participants will take either the test product or placebo for 24 weeks.

6.3 Identity of Study Products

The test product, *Lessertia frutescens* will be obtained in bulk directly from the he raw plant material was purchased from Afriplex. Afriplex (Pty) Ltd, (P O Box 3186, PAARL 7620. email: info@afriplex.co.za www.afriplex.co.za) , and we had it packed into capsules by a GMP certified manufacturer - Ferlot Manufacturing Packaging (Pty) Ltd (P O Box 1662, Jeffreys Bay 6330, email: valuedlife@epweb.co.za

Validation of the identity of the species used in the product will be performed by the Phytochemical Core of TICIPS. The final product is a standard (20mm x 6 mm) capsule filled with a pale green powder. Before use, the quality of the product will be validated by pharmaceutically evaluating samples of the capsules for mass and content uniformity and dissolution profile (using canavanine and /or pinitol as markers). The product will then be repackaged (in quantities of 60) into containers identical to that used for the placebo. Each capsule contains 400mg of the *Lessertia frutescens* (PN1^{TM} /SU1 chemotype) leaf powder. A placebo product identical in appearance will be prepared by filling the capsules with a mixture of lactulose, starch and a small amount of dried lettuce leaf powder. Preparation of the placebo and validation of the test product will be done at the Pharmaceutics Laboratory, School of Pharmacy at the University of the Western Cape. The South African Medical Research Council will assay the test product for canavanine and pinitol using a validated LCMS assay and gas chromatographic assay, respectively. A certificate of analysis will be issued before the test product is used. All study medications/placebo will be prepared, stored and dispatched by a pharmacist attached to the School of Pharmacy, at UWC.

6.4 Storage and Accountability

All study drugs will be kept in a secure place under adequate storage conditions – protected from moisture and light. Records of dispensing and returns will be maintained by the investigational center. The subject must return all unused study medication for each treatment period to the investigational center to be reconciled.

6.5 Allowed medication

Preferably no other medicine should be taken by the subjects. The use of any incidental medication (e.g. mild analgesics, oral contraceptives, etc) will be recorded.

6.6 Compliance

Compliance will be assessed by counting remaining pills returned at each follow-up visit and by two unannounced pill count by phone during the 6 month trial.

Each monthly supply of capsules will be supplied in bottles and packaged at the research unit into b.i.d. 4 week plastic dosette containers marked with the following label:

Bottle label for packaging dosettes

Study code: (TICIPS002) Investigator: Dr. Douglas Wilson

Subject Number: XXXXXXX Week Number: XX Bottle Number: X

Capsules containing Sutherlandia or Placebo

This Investigational Product Should be Stored out of the Reach of Children and Used by Study Participant Only.

<u>Directions:</u> Put one capsule from each bottle into each a.m. box and one capsule from each bottle into each p.m. box.

Emergency Contact Phone Number Edendale Exchange 033 395 4911 Contact Researcher On Call

For clinical study use only

Store below 30°C Exp Date: To be advised Batch number: xxxxxxxxxxxx

Dosette label:

Study code: (TICIPS002) Investigator: Dr. Douglas Wilson

Subject Number: XXXXXXX Week Number: XX Bottle Number: X

Capsules containing Sutherlandia or Placebo

This Investigational Product Should be Stored out of the Reach of Children and Used by Study Participant Only.

Directions: **Take the three capsules from the a.m. box in the morning and take the three capsules from the p.m. box at night**

Emergency Contact Phone Number Edendale Exchange 033 395 4911 Contact Researcher On Call

For clinical study use only

Store below 30°C Exp Date: To be advised Batch number: xxxxxxxxxxxx

7 STATISTICS

7.1 Statistical Design / Model

Appropriate descriptive analyses will be performed to examine distributional characteristics for collected measures, and to summarize changes over time as a function of group assignment. During this initial stage, we will explore bivariate relationships among primary and secondary outcome measures and variables thought to impact these outcomes (Potential Mediators and Background/Moderators). In addition, we will conduct appropriate analyses (Chi-Square, Mann-Whitney and Independent T-test for variables measured at the nominal, ordinal and interval/ratio level, respectively) to determine whether the randomly assigned groups are equivalent at the start of the study with respect to the demographic and other measures collected at baseline. Before hypothesis-testing analyses are conducted, exploratory analyses will be performed to examine the effect of various mediators and moderators on the relationship between study arm and clinical outcome. The results of these analyses will determine what additional variables will be incorporated in the subsequent hypotheses testing analyses (e.g., linear regression, mixed modeling, repeated-measures ANOVA, or analysis of covariance, as appropriate). As described in the research design section, outcome measures will also be dichotomized according to specific definitions of "failure", and failure rates compared between groups. In addition, exploratory analyses will be conducted to examine significant predictors of failure for these outcomes. A series of logistic regression models will be fitted to obtain odds ratios (OR± 95% CI) for failure if indicated by bivariate analyses.

7.2 General

The primary aim of the study is to determine the safety of *Lessertia frutescens* (Sutherlandia) used by HIV-1 infected adults. The primary outcomes for safety in stage 2 are weight loss and change in CD4 count. This study will use a two-stage, statistical selection theory design[73, 74] to explore the effect of different doses and select the "maximally tolerated and effective dose arm" of Sutherlandia for subsequent comparison against a placebo control. Subjects will be randomly assigned to receive either placebo, 400 mg BID, 800 mg BID or 1200 mg BID. An interim analysis (described below) to determine the superior active treatment arm to be continued will be conducted after 8 subjects per arm have completed the 24-week dosing regimen; this represents approximately 1/4 of the projected total number of study participants. If there are no clear differences between any of the treatment arms and the placebo arm at this point, the study will continue until 12 subjects per group have completed the 24-week dosing regimen and the interim analysis is repeated. At this point, the study will be stopped if the interim analysis identifies either significant safety issues (e.g., weight loss or CD4 counts) or demonstrated futility (e.g., active treatment being worse than placebo on any of the primary outcome measures). Following the interim analysis, accrual to the selected active and placebo control arms will continue until a sufficient sample size of 48 completed subjects per group is obtained.

For the purposes of designing the proposed study, "failure" is defined for the outcome measures. Weight loss failure is defined as having weight loss greater than 5% of body weight. Based on results reported by Morgan et al (1998)[75], 59.6% of HIV infected individuals have weight loss greater than 5% of body weight over a 6 month period. This rate includes 46.5% who have a weight loss rate of between 5% and 10%, and 13.1% who have a weight loss rate of more than 10%. Criteria for establishing failure rate for change in CD4 cell count was determined based on the following rationale and criteria. Existing research on un-medicated HIV+ participants in Cape Town, South Africa (with baseline CD4 counts 351-500) has demonstrated that the average CD4 cell count decline over a 6 month period in this population is 13.7 cells (95% CI 16.7, 10.6). Therefore, a CD4 cell count decrease of more than 20 cells across the 24 week period will be considered a treatment failure. For the secondary outcome Quality of Life (QoL) subscales of the MOS-HIV, failure was defined based on previously published[51, 52] documented differences in subscale scores (means $\pm$ SD) for populations with CD4 counts 200-500 (referent group) and populations with ARC (comparison group). For each of the subscales, we defined a failure in the respective QoL scales, as a decrease in score of 20 points or more from their baseline value. Finally, failure for the tertiary outcome, Length of Infection, was defined based on data from a recent Phase I clinical study that examined the safety of Sutherlandia (compared to a placebo) in a healthy population of individuals in South Africa. Results indicated that while the number of infections reported by each group in a 24-week period was not different, the average length of infection per event was significantly less for the Sutherlandia group (average 4 days versus 8 days). Therefore, failure for the outcome Length of Infection in the proposed study is defined as having more than 10 days of infection per event on average. This outcome will be assessed using open-ended questions during the evaluation of medical symptoms.

For the interim analysis, the strategy described by Steinberg and Venzon (2002)[74] will be used to determine which of the three active treatment arms (400 BID, 800 BID or 1200 BID) will be selected as superior for continued evaluation in the second stage of the study. Given the expected base rate for the various outcome measures, a reduction of 15% in the proportion of failures in an active treatment arm compared to the placebo arm will be considered a meaningful improvement. This strategy will give us a 90% probability of selecting the superior active treatment arm for continuation into the second stage of the study (Simon et al. 1985).[73] For example, a reduction of at least 15% in the rate of weight loss failure in any of the active treatment arms would be meaningful. Criteria established by Steinberg et al. (2002)[74] will be used to select the superior treatment as follows. Once 32 subjects (8 per stage 1 treatment arm) have completed the 24 week study, data will be evaluated to determine the number of failures in each group for a given outcome measure. Based on the number of failures per treatment arm, the gaps between group failure rates will be determined. A gap of 2 in failure rate between treatment groups is the minimal difference needed for determining which active treatment arm should be selected for continuation. To be selected, an active treatment arm must demonstrate a gap of at least 2 when compared to the placebo arm as well as when compared to the other active treatment arms. If the minimal difference in failure rates between arms does not exist at this point, accrual will continue until 12 subjects per group are completed and a similar strategy used for determining the best active treatment arm for continuation. Again, a gap of 2 subjects categorized as failures between arms at this point would be the minimal difference in failure rates for the active treatment arm selection.

In the event that there is an equivalent weight loss failure rate in more than one active treatment arm, and this rate is at last 15% better than the failure rate in the placebo arm, the failure rate for change in CD4 cell count will be used as the primary determinant for selecting the treatment arm to continue with. The arm with the lowest failure rate on this additional outcome measure will be selected. In the event there are no differences in the failure rate among more than one active treatment arm, and these rates are better than the rate in the placebo arm, the active treatment arm with the highest tolerable dose will be selected for continuation.

The estimates used to define criteria for the interim analyses were also used to determine that a sample size of 48 completed subjects per group will be adequate to assess the safety of Sutherlandia after 24 weeks of treatment (Simon et al, 1985).[73] This sample size is planned to result in a final sample of 96 (48 subjects per group) for stage two. We are confident that this sample size will be sufficient to detect meaningful differences in the primary outcome variables as well as the secondary and tertiary outcome variables. Randomization with substitution will be used until 48 subjects per arm have completed the 24 week study.

For the primary outcome Length of Infection, we also performed a traditional power analysis to determine the sample size that would be necessary to detect a significant reduction in average length of infection. Effect size estimates were computed based on the recent Phase I study data. Once again, we assume a one-tailed alpha of .05 and provide for 80% power to detect indicated effect sizes between the active and placebo control groups.

The estimated sample size of 48 per arm will provide more than sufficient power to detect significant differences between the active and placebo arms.

**Primary Outcome**	**n/gp**	**Expected Difference in Failure Rate**	**Probability in Selecting Superior Treatment**
Length of infection (days)	36	4.3 (2.2)	8.0 (5.9)
**Secondary Outcomes (QoL)**		**Mean (SD) CD4 200-500**	**Mean (SD) ARC**
Energy	25	64.4 (20)	45.4 (22)
Physical Function	25	89.0 (20)	70.0 (24)
Mental Function	36	65.4 (21)	50.0 (22)
Distress	30	69.0 (26)	49.0 (27)

Secondary and Tertiary Outcomes

For the secondary QoL outcome, we utilized both statistical selection theory and traditional sample size calculations for the QoL subscales of the MOS-HIV to assess power. Because our hypothesis predicts an increase in scores on the QoL subscales, we assume a one-tailed alpha of .05 and provide for 80% power to detect indicated effect sizes for the final two study arms (selected active and placebo). Effect size estimates were determined based on conservative extrapolations of population values determined by Wu and colleagues[51] and by Mast and colleagues[52] for the subscales most likely to be affected by treatment: Energy, Physical Function, Mental Function and Distress. Sample size estimates are provided below. Our estimated sample size of 96 (48 subjects per arm) is more than sufficient to meet these assumptions and provides sufficient power to detect meaningful change in the 4 subscale scores.

7.3 Planned Analyses

Besides the interim analysis, a single statistical analysis (of one treatment group vs placebo) will be performed at the end of the study. An intention to treat (ITT) approach will be followed, i.e. statistical analysis of safety will be based on data from all patients who were randomized and from whom meaningful data were collected. Data will be displayed graphically for visual inspection. Descriptive statistics will be presented as means ± SEM, and ninety percent confidence levels of the means.

Baseline characteristics. The subject disposition will be summarized. The demographic, background and baseline data will be presented descriptively.

Analysis of Safety. Adverse Events, as reported throughout the course of the study will be listed individually, per treatment group. Pre-study and post-study findings of **physical examination, vital sign variables, laboratory variables** (haematology, clinical chemistry and urinalysis), and **12-lead ECG** will be listed individually and summarized; values outside the normal range will be listed. Protocol violations will be listed per subject, describing the nature of the violation. Subjects failing to complete the study (as well as the times and reasons for discontinuation) will be displayed.

Analysis of Constituent levels. The active constituents obtained during the treatment will be compared to that found before the start of study treatment, and the averaged levels obtained in the study medication and placebo groups will be compared and analyzed using an analysis of variance model.

8 ETHICS

8.1 Ethics review

The study will not be initiated before the protocol and informed consent and subject information form have been reviewed and received approval from the Independent Ethics Committees (IEC) and Regulatory Authority. The final study protocol, including the final version of the Subject Information and Consent Forms, must be approved in writing by an IEC (e.g. the **Biomedical Research Ethics Committee,** University of KwaZulu-Natal) and the MCC before enrolment of any subject into the study. The Principal Investigator is responsible for informing the IEC of any serious adverse events (SAE) and amendment to the protocol as per regulatory requirement.

8.2. Ethical conduct of the study

The study will be performed in accordance with the ethical principles in the Declaration of Helsinki (see Appendix 14.1) and that are consistent with Good Clinical Practice and applicable regulatory requirements.

8.3 Subject information and consent

Prior to subjects' participation in the clinical study, written informed consent will be obtained from each subject according to the regulatory and legal requirements. The Investigator will ensure that the subject is given full and adequate oral and written information about the nature, purpose, possible risk and benefit of the study. Subjects must also be notified that they are free to discontinue from the study at any time. The subject should be given the opportunity to ask questions and allowed time to consider the information provided. The subject's signed and dated informed consent must be obtained before conducting any study specific procedure. The investigator must store the original, signed Subject Informed Consent Form and a copy must be given to the subject. Samples of the English version of the Subject Information and Consent Forms are enclosed (Appendix 14.2). Zulu translations of the approved English version will also be provided and submitted to IEC.

8.4 Subject data protection

The Subject Information and Consent Form will explain that study data will be stored in a computer database, maintaining confidentiality. Subjects in this database will be identified by initials or enrolment code / subject number only. Authorized representatives of a regulatory authority (e.g. MCC) may require direct access to parts of the clinical site records relevant to the study, including subjects' medical history for data verification purposes.

The Investigator must keep a Subject Identification List of all subjects that have signed the informed consent, including subject number, full name and last known address.

8.5 Insurance and Indemnity

All subjects participating in this study will be covered for any study medicine-related adverse effects through insurance cover to be taken out by TICIPS. Compensation for any research-related injury caused by taking part in the study will be in accordance with the guidelines of the Association of the British Pharmaceutical Industry (ABPI) (Appendix 14.).

9 HUMAN SUBJECTS

The study will be conducted with ethical oversight from the Research Ethics Boards of the Universities of KwaZulu-Natal and Western Cape (for clinical trials, the latter falls under the umbrella of the Committee for Pharmaceutical Trials at the University of Stellenbosch) and Institutional Review Boards of the University of Missouri and the University of Missouri-Kansas City, with regulatory oversight from the South African Medicines Control Council. The study investigators will ensure that voluntary informed consent is provided by study participants indicated by signature or witnessed thumbprint on the informed consent document (in triplicate). Study participants can withdraw consent at any time. The study will be conducted in accordance with Good Clinical Practice. Insurance and indemnity coverage will be purchased to cover study-related adverse events.

9.1 Risks to the Participants

9.1.1 Human Participants Involvement and Characteristics

This is a double blind randomized controlled clinical study in which we propose to enroll 120 participants to evaluate the safety of *Lessertia frutescens* and its impact on quality of life, CD4 cell count, and viral load. The participants will be HIV infected individuals (age 21 years or older) with early HIV disease ($\geq$350 CD4 cells/µL; Viral load >1,000 copies/mL). Participants must have the cognitive capacity to provide informed consent. Individuals with an acute opportunistic infection or other acute medical illness will be excluded, but can participate upon resolution of acute symptoms. Pregnant women will be excluded as well as any vulnerable populations.

9.1.2. Sources of Materials

Data will be collected from participants via self-report questionnaires and medical interviews. Data will also be abstracted from participants' medical records. Blood samples will be obtained as described in section 5.5.4. Research data and specimens will be used only for the proposed research. Unsolicited clinical information disclosed by participants to study personnel that may be clinically relevant, will not be recorded but will be forwarded to appropriate clinic staff.

9.1.3 Potential Risks

The risk of physical harm is minimal for participants in this protocol. The study procedures have been designed to complement but not interfere with routine medical care, thus there is little potential for adverse events directly related to study procedures. Participants may feel discomfort or pain associated with venipuncture, and less commonly they may become dizzy, faint, bleed, or get a bruise at the site. For each participant there will be blood draws prompted by the study protocol. These risks are similar to those done during routine care, and all blood draws will be performed by a trained nurse or medical practitioner.

The study design may introduce risks related to the use of *Lessertia frutescens*. However, this plant is widely used in southern African traditional medicine. It has been found to be safe in non-human primates and in a Phase 1 study in healthy HIV-negative volunteers. Study participants will be evaluated for evidence of toxicity and adverse events during the course of the study, at each study visit.

Psychological and social risks may be associated with participation in the study. It is possible that participants could experience distress or discomfort caused by discussing issues related to their HIV status and other emotionally charged topics related to study materials. Patient confidentiality and privacy could be jeopardized by receiving handout materials that discuss HIV, attending study visits, or other associations with study personnel or materials. While unavoidable, these risks are similar to that experienced by any HIV infected patient receiving routine care.

9.2 Adequacy of Protection Against Risks

9.2.1 Recruitment and Informed Consent

The study protocol, informed consent form, and advertisement flyers will be submitted to the Research Ethics Boards of the Universities of KwaZulu-Natal and Western Cape (for clinical trials the latter falls under the umbrella of the Committee for Pharmaceutical Trials at the University of Stellenbosch), with regulatory oversight from the South African Medicines Control Council for review and approval prior to participant enrolment as well as from the Institutional Review Boards of the University of Missouri and University of Missouri-Kansas City. Because all patient contact will be done in South Africa, HIPAA requirements regarding safeguarding patients' private medical information do not apply. All patients who are eligible will be informed of the study opportunity during a clinic visit. If the patient is interested in learning more they will be directed to study staff who will ensure that the participant meets inclusion/exclusion criteria and discuss the protocol in more detail with the patient. Study staff will ensure that the patient is thoroughly informed about the study. This will be accomplished by reading the informed consent form (in the patients' preferred language) with the potential participant in order to describe the details of the study including the purpose of the research, the sponsors of the project, the nature of the patient's involvement, the probability of being assigned to one of the 4 different groups, alternatives to participating in the study, the possible risks and benefits of the study, the extent to which participant confidentiality will be protected, and a patient's right to decline or withdraw participation without penalty. The patient will have the opportunity to ask questions and may request additional time for consideration. Patients who agree to participate in the study will sign the informed consent form. The original informed consent form will be stored in a locked file cabinet and one will be given to the participant. These procedures will not delay or interfere with the patient's medical treatment plan.

9.2.2 Protection Against Risk

The proposed study will receive human subjects' protection review and approval prior to participant enrolment. This and the use of informed consent will generally assist in the protection of study participants. Experienced clinic staff at the clinical site are involved as project personnel and are responsible for overseeing general clinical safety issues of the project as well as being a contact to handle clinical situations for individual participants.

To curtail psychological and emotional stress the professional nurse/ investigator administering the quality of life questionnaire are trained to avoid confrontation and use techniques which may allay stress during sessions. Participants will be assured they may skip evaluation items they do not want to answer. The study staff will be carefully trained to protect participant confidentiality. All interviews will be conducted at the study site. They will devise a plan with the participants for appropriate means of making contact with the participant. For example, what phone numbers they may use and whether they may leave a message. Handout materials will be modestly designed and special care will be taken to omit any reference to HIV on any documents that contain participant identifying information. Participants will be assigned a study number and all collected data will be marked with study number only. The informed consent forms and master list of patient name matched to study

number will be stored separately from the study data in a locked file cabinet. Only appropriate study staff will have access to study data.

9.3 Potential Benefits of the Proposed Research to the Participants
It is possible that participants enroled in this study will not directly benefit from their participation. Some participants may benefit from the repeated viral load and CD4 assessments made during the course of the study, which is more frequent than that funded by the South African government. All participants will receive compensation for completed evaluations up to a total of R1950 for participation in the study.

9.4 Importance of the Knowledge to be Gained
This project will contribute to understanding the safety of *L. frutescens* when used by HIV-infected South African adults with early disease. Many southern African HIV positive adults are deciding to use traditional medications in an attempt to favorably modify the course of the infection, and the results from this study will allow patients and their healthcare providers to make evidence-based decisions. Given the catastrophically high number of southern Africans living with this infection the results of this study may be of substantial and durable value.

9.5 Collaborating Sites
All the staff contributing to this study will receive training in Good Clinical Practise or Good Laboratory Practise and be expected to successfully complete the NIH course in medical ethics. All the sites will adhere to the standards of research contained in these courses.

9.6 Women and Minority Inclusion in Clinical Research
This study will recruit HIV positive adults receiving care from institutions in the Edendale Hospital area (District 22, KwaZulu-Natal). It is therefore anticipated that the majority of participants will be Zulu-speaking and, given the demographics of the epidemic, that more women than men will enrol. In line with South African affirmative action policies every effort will be made to recruit Zulu-speaking study staff. Translators will be used to support study staff who do not speak Zulu well. Potential participants from other language groups will not be excluded.

9.6.1 Inclusion of Women
Pregnant HIV infected women will be excluded at baseline from this study. Women enroled on the study will receive counseling on safer sex and contraception and will be referred to relevant healthcare providers. Women who fall pregnant while on the study will be counseled on possible teratogenic effects to the fetus and on the use of antiretroviral therapy for the prevention of mother to child HIV transmission, and exited from the study, and be asked to return for follow-up visits to establish outcomes in mother and infant. All other adult women meeting inclusion/exclusion criteria will be eligible for participation. Pregnant women will be eligible for participation after pregnancy if they meet the study inclusion criteria.

9.6.2 Inclusion of Children
Children will not be included in the study. As the South African National Child Act is widely interpreted by local IRBs as requiring parental consent for research participants under the age of 21, children under the age of 21 will not be included in this study. It is likely that many of these children under the age of 21 have not disclosed their HIV+ status to their families as doing so often results in negative consequences. Due to the stigmatization that surrounds HIV infection the investigators decided to exclude children under the age of 21 who would need to disclose their HIV status to their parents in order to participate rather than asking them to expose themselves to potential negative consequences simply to participate in the clinical study.

9.7 Data and Safety Monitoring Plan
Due to the widespread use of *L frutescens* in southern African we do not expect a high incidence of adverse events in this Phase 2 study. In accordance with NIH guidelines [http://grants.nih.gov/grants/guide/notice-files/NOT-OD-00-038.html], however, a Data and Safety Monitoring Board will be set up by TICIPS and utilized by this study. A data-monitoring plan will be formulated by the study investigators which will meet by teleconference and will observe participant enrolment and withdrawal rates and monitor adverse events.

Several mechanisms have been put in place to ensure data integrity. All interviewers will be trained to promote standardized and objective collection and recording of participant information. Interview instruments are edited immediately after the interview by both the interviewer who conducted the interview and by a second interviewer for legibility, consistency, and completeness. Errors in legibility, consistency, and /or completeness

are reviewed with each interviewer. Clinical, laboratory and interview data are double entered and imported into a password-protected database that is backed up through a secure off-site connection. We have set up several mechanisms to ensure the confidentiality of data collected from the participants. All paper files are stored in locked file cabinets, and electronic files are stored in password-protected files. Furthermore, both paper and electronic files, including laboratory reports, are identified only by a participant's ID number (PIN) and subject initials. Identifying information linking participants to their PIN is retained off site in a locked cabinet accessible only by the Principal Investigator, Project Director, and Project Coordinator.

9.8 Vertebrate Animals
Non-human vertebrate animals will not be studied.

9.9 Minorities and Women
Women and minority groups will be included in this study. Pregnant women will, however, be excluded at screening, and women will be tested for pregnancy at each study visit.

10 STUDY DOCUMENTATION AND ARCHIVING OF DATA

The following types of forms will be used in this study:

- Participant information leaflet
- Informed consent forms will be available in Zulu and English.
- Study Source Document
- Case record forms (CRF) that document enrolment, follow-up visits, and specimen collection.
- Administrative forms that document participant screening, enrolment, termination from study.
- Self report questionnaires in Zulu and English

The clinical data collected in the Source Document will be transcribed into a CRF and then double entered into a custom-designed database.

10.1 Data Collection Form Completion and Submission
All CRFs will be completed and data will be double entered according to instructions that will be developed in a detailed Manual of Procedures. A participant's name will be collected only once, and this information will be kept on a form that does not contain any test results, and that is filed separately from forms that do contain test results. The initials of the staff member who completes the form will appear at the bottom of the first page of each form. All forms will be reviewed prior to data entry for accuracy, consistency, and completeness by designated study site staff.

10.2 Data Management
Each participant will be assigned a unique study participant identification number (PIN). This number and their initials will be recorded on each data form and clinical specimen to facilitate linkage of data. Names and other obvious identifiers will not be used on forms, and will only be used on clinical specimens that are drawn during unscheduled acute care clinic visits. Laboratory specimens will be collected from the study sites each afternoon and delivered daily to the laboratories.

The site administrator (study coordinator) will maintain logbooks to record details of patients screened for the study, participants enrolled onto the study, and the dates of completed and upcoming clinic visits and specimen collections. In addition, a study file will be maintained for each participant, which will contain all forms, lab results and other pertinent study information. All logbooks and records will be kept in locked filing cabinets when not in use and will be accessible only by clinic personnel. Laboratory tests will be requested on study laboratory test requisition forms. In the laboratory the test results will be recorded and the results printed out three times: one copy will be kept in the laboratory, one copy in the study case record folder and one copy in the hospital folder. Forms received in the study management room will be logged in upon receipt. The forms will be entered and verified, and the date of these procedures will be recorded on the log-in sheet. Within two years of completion of the final study report, identifiers excluding the PIN will be deleted from computerized and paper data files.

10.3 Record, Storage and Archive

The study on-site director and the data manager will be responsible for maintaining and storing of all CRFs. Source records may include laboratory requisitions and reports, clinical notes, and documentation of referrals. All data collection forms and source records will be stored in locked files in a secure area. All locator information will be kept in locked rooms in a secure area apart from all other study documents. Access to study records and data files will be limited to study personnel.

11 DATA QUALITY ASSURANCE

Monitoring and auditing procedures, as determined will be followed, in order to comply with Good Clinical Practice and to ensure acceptability of the study data for international publication purposes.

Data from the study will be collected in CRFs. Data editing will be performed at the study center, comparing source and CRF entries. Data will be entered in a blind mode.

During the study an independent monitor will visit the investigational site to confirm that the facilities remain acceptable, that the investigational team is adhering to the protocol and that data are being accurately recorded in the CRFs. Source data verification (a comparison of the data in the CRF with the subjects' laboratory test results and other source documents) will also be performed.

Authorized representatives of the regulatory authority (e.g. MCC) may visit the center to perform inspections, including source data verification. A quality assurance audit of this clinical study may be conducted by the sponsor or sponsor's designees. The quality assurance auditor will have access to all medical records, the investigator's study related files and correspondence, and the informed consent documentation that is relevant to this clinical study.

Clean File for the final database will be declared when all data have been entered and a quality check on a sample of the data has been performed. The database will be locked after Clean File has been declared and data extracted for statistical analysis. Treatment code will not be broken until clean file.

Study committee meetings will be held as needed prior to or during the study. The medical, nursing and other staff involved in the study will receive proper education/information on how to conduct the study according to the protocol.

12 STUDY TIME TABLE AND TERMINATION

First Subject In	May 2008
1st Interim analysis	August 2009
2nd Interim analysis	August 2010
Last Patient Out	December 2010
Clean File	February 2011
Study Report	April 2011

13 REFERENCES

1. http://www.hsrc.ac.za/media/2003/10/20031020.pdf
2. Fauci AS et al. Immunopathogenic mechanisms of HIV infection. Annals of internal medicine. 1996; 124: 654-63.
3. Morgan et al. The natural history of HIV-1 infection in Africa. Nature Medicine. 2001; 7: 143-45
4. Leserman J. HIV disease progression: depression, stress, and possible mechanisms. Biol Psychiatry. 2003; 54: 295-306
5. Kopnisky KL, Stoff DM, Rausch DM. Workshop report: The effects of psychological variables on the progression of HIV-1 disease. Brain Behav Immun. 2004; 18: 246-61.
6. Palella FJJ, Delaney KM, Moorman AC et al. Declining morbidity and mortality among patients with advanced human immunodeficiency virus infection: HIV outpatients investigators. N Eng J Med 1998; 338: 853-68
7. Moatti JP, Spire B, Kazatchkine M. Drug resistance and adherence to HIV/AIDS antiretroviral treatment: against a double standard between the north and the south. AIDS. 2004 Jun;18 Suppl 3:S55-61.
8. Van Wyk B-E, Gericke N. 2000 Peoples Plants, Briza Publications, South Africa.
9. Van Wyk B-E, Oudtshoorn B, Gericke N. 2000 Medicinal Plants of South Africa, 2nd edition, Briza Publications, South Africa.
10. Ethnomedicine report of Sutherlandia frutescens. 2001 The International Conference on Traditional Medicine in HIV/AIDS and malaria, Abuja, Nigeria. www.astral-natural.com/Africana.htm
11. Singh RK, Pandey BL, Tripathi M, et al. Anti-inflammatory effects of (+)-pinitol. Fitoterapia 2001; 72: 168-70
12. Katere DRP, Eloff JN. Antibacterial and antioxidant activity of Sutherlandia frutescens. 2003 Paper presentation at the SAAB and ISE Conference, Pretoria, South Africa.
13. Rosenthal GA. L-canavarine: A potential chemotherapeutic agent for human pancreatic cancer. Pharmaceutical Biology. 1998; 36:194-201.
14. Brown DL, Naiki M, Gershwin ME. Does L-canavanine ingestion induce murine SLE? Paradoxical effects on survival of BALB/c. Journal of Nutritional Immunology. 2001; 5: 17-27.
15. Fernandes AC, Cromarty AD, Albrecht C, Jansen Van Rensburg CE. The antioxidant potential of Sutherlandia frutescens. J Ethnopharmacol. 2004 Nov;95(1):1-5.
16. A toxicity study of Sutherlandia leaf powder (Sutherlandia frutescens sub-species microphylla) consumption. 2002 Medical Research Council, Tygerburg, South Africa. www.sahealthinfo.org/traditionalmeds/firststudy.htm.
17. Mellors J et al. Plasma viral load and CD4+ lymphocytes as prognostic markers of HIV-1 infection. Annals of Internal Medicine. 1997; 126: 946-54.
18. Kedzierska K, Crowe SM, Turville S, Cunningham AL. The influence of cytokines, chemokines and their receptors on HIV-1 replication in monocytes and macrophages. Rev Med Virol. 2003 Jan-Feb;13(1):39-56.
19. Breen EC. Pro- and anti-inflammatory cytokines in human immunodeficiency virus infection and acquired immunodeficiency syndrome. Pharmacol Ther. 2002 Sep;95(3):295-304.
20. Abad LW, Schmitz HR, Parker R, Roubenoff R. Cytokine responses differ by compartment and wasting status in patients with HIV infection and healthy controls. Cytokine. 2002 Jun 7;18(5):286-93.
21. Saha RN, Pahan K. Tumor necrosis factor-alpha at the crossroads of neuronal life and death during HIV-associated dementia. J Neurochem. 2003 Sep;86(5):1057-71.
22. Kumar M, Kumar AM, Waldrop D, Antoni MH, Eisdorfer C. HIV-1 infection and its impact on the HPA axis, cytokines, and cognition. Stress. 2003 Sep;6(3):167-72.
23. O'Keefe EA, Wood R. The impact on human immunodeficiency virus (HIV) infection on quality of life in a multiracial South African population. Quality of Life Research. 1996; 5: 275-80.
24. Impact of African herbal medicines on antiretroviral metabolism. Mills E, Van Heeswijk R, Phillips E, Wilson K, Leonard B, Kosuge K, Kanfer I. AIDS 2005 19:95-6.
25. Letvin NL. Progress Toward an HIV Vaccine. Annu Rev Med. Aug 2004.
26. Diagnostic yield of peripheral lymph node needle-core biopsies in HIV infected adults with suspected smear negative tuberculosis. Wilson D, Nachega J, Chaisson R, Maartens M. Inter J Tuber Lung Dis (in press).
27. Identifying Sputum Smear-Negative TB in HIV-Infected Adults Using A Bacteriophage Assay. Wilson D, Nachega J, Chaisson R, Maartens G. Durban AIDS Conference 2003.

28. Validation of expanded case definitions for smear-negative tuberculosis in HIV-infected South African adults. Wilson D, Morroni C, Nachega J, Chaisson R, Maartens G. Bangkok AIDS conference 2004.
29. Effect of highly active antiretroviral therapy on incidence of tuberculosis in South Africa: a cohort study. Badri M, Wilson D, Wood R. Lancet 2002; 359:2059-64.
30. Impact of HAART in preventing tuberculosis in a South African cohort. Wood R Wilson D, Badri M. Barcelona AIDS conference 2002.
31. Kennedy, S., Goggin, K., & Nollen, N. (In Press). Adherence to HIV medications: Utility of the theory of self-determination. Cognitive Therapy and Research.
32. Kennedy, S., Goggin, K., Nollen, N., Reese-Smith, J., Terhune, N., Wise, D., & Burgess, D. (2002). Adherence to HIV medications: The role of autonomous regulation, autonomy support, and self-efficacy. Annals of Behavioral Medicine, 23(suppl.), S012.
33. Nollen, N., Goggin, K., Kennedy, S., Burgess, D., Reese-Smith, J., Terhune, N., & Wise, D. (2002). Monitoring coping style and antiviral adherence in HIV+ patients. Annals of Behavioral Medicine, 23(suppl.), S012.
34. Goggin, K., Metcalf, K., Kennedy, S., Wise, D., Murray, T., Broadus, K., Burgess, D., Reese-Smith, J., Terhune, N., Buckendahl, H., & Downes, A. (2002). HIV risk and substance use behaviors among African-American youth. Poster to be presented at the 109th Annual Meeting of the American Psychological Association, Chicago, IL.
35. Goggin, K., Metcalf, K., Wise, D., Kennedy, S., Murray, T., Burgess, D., Reese-Smith, J., Terhune, N., Broadus, K., Downes, A., & Buckendahl, H. (2002). A youth-initiated HIV risk and substance use prevention program. Poster to be presented at the 109th Annual Meeting of the American Psychological Association, Chicago, IL.
36. Murray, T., Malcarne, V., & Goggin, K. (2003). Alcohol-related God/higher power control beliefs, locus of control, and recovery within the alcoholics' anonymous recovery paradigm. Alcoholism Treatment Quarterly, 21(3), 23-41.
37. Goggin, K., Catley, D., Brisco, S., Engelson, E.S., Rabkin, J. G., & Kotler, D. P. (2001). A female perspective of life with HIV disease. Health and Social Work, 26(2), 80-90.
38. Goggin, K., Sewell, M., Ferrando, S., Evans, S., Fishman, B., & Rabkin, J. (2000). Plans to hasten death among men with HIV/AIDS: Relationship to psychological adjustment. AIDS Care, 12, 125-136.
39. Sewell, M., Goggin, K., Ferrando, S., Evans, S., Fishman, B., & Rabkin, J. (2000). Anxiety syndromes and symptoms among men with AIDS: A longitudinal controlled study. Psychosomatics, 41, 294-300.
40. Ferrando, S., Goggin, K., Sewell, E., Evans, S., Fishman, B. & Rabkin, J.G. (1998). Substance use disorders in gay/bisexual men with HIV and AIDS. The American Journal on Addictions, 7, 51-60.
41. Ferrando, S., Evans, S., Goggin, K., Sewell, M., Fishman, B., & Rabkin, J. (1998). Fatigue in HIV illness: Relationship to depression, disability and quality of life. Psychosomatic Medicine, 60, 759-764.
42. Ferrando, S., van Gorp, W., McElhiney, M., Goggin, K., Sewell, M., & Rabkin, J. (1998). Highly active antiretroviral treatment (HAART) in HIV-infection: Benefits for neuropsychological function. AIDS, 12, F65-70.
43. Evans, S., Ferrando, S., Sewell, M., Goggin, K., Fishman, B., & Rabkin, J. (1998). Pain and depression in HIV illness. Psychosomatic Medicine, 39, 528-535.
44. Goggin, K. J., Zisook, S., Heaton, R. K., Atkinson, J. H., Marshall, S., McCutchan, J. A., Chandler, J. L., Grant, I., & the HNRC Group. (1997). Neuropsychological performance of HIV+ men with major depression. Journal of the International Neuropsychological Society, 3, 457-464.
45. Zisook, S., Peterkin, J. J., Goggin, K. J., Sledge, P., Atkinson, J. H., Grant, I., & the HNRC Group. (1998). Treatment of major depression in HIV+ men. Journal of Clinical Psychiatry, 59(5), 217-224.
46. Goggin, K. J., Engelson, E. S., Rabkin, J. G., & Kotler, D. P. (1998). Relationship of mood, endocrine and sexual disorders in HIV+ women: An exploratory study. Psychosomatic Medicine, 60, 11-16.
47. Goggin, K. J., & Rabkin, J. G. (1997). Treating HIV+ women. In M. O'Connor (Ed.), Treating the psychological consequences of HIV. San Francisco: Jossey-Bass.
48. Ilaria, G., Damson, L., Goggin, K. J., & Lytton, J. (1996, July). Don't shut me out: A training video to help HIV+ parents disclose to their children. Presented at the 11th International AIDS Conference, Vancouver, Canada.
49. Nollen, N. L., Goggin, K., Heiland, M. F., Catley, D., Kessler, K., Brammer, S. K., Schmidt, C., & Murray, T. (in press). The impact of combination therapy on the health behaviors of HIV+ gay men. AIDS and Behavior.
50. Heiland, M. F., Goggin, K., Kessler, K., Brammer, S. K., Nollen, N. L., Schmidt, C. K., & Murray, T. (in press). Changes in meaning and identity for HIV+ gay men in the wake of combination therapies. Psychology and Human Sexuality.

51. Wu, A.W., Revicki, D.A., Jacobson, D., & Malitz, F.E. (1997). Evidence ffor reliability , validity and usefulness of the Medical Outcomes Study HIV Health Survey (MOS-HIV). Quality of Life Research, 6, 481-493.
52. Mast, T.C., Kigozi, G., Wabwire-Mangen, F., Black, R., Sewankambo, N., Serwadda, D., Gray, R., Wawer, M., & Wu, A.W. (2004). Measuring quality of life among HIV-infected women using a culturally adapted questionnaire in Rakai district, Uganda. AIDS Care, 16, 81-94.
53. Holmes, C.B., Wood R., Margolin, S., Badri, M., Maartens, G., Freedberg, K., & Losina, E. (2004, October). Analysis of CD4 trends in antiretroviral-naive HIV-1-infected patients in Cape Town, South Africa: implications for clinical care. Paper presented at the 42nd annual meeting Infectious Diseases Society of America, Boston.
54. Bullinger, M., Alonso, J., Apolone, G., Leplege, A., Sullivan, M., Wood-Dauphinee, S., Gandek, B., Wagner, A., Aaronson, N., Bech, P., Fukuhara, S., Kaasa, S., & Ware, J.E. (1998). Translating health status questionnaires and evaluating their quality: the IQOLA project approach. International Quality of Life Assessment. Journal of Clinical Epidemiology, 51, 913-923.
55. Ware, J.E., Keller, S.D., Gandek,B., Brazier, J., Sullivan, M., & the IQOLA Project Group (1995). Evaluationg translatins of helath status questionnaires: methods form the IQOLA Project. International Journal of Technology Assessment in Health Care, 11, 525-551.
56. Wu, A.W., Revicki, D.A., Jacobson, D., & Malitz, F.E. (1997). Evidence for reliability, validity and usefulness of the Medical Outcomes Study HIV Health Survey (MOS-HIV). Quality of Life Research, 6, 481-493.
57. Mast, T.C., Kigozi, G., Wabwire-Mangen, F., Black, R., Sewankambo, N., Serwadda, D., Gray, R., Wawer, M., & Wu, A.W. (2004). Measuring quality of life among HIV-infected women using a culturally adapted questionnaire in Rakai district, Uganda. AIDS Care, 16, 81-94.
58. Revicki, D.A., Sorensen, S., & Wu, AW (1998). Reliability and validity of physical and mental health summary scores form the Medical Outcomes Study HIV Health Survey. Medical Care, 36, 126-137.
59. Scott-Lennox, J., McLaughlin-Miley, C.J., & Mauskopf, J.A. (1996). Impact of lamivudine and zidovudine therapy on quality of life (QOL). In : XI International conference on AIDS, Vancouver, 7-12 July, 1996.
60. Revicki, D.A., & Swartz, C. (1997). Quality of life outcomes of saquinavir, zalcitabine, and combination saquinavir-zalcitabine therapy for advanced HIV-infection. 4th Conf Retroviruses Opportunity Infection, 27, 266.
61. Wu, A.W., Lichter, S.L., Richardson, W. et al. (1992, July). Quality of life in patients receiving clarithromycin for mycobacterium avium complex infection and AIDS. In: VIII International Conference on AIDS/III STD World Congress, Amsterdam.
62. Wu, A.W., Rubin, H.R., Mathews, W.C. et al. (1993). Functional status and well-being in a placebo-controlled trial of zidovudine in early AIDS-related complex. Journal of AIDS, 6, 452-458.
63. Chaisson, R.E., Benson, C.A., Dube, M. et al. (1994). Clarithromycin for disseminated Mycobacterium avium-complex in AIDS patients. Annual of Internal Medicine, 121, 905-911.
64. Radloff, L. S. (1977). The CES-D scale: a self-report depression scale for research in the general population. Applied Psychological Measures, 1, 385-401.
65. Cohen, S., Kessler, R. C., & Gordon, L. U. (1995). Measuring Stress – A guide for health and social scientists. New York: Oxford University Press.
66. Holzemer, W. L., Henry, S. B., Nokes, K. M., Corless, I. B., Brown, M., Powell-Cope, G. M., Turner, J. G., & Inouye, J. (1999). Validation of the sign and symptom checklist for persons with HIV disease (SSC-HIV). Journal of Advanced Nursing, 30, 1041-1049.
67. Kennedy, S., Goggin, K., & Nollen, N. (In Press). Adherence to HIV medications: Utility of the theory of self-determination. Cognitive Therapy and Research.
68. Kennedy, S., Goggin, K., Nollen, N., Reese-Smith, J., Terhune, N., Wise, D., & Burgess, D. (2002). Adherence to HIV medications: The role of autonomous regulation, autonomy support, and self-efficacy. Annals of Behavioral Medicine, 23(suppl.), S012.
69. Chesney, M. A., Ickovics, J. R., Chambers, D. B., Gifford, A. L., Neidig, J., Zwickl, B., & Wu, A. W. (2000). Self-reported adherence to antiretroviral medications among participants in HIV clinical trials: The AACTG Adherence Instruments. AIDS Care, 12(3), 255-266.
70. McLellan AT, Kushner, H., Metzger, D., Peters, R., Smith, I., Grissom, G., Pettinati, H., & Argeriou, M. (1992). The fifth edition of the Addiction Severity Index. Journal of Substance Abuse Treatment, 9, 199-213
71. Belperio PS, Rhew DC. Prevalence and outcomes of anemia in individuals with human immunodeficiency virus: a systematic review of the literature. Am J Med. 2004 Apr 5;116 Suppl 7A:27S-43S.

72. Means RT Hepcidin and cytokines in anaemia. Hematology. 2004 Oct-Dec;9(5-6):357-62.
73. Simon R, Wittes R, & Ellenberg S. (1985) Randomized Phase II Clinical Trials. Cancer Treatment Reports 69 (12): 1375-1381.
74. Steinberg S, & Venzon D. (2002) Early selection in a randomized phase II clinical trial. Statistics in Medicine 21: 1711-1726.
75. Morgan D, Ross A, Mayanja B, Malamba S, Whitworth J. (1998) Early manifestations (pre-AIDS) of HIV-1 infection in Uganda. AIDS 12: 591-596.
76. Rodriguez B, Sethi A, Cheruvu V, Mackay W, Bosh R, Kitahata M, Boswell S, Mathews W, Bangsberg D, Martin J, Whalen C, Sieg S, Yadavalli S, Deeks S, Lederman M. Predictive value of plasma HIV RNA level on rate of CD4 T-Cell decline in untreated HIV infection. 2006 JAMA 296(12):1498-1506.

14 APPENDICES

14.1 Declaration of Helsinki

14.2 Clinical Trial Compensation Guidelines

14.3 Sample Patient Information Leaflet and Informed Consent Form

14.4 Pill Count for Adherence Assessment

14.5 Product Information A (http://www.sutherlandia.org/index.html

14.6 Product Information B (Background: www.sutherlandia.org/chemistry.html)

14.7 Product Information C (Supplier)

14.8 Product Information D (Package Insert Equivalent)

14.9 Product Information E (Label)

14.10 Study Questionnaires and Measurement Instruments

- **Validation of the World Health Organization Quality of Life Instrument in patients with HIV infection**
- **Center for Epidemiologic Studies Depression (CES-D)**
- **Perceived Stress Scale**
- **ACTG Adherence Baseline Questionnaire**
- **ACTG Adherence Follow Up Questionnaire**
- **The Addiction Severity Index – Lite (ASI-Lite)**
- **Addiction Severity Index Lite - CF**
